# Chromosome condensation mechanically primes the nucleus for mitosis

**DOI:** 10.1038/s44318-026-00835-8

**Published:** 2026-06-29

**Authors:** Vanessa Nunes, Margarida Moura, Sara F Silva, Débora Vareiro, Nicolas Audugé, Nicolas Borghi, Jorge G Ferreira

**Affiliations:** 1 Instituto de Investigação e Inovação em Saúde (i3S), Universidade do Porto, Porto, Portugal; 2 Programa Doutoral em Biomedicina, Faculdade de Medicina do Porto, Universidade do Porto, Porto, Portugal; 3 Université Paris-Cité, CNRS, Institut Jacques Monod, Paris, France; 4 Departamento de Biomedicina, Faculdade de Medicina do Porto, Universidade do Porto, Porto, Portugal; 5 Present Address: Institut Curie, Paris, France

**Keywords:** Cell Cycle, Chromatin, Transcription & Genomics

## Abstract

Accurate transition into mitosis driven by cyclin B1-CDK1 activity is essential to avoid chromosome segregation errors and preserve genome integrity. How this activity is spatially controlled to trigger mitotic onset remains unclear. Here, we show that chromosome condensation triggers an increase in nuclear envelope (NE) tension. This increased tension is required for translocation of cyclin B1 into the nucleus and dynein loading on nuclear pore complexes (NPCs), ensuring timely mitotic entry. Micromanipulation experiments further indicate this tension-dependent mechanism requires SUN proteins on the NE. Impairment of chromosome condensation leads to the nuclear accumulation of the G2 checkpoint kinase Wee1 and an inhibition of CDK1 activity, which result in a temporary delay in mitotic entry. This delay can be overridden by increasing tension on the NE, which accelerates the nuclear translocation of cyclin B1 and dynein loading. We propose that mitotic onset is controlled by a chromosome-dependent NE tension mechanism that enables robust spatiotemporal coupling between chromosome condensation and the NE structural changes required for an efficient mitosis.

## Synopsis

Nuclear translocation of cyclin B1-CDK1 during prophase ensures irreversible mitotic commitment. This study shows that chromosome condensation generates tension on the nuclear envelope (NE), regulating cyclin B1 nuclear translocation and mitotic onset.

- Mitotic chromosome condensation increases NE tension during prophase.
- Chromosome-dependent tension regulates the nuclear translocation of cyclin B1 and dynein loading on nuclear pore complexes, leading to mitotic entry.
- Tension-dependent mitotic entry requires SUN proteins on the inner nuclear membrane.
- This mechanism ensures robust coordination between chromosome condensation and mitotic entry.

## Abstract (web summary)

Nuclear envelope tension generated by chromosome condensation regulates cyclin B1 nuclear translocation and mitotic onset.

## Graphical abstract

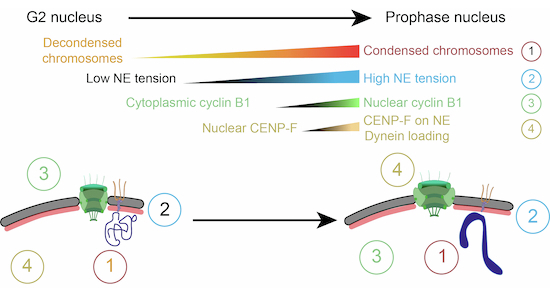

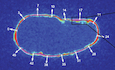

## Introduction

Mitosis is a highly regulated process, essential for organism development and homeostasis. A precise spatiotemporal control of mitosis ensures an accurate partitioning of chromosomes, preventing segregation errors and aneuploidy (Dantas et al, 2022; Furuno et al, 1999; Thompson and Compton, 2008). Underlying this spatiotemporal regulation is an intricate biochemical pathway, controlled by a complex formed by cyclin B1 and its associated cyclin-dependent kinase (CDK) 1 (Lindqvist et al, 2009), that translocates to the nucleus (Gavet and Pines, 2010a) during the transition from G2 to mitosis (G2-M). Once inside the nucleus, this complex performs a series of well-characterized phosphorylations on key nuclear substrates that result in chromosome condensation (Abe et al, 2011), nuclear pore complex (NPC) dismantling (Linder et al, 2017) and nuclear lamina disassembly (Heald and McKeon, 1990), ultimately triggering nuclear envelope permeabilization (NEP) (Lindqvist et al, 2009) and irreversible mitotic commitment. Therefore, cyclin B1-CDK1 was proposed to coordinate the cytoplasmic and nuclear events required for timely mitotic entry (Gavet and Pines, 2010a; Gavet and Pines, 2010b). Nevertheless, the signal that triggers the initial nuclear accumulation of the complex, and therefore determines the timing of mitotic onset, remains elusive.

The disassembly of the nuclear envelope (NE) during the initial stages of cell division (Champion et al, 2017) gives rise to an “open mitosis”, characteristic of mammalian cells. This process requires the removal of NE membranes by dynein-mediated microtubule (MT) forces (Beaudouin et al, 2002; Salina et al, 2002). Dynein associates with the NE during early prophase by interacting with specific NPC components. It can either bind Nup358/RanBP2 through its interaction with BicD2 (Splinter et al, 2010) or associate with Nup133 via its binding to CENP-F/NudE/EL (Bolhy et al, 2011). Importantly, CENP-F, one of the key components of the Nup133 pathway, is exported from the nucleus in a CDK1-dependent manner during the G2-M transition to associate with NPCs and enable dynein loading (Berto and Doye, 2018; Bolhy et al, 2011; Loftus et al, 2017). Therefore, during the G2-M transition, nuclear-cytoplasmic transport must be tightly regulated to ensure the ordering of early mitotic events.

The nucleus has been traditionally viewed as merely a structure that needs to be disassembled at mitotic entry. However, accumulating evidence reveals that multiple nuclear components play critical roles in regulating mitotic timing and fidelity (Belaadi et al, 2022; Champion et al, 2019; Dantas et al, 2022; Lima et al, 2024; Turgay et al, 2014). Still, how these nuclear components mechanistically contribute to mitosis is poorly understood. Here, we investigated how the NE regulates mitotic onset. We show that mitotic chromosome condensation during prophase generates NE tension which, in turn, coordinates the cytoplasmic and nuclear events required for timely mitotic entry.

## Results

### The nucleus is remodelled during mitotic entry

**Figure 1:**
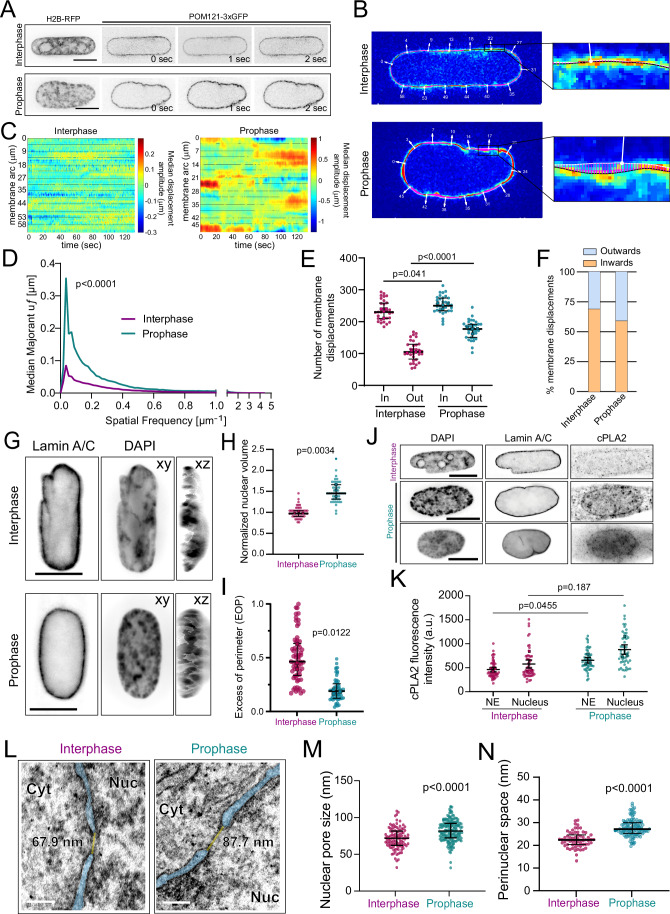
Nuclear remodelling during the G2-M transition. (**A**) Selected frames from movies of HeLa cells expressing POM121-3xGFP/H2B-RFP, used for NE fluctuation analysis. Identification of prophase cells was done by assessing chromosome condensation with the H2B-RFP signal. (**B**) Representative examples of inwards (pink vectors) or outwards (red vectors) NE membrane displacements of a cell in interphase (top) and a cell in prophase (bottom). (**C**) Map of the membrane displacement amplitude, u, obtained for all time frames. The *y* axis corresponds to the arc around the median NE membrane. (**D**) Median of the majorant of frequency-dependent displacements, *uf*, obtained by finding the maximum amplitude of the spatial Fourier transform (FT) for each frequency for interphase (*n* = 32 cells from 4 independent replicates) and prophase cells (*n* = 32 cells from 4 independent replicates; *P *< 0.0001, using a two-way ANOVA). This FT curve shows the maximum displacement amplitude of each wavelength. Quantification of the orientation (**E**) and proportion (**F**) of NE displacements for interphase (*n* = 32 cells from four independent replicates) and prophase cells (*n* = 32 cells from 4 independent replicates). “Out” corresponds to outwards-directed movements and “In” corresponds to inwards-directed movements (*P* = 0.041 for inwards displacements, using an unpaired *t* test; *P* < 0.0001 for outwards displacements, using an unpaired *t* test). (**G**) Representative immunofluorescence images of nuclei labelled for Lamin A/C and stained with DAPI highlighting xy and xz projections. (**H**) Quantification of nuclear volume for cells in interphase (*n* = 51 cells, 3 replicates) and prophase (*n* = 50 cells, 3 replicates; *P* = 0.0034, using an unpaired *t* test). (**I**) Estimation of nuclear folding as assessed by the excess of perimeter (EOP) parameter for interphase (*n* = 51 cells, 3 replicates) and prophase cells (*n* = 50 cells, 3 replicates; *P* = 0.0122, using an unpaired *t* test). (**J**) Representative immunofluorescence images of nuclei labelled for cPLA2 and Lamin A/C and stained with DAPI. (**K**) Quantification of cPLA2 fluorescence intensity on the NE and nucleus of interphase (*n* = 55 cell, 3 replicates) and prophase cells (*n* = 50 cells, 3 replicates; *P* = 0.0455 for NE, using an unpaired *t* test; *P* = 0.187 for nucleus, using an unpaired *t* test). (**L**) Representative transmission electron microscopy (TEM) images of nuclear boundary regions in interphase and prophase. The NE is highlighted in blue and NPCs in yellow. Quantification of NPC size ((**M**); *P* < 0.0001, using a Mann–Whitney test) and perinuclear space (PNS; (**N**); *P* < 0.0001, using a Mann–Whitney test) for interphase (*n* = 11 cells; 106 NPCs) and prophase cells (*n* = 18 cells; 182 NPCs). On the dot plots, each dot corresponds to an individual value, the line corresponds to the median and the bars correspond to the interquartile range. Square dots represent the median of each replicate in the experiment.

**Figure EV1:**
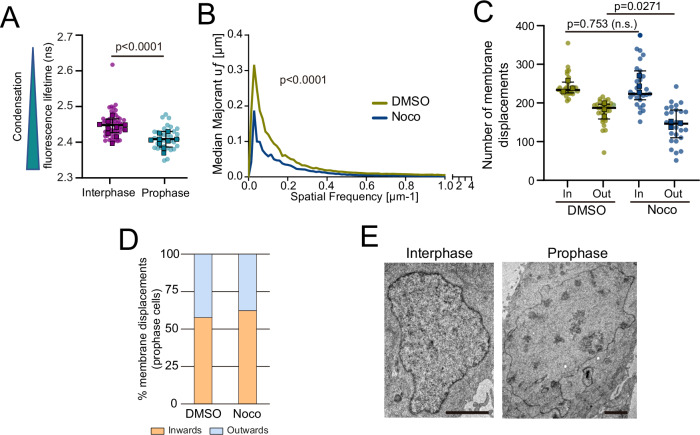
Microtubule-dependent changes in NE membrane displacement. (**A**) Fluorescence lifetime microscopy (FLIM) measurement of histone H4-GFP of cells in interphase (*n* = 50 cells, 3 replicates) and prophase (*n* = 36 cells, 3 replicates, *P* < 0.0001, using a Kruskal–Wallis test). Decreased fluorescence lifetime in prophase cells reflects increased chromosome compaction. (**B**) Median of the majorant of frequency-dependent NE membrane displacements, uf, obtained by finding the maximum amplitude of the spatial Fourier transform (FT) for each frequency for DMSO (*n* = 30 cells, 3 replicates) and Nocodazole treated cells (Noco; *n* = 27 cells, 3 replicates; *P* < 0.0001, using a two-way ANOVA). This FT curve shows the maximum displacement amplitude of each wavelength. Quantification of the orientation (**C**) and proportion (**D**) of NE membrane displacements for DMSO (*n* = 30 cells, 3 replicates) and Nocodazole treated cells (*n* = 27 cells, 3 replicates; *P* = 0.0271, using an unpaired *t* test). (**E**) Representative TEM images of interphase (left) and prophase (right) nuclei. On the dot plots, each dot corresponds to an individual value, the line corresponds to the median and the bars correspond to the interquartile range. Square dots represent the median of each replicate in the experiment. Scale bars correspond to 5 μm.

During the G2-M transition, the cell nucleus undergoes extensive remodelling (Champion et al, 2017) to ensure an efficient assembly of the mitotic spindle (Lancaster et al, 2013). Whether these changes impact nuclear morphodynamics is not known. To address this, we used a combination of quantitative live-cell microscopy with micromanipulation in cells during late G2/prophase. These cells were readily identified by the presence of condensed chromosomes (Fig. 1A), one of the first signs of mitotic commitment (McIntosh, 2016). Using a parallel approach, we confirmed prophase chromosome condensation by performing fluorescence lifetime imaging (FLIM) of histone EGFP-H4. This revealed that prophase cells had lower lifetime measurements than interphase cells (Fig. EV1A), reflecting an increased chromosome compaction (Auduge et al, 2019). Then, to determine if mitotic chromosome condensation correlated with changes in the physical state of the nucleus, we measured NE membrane displacements across the entire NE, in HeLa cells expressing POM121-3xGFP/H2B-RFP (Castro et al, 2020). These cells were seeded in line micropatterns to standardize nuclear shape and imaged with high temporal resolution (200 msec), using spinning disk microscopy (Fig. 1A). Notably, prophase cells showed increased membrane displacements across the entire NE (Fig. 1B–D), when compared to their interphase counterparts. Moreover, most of these membrane movements were directed towards the nuclear interior (Fig. 1E,F), suggesting they might be dependent on inwards-directed active forces imposed on the NE (Introini et al, 2023; Schreiner et al, 2015). Next, since MT-generated forces were previously shown to trigger changes in NE dynamics in interphase cells (Hampoelz et al, 2011), we disrupted the MT network by treating cells with nocodazole (Noco). Accordingly, although noco-treated cells presented a significant dampening of the overall NE movements (Fig. EV1B), their inwards orientation was not significantly altered when compared to control cells treated with DMSO (Fig. EV1C,D). Together, these results indicate that the magnitude of NE movements during prophase partly depends on forces exerted by MTs, but their inwards orientation does not.

Additional nuclear alterations were observed as cells prepared to divide. Our analysis revealed a significant increase in nuclear volume (Fig. 1G,H), which correlated with an unfolding of the NE as reflected by a decrease in the excess of perimeter (EOP) parameter in prophase cells (Fig. 1G,I). Here, increased values of EOP reflect a higher degree of NE folding. Considering that both phenomena are indicative that, during the G2-M transition, the NE is under increased tension, we next measured the levels of the cytosolic phospholipase A2 (cPLA2) in both interphase and prophase cells (Fig. 1J,K). Interestingly, our analyses revealed a recruitment of cPLA2 to the NE of prophase cells, which normally occurs when the NE is under tension (Dantas et al, 2022; Enyedi et al, 2016; Lomakin et al, 2020; Venturini et al, 2020). Previous studies have shown that imposing tension on the nucleus is sufficient to deform NPCs (Schuller et al, 2021; Zimmerli et al, 2021), leading to faster nuclear transport (Andreu et al, 2022; Elosegui-Artola et al, 2017). Moreover, we have previously demonstrated that nuclear tension during prophase can determine the timing of mitotic entry by regulating cyclin B1 translocation across the NPCs (Dantas et al, 2022). Therefore, we determined whether this increased nuclear volume and NE tension during prophase impacts NPC deformation. Comparison of NPC diameter between interphase cells and cells that were synchronized in G2 with a double thymidine block and subsequently released into prophase, indicated that synchronized cells present a significant dilation of NPCs (Figs. 1L,M and EV1E), which was accompanied by an increase in the perinuclear space (PNS; Fig. 1L,N). Together, these data demonstrate that a nuclear remodelling takes place during the G2-M transition.

### Chromosome condensation ensures the timing of mitotic entry

**Figure 2:**
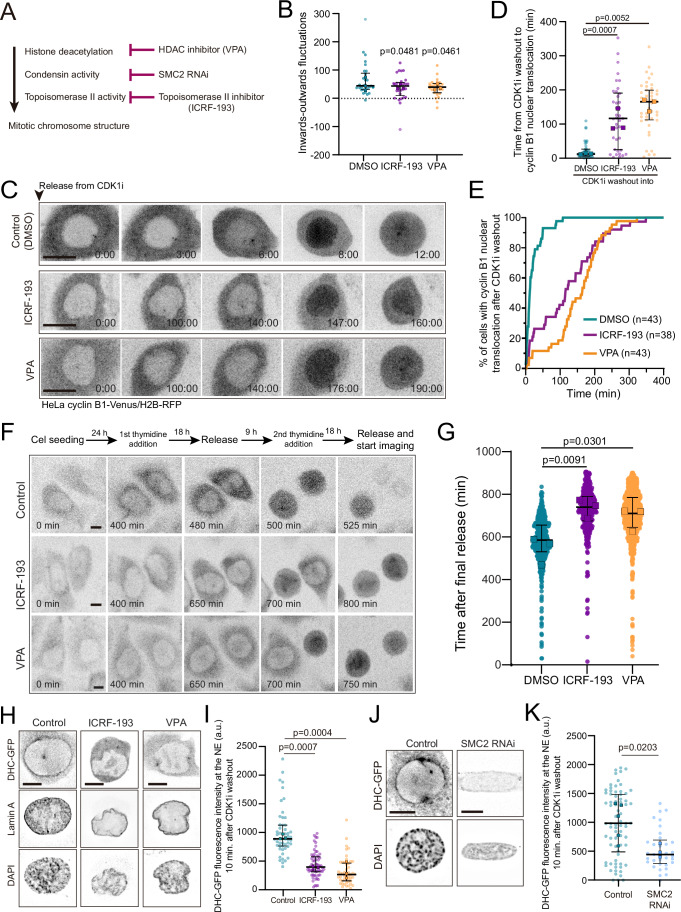
Chromosome condensation is required for timely mitotic entry. (**A**) Scheme with the multiple strategies employed to interfere with mitotic chromosome structure. (**B**) Quantification of the orientation bias in NE fluctuations for control cells (DMSO; *n* = 30, 3 replicates) and cells treated with either ICRF-193 (*n* = 29, 3 replicates; *P *= 0.0481) or VPA (*n *= 27, 3 replicates; *P* = 0.0461). Comparisons between groups were performed using an ANOVA test. Values above zero reflect a bias towards inwards fluctuations. (**C**) Representative frames from movies of cells expressing cyclin B1-Venus/H2B-RFP treated with DMSO (top panel), ICRF-193 (middle panel) or VPA (bottom panel). Time is represented in minutes (min) and zero corresponds to the moment of CDK1i washout. (**D**) Quantification of the time for cyclin B1 nuclear translocation after CDK1i washout to for the indicated treatments (*n* = 43 cells, 3 replicates for DMSO; *n* = 38 cells, 3 replicates for ICRF-193, *P* = 0.0007; *n* = 43 cells, three replicates for VPA; *P* = 0.0052). Comparisons between groups were performed using an ANOVA test. (**E**) Cumulative percentage of cells with cyclin B1 nuclear translocation following CDK1i washout. (**F**) Representative frames from movies of cells expressing cyclin B1-Venus after double thymidine synchronization treated with DMSO (top panel), ICRF-193 (middle panel) or VPA (bottom panel). Time lapse is 5 min. Time is in min. (**G**) Quantification of the time from thymidine washout to cyclin B1 translocation for DMSO (*n* = 680 cells, 3 replicates), ICRF-193 (*n* = 356 cells, 3 replicates, *P* = 0.0091) or VPA (*n* = 902 cells, 3 replicates, *P* = 0.0301). Comparisons between groups were performed using an ANOVA test. (**H**) Representative immunofluorescence images of prophase cells expressing dynein heavy chain (DHC)-GFP after treatment with DMSO, ICRF-193 or VPA. (**I**) Quantification of the levels of DHC-GFP fluorescence intensity on the NE for DMSO (*n* = 52 cells, 3 replicates), ICRF-193 (*n* = 63 cells, 3 replicates; *P* = 0.0007) or VPA (*n* = 49 cells, 3 replicates, *P* = 0.0004) treated cells. Comparisons between groups were performed using an ANOVA test. (**J**) Representative immunofluorescence images of DHC-GFP localization at the NE, following depletion of SMC2 by RNAi. (**K**) Quantification of the levels of DHC-GFP on the NE following for control cells (*n* = 74 cells, 5 replicates) or SMC2-depleted cells (SMC2 RNAi; *n* = 33 cells, 3 replicates; *P* = 0.0203, using an unpaired *t* test). On the dot plots, each dot corresponds to an individual value, the line corresponds to the median and the bars correspond to the interquartile range. Square dots represent the median of each replicate in the experiment. Scale bars, 10 μm.

**Figure EV2:**
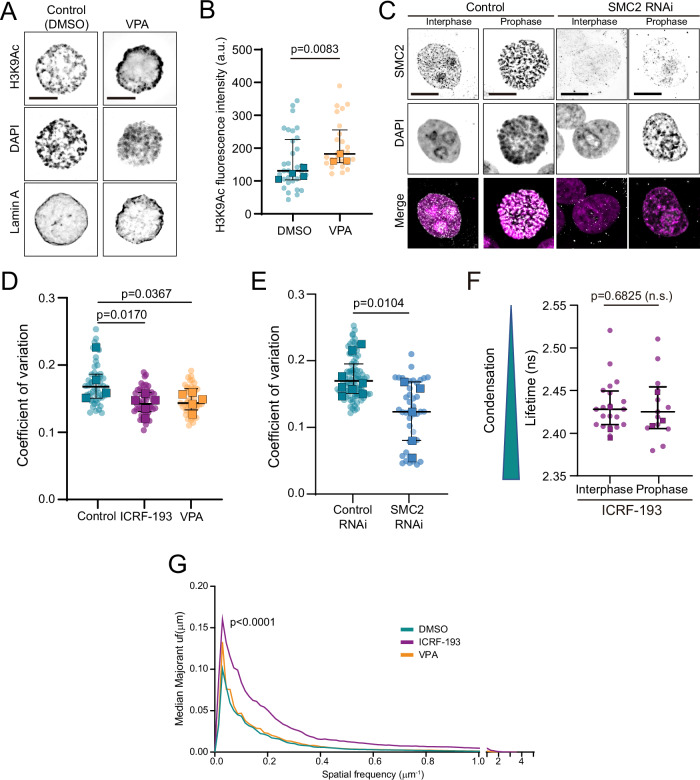
Disrupting chromosome condensation affects NE membrane displacements. (**A**) Representative immunofluorescence images of histone H3 acetylation on lysine 9 (H3K9ac) in control cells (DMSO) and VPA-treated cells. (**B**) Quantification of H3K9ac levels in prophase cells treated with DMSO (*n* = 33 cells, 4 replicates) or VPA (*n* = 25 cells, 3 replicates for VPA; *P* = 0.0083, using an unpaired *t* test), to determine the efficacy of VPA treatment in increasing histone acetylation. (**C**) Representative images of SMC2 immunostaining in control cells and SMC2-depleted cells in interphase and prophase, to demonstrate the efficacy of the RNAi treatment. (**D**) Quantification of chromosome condensation using the coefficient of variation (CV) parameter. Treatment with ICRF-193 (*n* = 49 cells, 5 replicates, *P* = 0.0170) or VPA (*n* = 48 cells, 5 replicates, *P* = 0.0367) significantly decreases the CV when compared to control cells (*n* = 52 cells, 4 replicates), reflecting an impairment in chromosome condensation. Comparisons between groups were performed using an ANOVA test. (**E**) Quantification of chromosome condensation after depletion of SMC2 by RNAi. The CV is significantly decreased in depleted cells (*n* = 42 cells, 5 replicates; *P* = 0.0104, using an unpaired *t* test) when compared to control cells (*n* = 91 cells, 8 replicates). (**F**) FLIM measurement of histone H4-GFP in interphase or prophase cells treated with ICRF-193 (*n* = 20 cells for interphase and *n* = 13 cells for prophase; p = 0.6825 using a Mann–Whitney test). Decreased fluorescence lifetime corresponds to increased chromosome compaction. (**G**) Median of the majorant of frequency-dependent NE membrane displacements, uf, obtained by finding the maximum amplitude of the spatial Fourier transform (FT) for each frequency for DMSO (*n* = 27 cells), ICRF-193 (*n* = 30 cells) or VPA-treated cells (*n* = 22 cells; *P* < 0.0001, using a Friedman test). This FT curve shows the maximum displacement amplitude of each wavelength. On the dot plots, each dot corresponds to an individual value, the line corresponds to the median and the bars correspond to the interquartile range. Square dots represent the median of each replicate in the experiment.

We then set out to determine the mechanism behind this prophase nuclear remodelling. We hypothesized that the remodelling might depend on mitotic chromosome condensation, since this could be an early source of mechanical perturbation of the NE as cells prepare to enter mitosis. To test this, we disrupted mitotic chromosome structure using well-established methodologies (Fig. 2A). We interfered with topoisomerase II (TopoII) activity using ICRF-193 (Ishida et al, 1994), inhibited the activity of histone deacetylases (HDAC) using valproic acid (VPA; Fig. EV2A,B) or depleted condensins using RNAi (SMC2 RNAi; Fig. EV2C) (Schneider et al, 2022). Efficiency of these treatments in disrupting mitotic chromosome structure was assessed by analysing the coefficient of variation (CV) of chromatin (Neguembor et al, 2021). Notably, the treatments resulted in a significant decrease in the CV for the drugs (Fig. EV2D), as well as SMC2 RNAi (Fig. EV2E), indicating a disruption in chromosome condensation. FLIM measurements on EGFP-H4 confirmed the effect of ICRF-193, which abolished the decrease in fluorescence lifetime between interphase and prophase (Fig. EV2F). Having established the efficacy of these treatments in disrupting mitotic chromosome condensation, we then asked whether they could lead to changes in NE morphodynamics. Treatment with ICRF-193 or VPA induced an increase in median membrane movements across the NE (Fig. EV2G), with a small but significant shift towards outwards-directed motions (Fig. 2B). These changes in NE dynamics indicate that chromosome condensation is likely responsible for the bias towards inward displacements during prophase, and suggest that condensation affects the tension state of the NE.

**Figure EV3:**
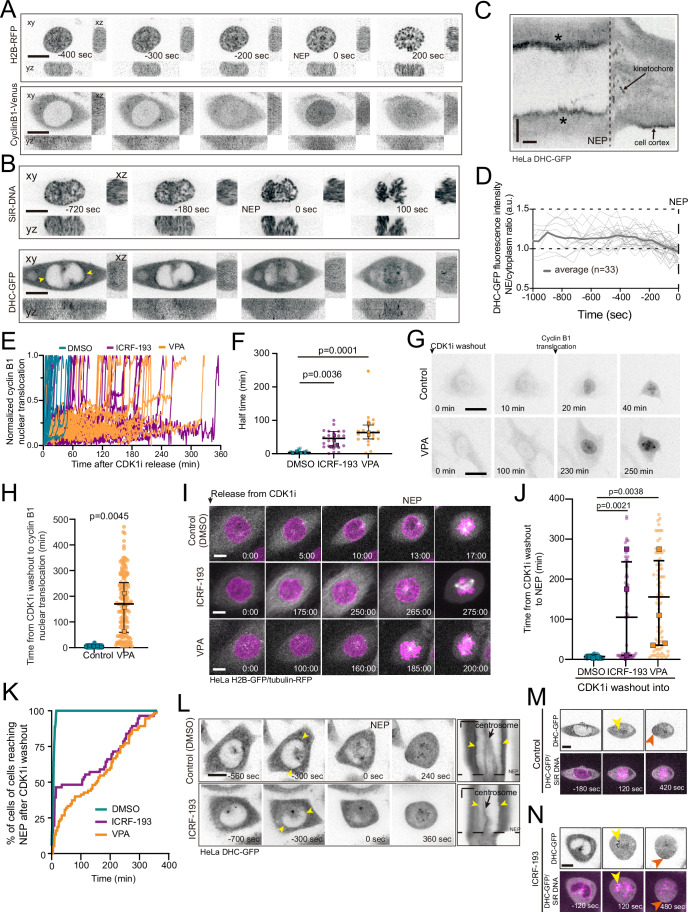
Chromosome condensation sets the timing of mitotic entry. (**A**) Representative frames from a movie of a HeLa cell expressing cyclin B1-Venus/H2B-RFP during mitotic entry. (**B**) Representative frames from a movie of a HeLa cell expressing DHC-GFP and stained with SiR-DNA during mitotic entry. Note the accumulation of dynein on the NE (yellow arrowheads). (**C**) Kymograph of the cell in (**B**), highlighting the accumulation of dynein on the NE prior to NEP (asterisks), as well as its accumulation on kinetochores and cell cortex after mitotic entry. Horizontal scale bar, 200 s. Vertical scale bar, 10 μm. (**D**) Ratio between dynein on the NE and on the cytoplasm over time in prophase cells (*n* = 33 cells). Note how the ratio is above 1, reflecting an enrichment on the NE at this stage. In the movies, time zero corresponds to the moment of NEP. (**E**) Individual traces for cyclin B1 nuclear translocation for cells treated with DMSO, ICRF-193 or VPA. Time zero corresponds to the time of CDK1 inhibitor (CDK1i) washout. Values were normalized and scaled between 0 and 1, corresponding to the minimum and maximum values, respectively. (**F**) Half-times for cyclin B1 translocation were calculated from the curve fits for DMSO, ICRF-193 (*P *= 0.0036) and VPA (*P* = 0.0001). Comparisons between groups were performed using an ANOVA test. (**G**) Representative frames from movies of RPE-1 cells expressing cyclin B1-eYPF after synchronization with a CDK1 inhibitor. Following washout, cells were filmed during mitotic entry. Time lapse is 5 min. Time is in min and scale bars are 100 μm. (**H**) Quantification of the time from CDK1i washout to cyclin B1 nuclear translocation in RPE-1 cells (*n* = 232 cells, 5 replicates for DMSO; *n* = 149 cells, 3 replicates for VPA; *P* = 0.0045, using an unpaired *t* test). (**I**) Representative frames from movies of cells expressing H2B-GFP/tubulin-RFP that were synchronized in late G2 using CDK1i and incubated with DMSO, ICRF-193 or VPA overnight. Following CDK1i washout, cells progressed to prophase, in medium still containing either DMSO, ICRF-193 or VPA. Time zero corresponds to washout of CDK1i. (**J**) Quantification of the time from CDK1i washout to NEP for DMSO (*n* = 70 cells, 9 replicates), ICRF-193- (*n* = 84 cells, 3 replicates, *P* = 0.0021) or VPA-treated cells (*n* = 85 cells, 4 replicates, *P* = 0.0038). Comparisons between groups were performed using an ANOVA test. NEP is determined as the moment soluble tubulin enters the nuclear space, reflecting permeabilization of the NE. (**K**) Cumulative percentage of cells treated with DMSO, ICRF-193 or VPA that enter mitosis (NEP) after CDK1i washout. (**L**) Representative frames of HeLa cells expressing DHC-GFP cells during mitotic entry, after treatment with DMSO (top panel) or ICRF-193 (bottom panel). Yellow arrowheads indicate the NE. Right panels show kymographs highlighting the absence of dynein on the NE of ICRF-193 treated cells. Horizontal scale bar, 10 μm. Vertical scale bar, 200 s. Representative frames from movies of cells expressing DHC-GFP and stained with SiR-DNA from control cells (**M**) and ICRF-193 (**N**) treated cells. Note how dynein still accumulates at kinetochores (yellow arrowhead) and cell cortex (orange arrowhead) after ICRF-193 treatment. On the dot plots, each dot corresponds to an individual value, the line corresponds to the median and the bars correspond to the interquartile range. Square dots represent the median of each replicate in the experiment.

Next, we asked whether perturbations in chromosome condensation could have implications for the timing and efficiency of mitotic entry. In preparation for mitosis, cyclin B1 translocates through the NPCs into the nucleus (Fig. EV3A), a process that depends on importins (Takizawa et al, 1999) and nuclear tension (Dantas et al, 2022). In addition, dynein is loaded on the NE (Fig. EV3B–D) in a CDK1-dependent manner (Baffet et al, 2015). We started by monitoring the nuclear translocation of cyclin B1 (Gavet and Pines, 2010a), as this event is required for NPC dismantling (Laurell et al, 2011), ultimately leading to disassembly of the nuclear lamina and NEP. HeLa cells expressing endogenously tagged cyclin B1-Venus and H2B-RFP were synchronized in late G2 using a CDK1 inhibitor (CDK1i; RO-3306). Following release from the inhibitor, we monitored the translocation of cyclin B1 into the nucleus using high-resolution live-cell imaging, as cells progressed towards mitosis (Fig. 2C; Movie EV1). Notably, disruption of chromosome structure using ICRF-193 or VPA significantly delayed cyclin B1 nuclear accumulation (Figs. 2C–E and EV3E; Movie EV1), with an increase in the half-time of translocation from 4.0 ± 4.3 min in DMSO to 42.2 ± 27.2 min in ICRF-193- (*P* = 0.0036) and 67.4 ± 49.6 min in VPA-treated cells (*P* = 0.0001; Fig. EV3F). To rule out the possibility that the delay in cyclin B1 translocation could be due to the use of HeLa cells, which have a perturbed cell cycle, we decided to use near-diploid, untransformed RPE-1 cells expressing endogenously tagged cyclin B1-eYFP (Akopyan et al, 2014). These cells were treated with VPA, synchronized in late G2 with CDK1i and imaged during mitotic entry (Fig. EV3G). Importantly, treatment of RPE-1 cells with VPA induced a delay in cyclin B1 translocation (Fig. EV3H), confirming our earlier observations in HeLa cells. While we cannot exclude other differences in cell cycle progression, our results indicate that disrupting mitotic chromosome condensation in RPE-1 cells also delays cyclin B1 translocation. Next, since prolonged inhibition of CDK1 could impact the activity of other complexes such as cyclin A-CDK1, we performed a double thymidine synchronization using HeLa cells expressing cyclin B1-Venus (Fig. 2F). Consistent with our previous findings, treatment with ICRF-193 or VPA resulted in a significant delay in cyclin B1 translocation (Fig. 2G), independently of direct CDK1 inhibition. Consequently, as a result of these delays in cyclin B1 translocation, DMSO-treated cells entered mitosis within 16.1 ± 14.1 min after CDK1i washout, whereas both ICRF-193- and VPA-treated cells took on average 121.8 ± 91.7 min and 150.5 ± 72.3 min to enter mitosis, respectively (Fig. EV3I–K). Although we cannot exclude additional side-effects of these treatments, it is unlikely that they would all result in similar delays in mitotic entry, independently of their role in chromosome condensation. Therefore, we conclude that the timing of mitotic entry correlates with the degree of chromosome condensation.

To further confirm the contribution of chromosome condensation for mitotic entry, we then evaluated the dynamics of dynein loading on the NE. This process occurs by the binding of dynein to NPCs (Bolhy et al, 2011; Splinter et al, 2010) and is required for efficient NEP (Beaudouin et al, 2002; Salina et al, 2002) and early mitotic spindle assembly (Lima et al, 2024; Nunes et al, 2020). HeLa cells expressing dynein heavy chain (DHC) tagged with GFP were treated with either DMSO or ICRF-193 overnight and imaged during the G2-M transition (Fig. EV3L). In control prophase cells, dynein accumulated on centrosomes and the NE (top kymograph, black and yellow arrows, respectively). Following NE disassembly, dynein relocalized to kinetochores and the cell cortex (Fig. EV3M, yellow and orange arrowheads, respectively). Strikingly, after treatment with ICRF-193, dynein no longer localized to the NE (Fig EV3L, bottom kymograph, yellow arrowheads), although centrosome, kinetochore and cortical localization were unaffected (Fig. EV3L,N). These results suggest that localization of dynein on the NE is dependent on the condensation state of chromosomes. To validate these observations, we quantified the recruitment of DHC-GFP to the NE of prophase cells following ICRF-193 or VPA treatment (Fig. 2H,I), or depletion of SMC2 by RNAi (Fig. 2J,K). HeLa cells were first synchronized in late G2 with CDK1i, released into prophase and fixed 10 min later. Our results show that interfering with mitotic chromosome structure leads to a significant decrease in dynein NE localization, when compared to control cells (Fig. 2H–K). Overall, these results demonstrate that chromosome condensation regulates the timing of key events required for mitotic entry.

### NE tension during prophase depends on chromosome condensation

**Figure 3:**
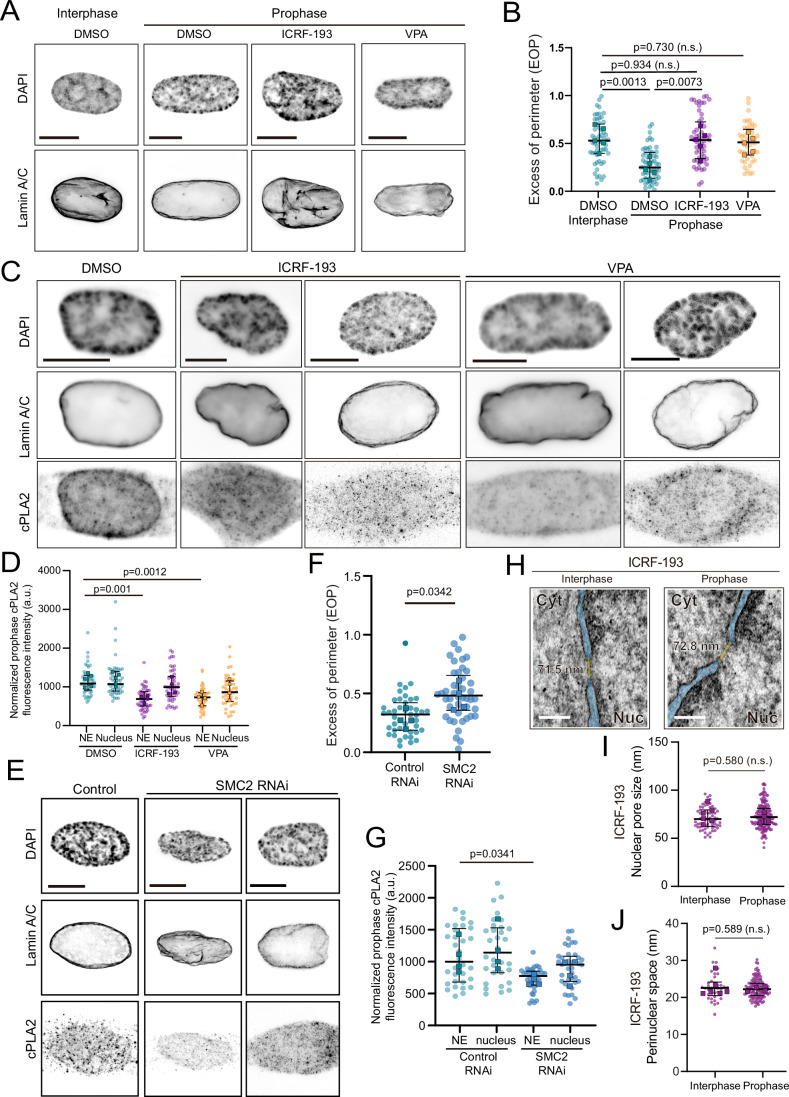
Nuclear envelope tension is determined by chromosome condensation. (**A**) Representative immunofluorescence images of the NE of prophase cells following disruption of chromosome structure. (**B**) Quantification of the excess of perimeter (EOP) in nuclei of interphase (*n* = 59 cells, 5 replicates) and prophase (*n* = 56 cells, 5 replicates) cells treated with DMSO, as well as prophase cells treated with ICRF-193 (*n* = 56 cells, 5 replicates, *P* = 0.0073) or VPA (*n* = 49 cells, 3 replicates; *P *= 0.0163). Multiple comparisons between groups were performed using an ANOVA test. (**C**) Representative immunofluorescence images of nuclei stained for cPLA2 in control cells (DMSO, *n* = 53 cells, 5 replicates), and cells treated with ICRF-193 (*n* = 52 cells, 4 replicates) or VPA (*n* = 46 cells, 4 replicates) in prophase. (**D**) Quantification of cPLA2 levels in the NE and nucleus of DMSO (*n* = 53 cells, 5 replicates), ICRF-193 (*n* = 52 cells, 4 replicates; *P* = 0.001) or VPA (*n* = 46 cells, 4 replicates; *P* = 0.0012) treated prophase cells. Comparisons between groups were performed using an ANOVA test. (**E**) Representative immunofluorescence images of nuclei stained for cPLA2 in control (scramble RNAi) or SMC2 depleted cells (SMC2 RNAi). (**F**) Quantification of the EOP in nuclei of prophase cells treated with control (*n* = 43 cells, 4 replicates) or SMC2 RNAi (*n* = 45 cells, 4 replicates; *P* = 0.0342, using an unpaired *t* test). (**G**) Quantification cPLA2 in the NE (*P* = 0.0341, using an unpaired *t* test) and nucleus of cells depleted of SMC2 by RNAi (*n* = 45 cells, 4 replicates). (**H**) Representative TEM images of the NE of cells treated with ICRF-193 in interphase (left panel) and prophase (right panel), highlighting the NE in blue and NPCs in yellow. Quantification of the nuclear pore size ((**I**); *P* = 0.580, using an unpaired *t* test) and perinuclear space ((**J**); *P* = 0.589, using an unpaired *t* test) in interphase (*n* = 6 cells; 67 NPCs) and prophase cells treated with ICRF-193 (*n* = 17 cells; 168 NPCs). On the dot plots, each dot corresponds to an individual value, the line corresponds to the median and the bars correspond to the interquartile range. Square dots represent the median of each replicate in the experiment. Scale bars in immunofluorescence images, 10 μm. Scale bars in TEM images, 100 nm.

We then set out to determine how chromosome condensation triggers the nuclear remodelling required for timely mitotic entry. During prophase, the nucleus unfolds and swells (Fig. 1G–I) leading to increased NE tension (Dantas et al, 2022). This increased tension regulates the dynamics of cyclin B1 nuclear translocation, setting the time for mitotic entry (Dantas et al, 2022). Based on this report and our current observations that cyclin B1 translocation (Fig. 2C–E) and NE dynein loading (Fig. 2H–K) are impaired upon disruption of chromosome structure, we hypothesized that NE tension could be dependent on efficient chromosome condensation. To test this hypothesis, we treated cells with ICRF-193 or VPA and used the EOP parameter to estimate the extent of NE folding (Dantas et al, 2022; Lomakin et al, 2020). As can be observed, both treatments induced a significant increase in EOP values of prophase nuclei, when compared to controls (Fig. 3A,B). As a result, these were now indistinguishable from nuclei of interphase cells (Fig. 3B). Next, we evaluated the impact of treatments on NE tension. To assess this, we measured the nuclear levels of cPLA2, as these reflect increased tension on the NE of both interphase (Enyedi et al, 2016; Lomakin et al, 2020; Venturini et al, 2020) and prophase cells (Dantas et al, 2022). Both treatments significantly decreased cPLA2 levels on the NE of prophase cells (Fig. 3C,D), when compared to controls. These results were corroborated in cells treated with SMC2 RNAi. Indeed, depleting condensin significantly increased the EOP (Fig. 3E,F), with a corresponding decrease in the levels of cPLA2 (Fig. 3G). Taken together, these results indicate that interfering with mitotic chromosome condensation impairs NE unfolding and reduces tension during prophase. The question then remains of how these changes might impact the translocation of cyclin B1 across the NPCs. Recent works have shown that NPCs can deform in vivo and are sensitive to tension imposed on the NE (Schuller et al, 2021; Zimmerli et al, 2021), likely resulting in increased nuclear transport (Elosegui-Artola et al, 2017). Consistent with this, we observed that prophase nuclei have an increased NPC diameter (Fig. 1L,M). These observations suggest that mitotic chromosome condensation increases tension on the NE, leading to NPC dilation and faster transport of cyclin B1 to the nucleus. To test this, we synchronized cells in G2 using a double thymidine block and incubated them with either DMSO or ICRF-193 after the second release. These cells were then analysed for NPC dilation and PNS size (Fig. 3H). After treatment with ICRF-193, both NPC diameter and the PNS of prophase nuclei were indistinguishable from those of interphase nuclei (Fig. 3I,J), in contrast to what happens in untreated cells (Fig. 1L–N). Therefore, our results indicate that chromosome condensation impacts both NE structure and the timing of mitotic entry.

### Wee1 is required for the chromosome-dependent delay in mitotic entry

**Figure 4:**
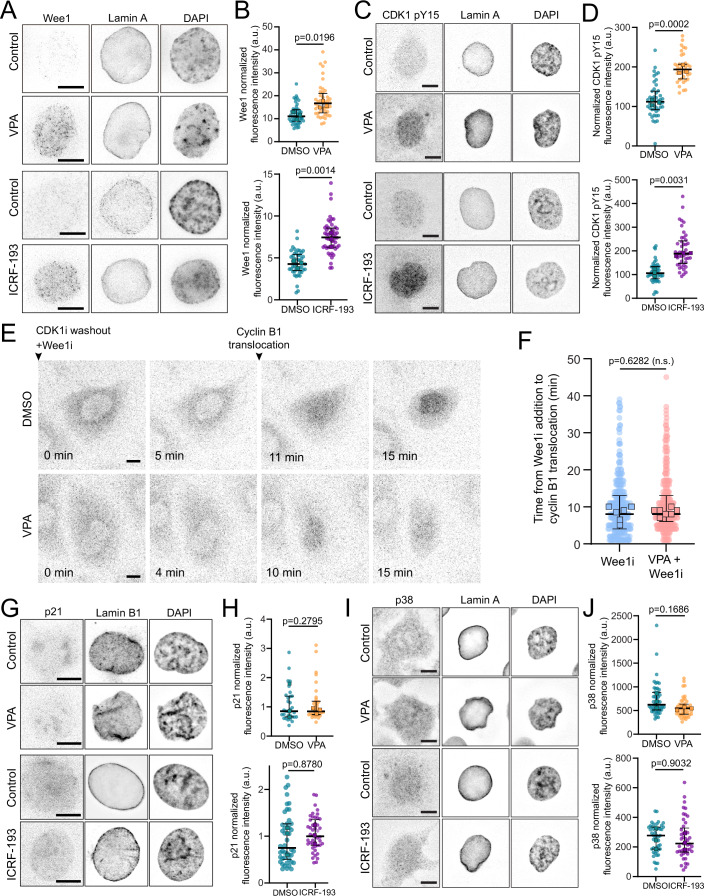
Wee1 delays mitotic entry when chromosome condensation is disrupted. (**A**) Representative immunofluorescence images of cells stained for Wee1, Lamin A and DAPI for control cells and cells treated with ICRF-193 or VPA. (**B**) Quantification of nuclear Wee1 levels in DMSO and VPA-treated cells (top plot; *n* = 49 cells, 3 replicates for DMSO; *n* = 44 cells, 3 replicates for VPA; *P* = 0.0196, using an unpaired *t* test). Quantification of nuclear Wee1 levels in DMSO and ICRF-193 treated cells (bottom plot; *n* = 45 cells, 3 replicates for DMSO; *n* = 52 cells, 3 replicates for ICRF-193; *P* = 0.0014, using an unpaired *t* test). (**C**) Representative immunofluorescence images of cells stained for CDK1 pY15, Lamin A and DAPI for control cells and cells treated with ICRF-193 or VPA. (**D**) Quantification of CDK1 pY15 levels in controls and VPA-treated cells (top plot; *n* = 46 cells, 3 replicates for DMSO; *n* = 39 cells, 3 replicates for VPA; *P* = 0.0002, using an unpaired *t* test). Quantification of CDK1 pY15 levels in controls and ICRF-193 treated cells (bottom plot; *n* = 45 cells, 3 replicates for DMSO; *n* = 45 cells 3 replicates for ICRF-193; *P* = 0.0031, using an unpaired *t* test). (**E**) Representative time frames from movies of HeLa cells expressing cyclin B1-Venus, treated with DMSO or VPA. Cells were synchronized in late G2 using a CDK1 inhibitor (CDK1i, RO-3306). Then, cells were washed out of CDK1i and into medium with a Wee1 inhibitor (Wee1i, MK-1775) and filmed during mitotic entry. (**F**) Quantification of the time from addition of the Wee1 inhibitor to the nuclear translocation of cyclin B1 for control cells (Wee1i only; *n* = 509 cells, 6 replicates) or VPA+Wee1i treated cells (*n* = 485 cells, 7 replicates; *P* = 0.6282, using a Mann–Whitney test). (**G**) Representative immunofluorescence images of cells stained for p21, Lamin B1 and DAPI for controls, ICRF-193 and VPA treated cells. (**H**) Quantification of p21 levels in controls and VPA-treated cells (top plot; *n* = 31 cells, 3 replicates for DMSO; *n* = 31 cells, 3 replicates for VPA, *P *= 0.2795, using an unpaired *t* test). Quantification of p21 levels in control cells, and cells treated with ICRF-193 (bottom plot; *n* = 44 cells, 3 replicates for DMSO, *n* = 46 cells, 3 replicates for ICRF-193, *P* = 0.8780, using an unpaired *t* test). (**I**) Representative immunofluorescence images of cells stained for p38, Lamin A and DAPI for control cells, and cells treated with ICRF-193 or VPA. (**J**) Quantification of p38 levels in DMSO and VPA-treated cells (top plot; *n* = 50 cells, 3 replicates for DMSO, *n* = 47 cells, 3 replicates for VPA, *P* = 0.1686, using an unpaired *t* test). Quantification of p38 levels in DMSO and ICRF-193 treated cells (bottom plot; *n* = 46 cells, 3 replicates for DMSO, *n* = 45 cells, 3 replicates for ICRF-193, *P* = 0.9032, using an unpaired *t* test). On the dot plots, each dot corresponds to an individual value, the line corresponds to the median and the bars correspond to the interquartile range. Square dots represent the median of each replicate in the experiment.

Next, we aimed to understand how interfering with chromosome condensation could result in a delay in mitotic entry. To do so, we analysed potential changes in expression levels and localization of key regulators of the G2-M transition in cells with disrupted chromosome condensation. Immunofluorescence analysis of prophase cells revealed that treatment with VPA or ICRF-193 increased the nuclear levels of Wee1 (Fig. 4A,B), an essential regulator of the G2-M transition (Heald et al, 1993; McGowan and Russell, 1993). Wee1 regulates mitotic entry by phosphorylating CDK1 on its Y15 residue (CDK1 pY15), which blocks CDK1 activity (McGowan and Russell, 1993). Accordingly, immunofluorescence analysis using an antibody to specifically detect the levels of CDK1 pY15 showed that disruption of chromosome condensation correlated with increased levels of this inhibitory phosphorylation (Fig. 4C,D). Overall, these results suggest that Wee1 sustains the delay in mitotic entry observed in cells with disrupted chromosome condensation by maintaining CDK1 in an inactive state. To confirm this, we decided to artificially activate CDK1 by using a specific Wee1 inhibitor (Wee1i; MK-1775). Addition of the Wee1 inhibitor to VPA-treated cells was sufficient to restore the nuclear translocation of cyclin B1 within normal timings (Fig. 4E,F), abolishing the delay observed in cells treated with VPA only (Fig. 2C–E). Importantly, we did not observe changes in the levels of p21 (Fig. 4G,H) or p38 (Fig. 4I,J), suggesting that the observed delay in mitotic entry imposed by disruption of chromosome structure does not depend on the p21-mediated DNA damage checkpoint (Bunz et al, 1998) or the p38-mediated antephase checkpoint (Matsusaka and Pines, 2004), respectively.

### Imposing tension on the NE triggers mitotic entry

**Figure 5:**
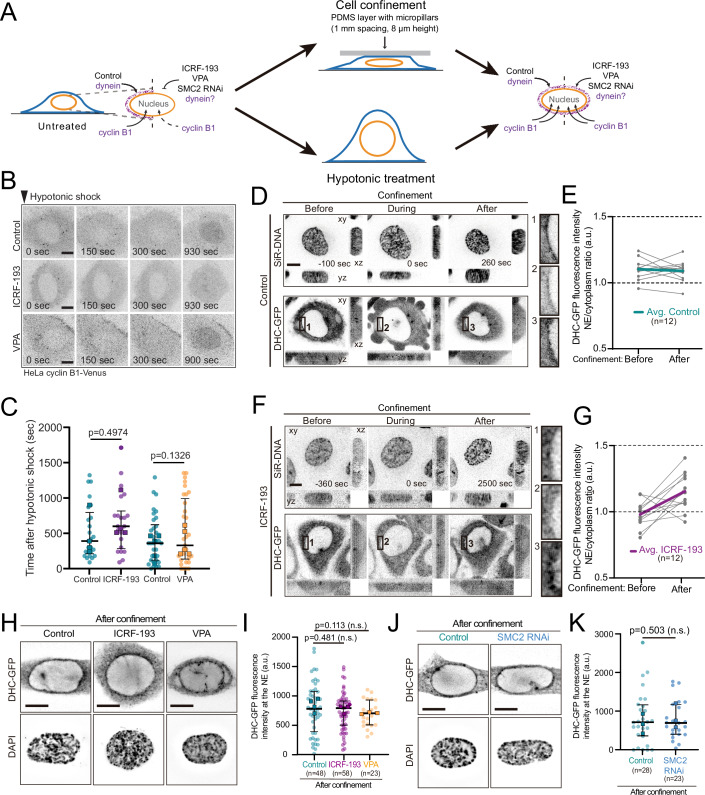
Increased NE tension imposed by chromosome condensation regulates mitotic entry. (**A**) Schematic representation of the experimental setup designed to assess the response of the prophase nucleus to increased tension. (**B**) Representative time frames from movies of cells expressing cyclin B1-Venus and treated with DMSO (top panels), ICRF-193 (middle panels) or VPA (bottom panels). Cells were subjected to hypotonic shock and filmed during mitotic entry. Time is in second. Time lapse is 30 s. (**C**) Quantification of the time from the hypotonic shock to cyclin B1 translocation control cells and cells treated with ICRF-193 (*n* = 25 cells, 4 replicates for control, *n* = 22 cells, 4 replicates for ICRF-193, *P* = 0.4974, using an unpaired *t* test) or VPA (*n* = 34 cells for control, 5 replicates, *n* = 33 cells for VPA, 5 replicates, *P* = 0.1326, using an unpaired *t* test). (**D**) Representative images of control HeLa cells in prophase expressing DHC-GFP and stained with SiR-DNA under mechanical stimulation. Time zero corresponds to the moment when compression was applied to the nucleus. (**E**) Ratio between the intensity of dynein on the NE and cytoplasm for control prophase cells (*n* = 12 cells). Note how the ratio is above 1, reflecting an enrichment of dynein on the NE during this stage of the cell cycle. (**F**) Representative images of a prophase cell treated with ICRF-193 expressing DHC-GFP and stained with SiR-DNA, under mechanical stimulation. Time zero corresponds to the moment when compression was applied to the nucleus. (**G**) Ratio between the intensity of dynein on the NE and cytoplasm for prophase cells treated with ICRF-193 (*n* = 12 cells). Note how the ratio increases following mechanical stimulation. (**H**) Representative immunofluorescence images of DMSO, ICRF-193 or VPA treated cells after confinement, showing localization of dynein on the NE. (**I**) Quantification of DHC-GFP levels on the NE after mechanical stimulation (*n* = 48 cells, 4 replicates for control; *n *= 58 cells, 3 replicates for ICRF-193, *P* = 0.481; *n* = 23 cells, 3 replicates for VPA, *P* = 0.113). Comparisons between groups were performed using an ANOVA test. Note how ICRF-193 and VPA-treated cells accumulate dynein on the NE, similar to control cells. (**J**) Representative immunofluorescence images of control cells (Scramble RNAi) and SMC2 depleted cells (SMC2 RNAi) showing dynein localization on the NE. (**K**) Quantification of DHC-GFP levels on the NE of control cells (*n* = 28 cells, 3 replicates) and cells depleted of SMC2 (*n* = 23 cells, 3 replicates for SMC2 RNAi, *P* = 0.503, using an unpaired *t* test) after mechanical stimulation. On the dot plots, each dot corresponds to an individual value, the line corresponds to the median and the bars correspond to the interquartile range. Square dots represent the median of each replicate in the experiment. Scale bars, 10 μm.

Nuclear mechanics depends on the condensation state of chromatin (Stephens et al, 2017; Stephens et al, 2018) and its tethering to the NE (Schreiner et al, 2015). To test whether chromosome condensation regulates mitotic entry by increasing NE tension, we implemented two parallel approaches using either a cell confinement system or hypotonic treatment (Fig. 5A). Cell confinement induces NE unfolding and cell area expansion (Dantas et al, 2022; Lomakin et al, 2020), while hypotonic treatment results in a swelling of the nucleus and increased volume (Deviri and Safran, 2022). Using these approaches, we tested whether imposing tension on the NE of cells with compromised chromosome condensation was sufficient to restore normal mitotic entry. As a proxy for NE function in prophase, we used cells expressing cyclin B1-Venus to assess the dynamics of cyclin B1 translocation across NPCs, as well as cells expressing DHC-GFP to follow the dynamics of dynein accumulation on the NE, which is sensitive to the condensation state of chromosomes (Fig. 2H–K) and also required for efficient mitotic entry (Beaudouin et al, 2002; Nunes et al, 2020; Salina et al, 2002). In a previous work, we demonstrated that imposing tension on the NE induced a faster nuclear translocation of cyclin B1 (Dantas et al, 2022). Here, we tested whether artificially increasing NE tension could restore the dynamics of cyclin B1 translocation in cells with disrupted chromosome condensation. Indeed, cells that were treated with ICRF-193 or VPA and subjected to hypotonic shock quickly accumulated cyclin B1 in the nucleus (Fig. 5B,C). In addition, transiently increasing NE tension through cell confinement in prophase cells treated with ICRF-193, was sufficient to restore dynein loading on the NE, similarly to control cells (Fig. 5D–G). To extend these observations, we next analysed NE dynein levels in prophase cells that were previously treated with ICRF-193, VPA or SMC2 RNAi, using an immunofluorescence approach. Following treatment, cells were confined for 30 min. After release from the confinement, cells were immediately fixed and processed. Notably, confinement was sufficient to promote NE dynein loading in ICRF-193- and VPA-treated cells (Fig. 5H,I) as well as in cells depleted of SMC2 (Fig. 5J,K). This loading occurred to levels similar to controls, overcoming the block induced by the treatments alone (Fig. 2H–K). These results indicate that increasing NE tension is sufficient to trigger mitotic entry, in conditions where chromosome condensation is disrupted.

### The NE senses chromosome condensation through SUN proteins

**Figure 6:**
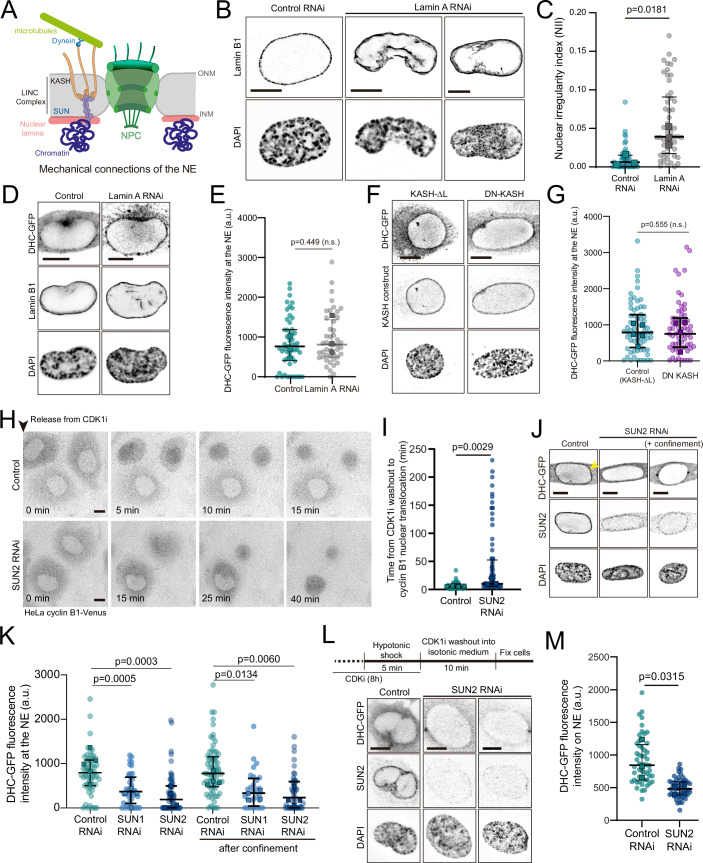
Chromosome condensation is coupled to NE through SUN proteins. (**A**) Schematic depicting the multiple connections of chromatin to the NE. (**B**) Representative immunofluorescence images of cells treated with scrambled RNAi (Control RNAi; *n* = 51 cells, 3 replicates) or Lamin A RNAi (*n* = 53 cells, 3 replicates). (**C**) Quantification of the nuclear irregularity index (NII) of control cells and Lamin A depleted cells (*P* = 0.0181, using an unpaired *t* test). This parameter was calculated as 1-solidity, with solidity being defined as nucleus area/nucleus convex area. (**D**) Representative immunofluorescence images of a cell depleted of Lamin A, showing the localization of dynein on the NE. (**E**) Quantification of dynein on the NE for control (*n* = 53 cells, 3 replicates) or Lamin A depleted cells (*n* = 49 cells, 3 replicates, *P* = 0.449, using an unpaired *t* test). (**F**) Representative immunofluorescence images of cells expressing KASH-ΔL or DN-KASH, showing dynein localization on the NE. (**G**) Quantification of dynein fluorescence intensity on the NE of cells expressing KASH-ΔL (*n* = 68 cells, 5 replicates) or DN-KASH (*n* = 65 cells, 4 replicates, *P* = 0.555, using a Mann–Whitney test). (**H**) Representative time frames of movies from control (top panels) and SUN2 RNAi (bottom panels) HeLa cells expressing cyclin B1-Venus. Cells were synchronized in late G2 with a CDK1 inhibitor. Following inhibitor washout, cells were filmed to monitor cyclin B1 nuclear translocation. Time lapse is 5 min. Time is in min. (**I**) Quantification of the time from CDK1i washout to cyclin B1 nuclear translocation for controls (*n* = 42 cells, 4 replicates) and SUN2 RNAi cells (*n* = 105 cells, 3 replicates; *P* = 0.0029, using an unpaired *t* test). (**J**) Representative immunofluorescence images showing dynein and SUN2 localization on the NE for control cells and SUN2 depleted cells. (**K**) Quantification of dynein levels on the NE for control cells (*n* = 55 cells, 5 replicates), SUN1-depleted cells (*n* = 42 cells, 4 replicates, *P* = 0.0005) and SUN2-depleted cells (*n* = 63 cells, 4 replicates, *P *= 0.0003) without confinement. Comparisons between groups were performed using an ANOVA test. Similar quantifications were performed in control cells (*n* = 69 cells, 4 replicates), SUN1-depleted (*n* = 28 cells, 4 replicates, *P* = 0.0134) and SUN2-depleted (*n* = 45 cells, 4 replicates, *P* = 0.0060) cells that were transiently confined. Comparisons between groups were performed using an ANOVA test. (**L**) Representative immunofluorescence images showing dynein and SUN2 localization. Cells were synchronized with a CDK1i and subjected to a hypotonic shock in the last 5 min. Then, cells were washed out of the inhibitor into isotonic medium. After 10 min, they were fixed and immunostained. (**M**) Quantification of the levels of dynein on the NE for control cells (*n* = 53 cells, 3 replicates) and SUN2 RNAi cells (*n* = 50 cells, 4 replicates, *P* = 0.0315, using an unpaired *t* test). On the dot plots, each dot corresponds to an individual value, the line corresponds to the median and the bars correspond to the interquartile range. Square dots represent the median of each replicate in the experiment. Scale bars, 10 μm.

**Figure EV4:**
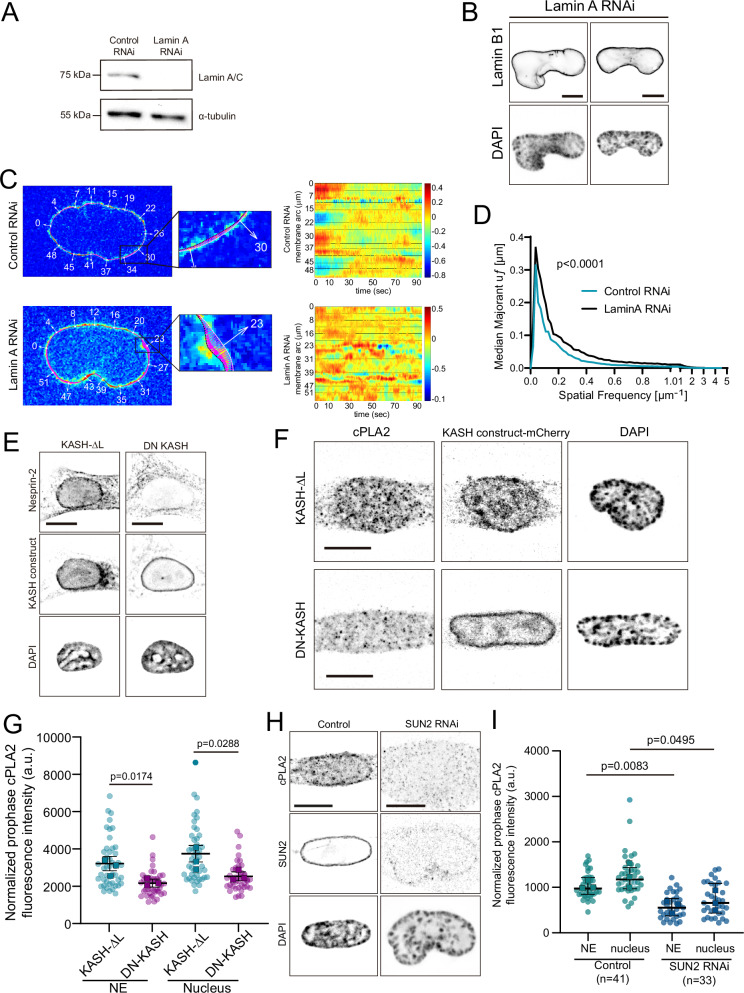
Tension on the NE requires SUN proteins. (**A**) Western blotting analysis of Lamin A levels following depletion by RNAi. (**B**) Representative images of nuclei from cells depleted of Lamin A. (**C**) Representative examples of inwards (pink vectors) or outwards (red vectors) NE membrane displacements of a control cell (top) and a cell depleted of Lamin A (bottom). Right panels show a map of the displacement amplitude, u, obtained for all time frames. The *y* axis corresponds to the arc around the median NE membrane. (**D**) Median of the majorant of frequency-dependent displacements, uf, obtained by finding the maximum amplitude of the spatial Fourier transform (FT) for each frequency for controls (*n* = 24 cells) and Lamin A depleted cells (*n* = 21 cells; *P* < 0.0001, using a two-way ANOVA). This FT curve shows the maximum displacement amplitude of each wavelength. (**E**) Representative immunofluorescence images of the expression of KASH-ΔL (left panel) and DN-KASH (right panel) constructs in HeLa cells and their respective impact on Nesprin-2 localization on the NE. (**F**) Representative immunofluorescence images of cPLA2 levels on the nucleus in cells expressing KASH-ΔL or DN-KASH. (**G**) Quantification of cPLA2 fluorescence intensity on the NE and nucleus for cells expressing KASH-ΔL or DN-KASH (*n* = 46 cells, 3 replicates for KASH-DL; *n* = 42 cells, 3 replicates for DN-KASH, *P* = 0.0174 for NE and *P* = 0.0288 for nucleus). Group comparisons were performed using unpaired *t* tests. (**H**) Representative immunofluorescence images of cPLA2 levels on the nucleus following SUN2 RNAi. (**I**) Quantification of cPLA2 fluorescence intensity on the NE and nucleus for control cells (*n* = 41 cells, 3 replicates) and SUN2 depleted cells (*n* = 33 cells, 3 replicates; *P* = 0.0083 for NE and *P* = 0.0495 for nucleus). Comparisons between groups were performed using unpaired *t* tests. On the dot plots, each dot corresponds to an individual value, the line corresponds to the median and the bars correspond to the interquartile range. Square dots represent the median of each replicate in the experiment. Scale bars, 10 μm.

**Figure EV5:**
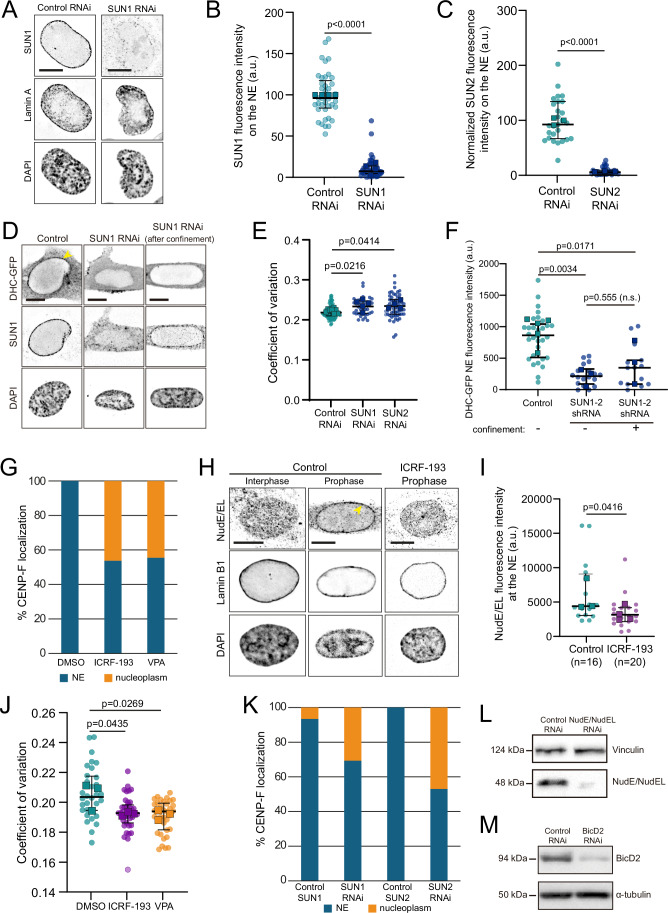
SUN proteins regulate dynein loading through the Nup133-CENP-F pathway. (**A**) Representative immunofluorescence images of SUN1, Lamin A and DAPI for control cells (left panel) and SUN1 depleted cells (right panel). (**B**) Quantification of SUN1 levels on the NE of controls (*n* = 41 cells, 4 replicates) and SUN1 RNAi cells (*n* = 46 cells, 4 replicates, *P* < 0.0001, using an unpaired *t* test). (**C**) Quantification of SUN2 levels on the NE of control cells (*n* = 27 cells, 3 replicates) and SUN2 RNAi cells (*n* = 30 cells, 3 replicates; *P* < 0.0001, using an unpaired *t* test). (**D**) Representative immunofluorescence images showing dynein and SUN1 localization on the NE for control cells and SUN1-depleted cells. (**E**) Analysis of chromosome condensation using the coefficient of variation (CV) for control cells (*n* = 104 cells, 8 replicates) and cells treated with SUN1 RNAi (*n* = 40 cells, 5 replicates; *P* = 0.0216) or SUN2 RNAi (*n* = 60 cells, 4 replicates; *P* = 0.0414). Comparisons between groups were performed using an ANOVA test. Depletion of SUN proteins leads to a small but significant increase in CV, reflecting increased chromosome condensation. (**F**) Quantification of dynein fluorescence intensity on the NE for controls (*n* = 32 cells, 5 replicates) and SUN1/SUN2 shRNA without (*n* = 18 cells, 3 replicates; *P* = 0.0034) or with confinement (*n* = 15 cells, 3 replicates; *P* = 0.0171). Note how confinement does not restore dynein on the NE after SUN1/SUN2 are depleted (*P *= 0.555). Comparisons between groups were performed using an ANOVA test. (**G**) Quantification of the percentage of prophase cells showing NE or nucleoplasmic localization of CENP-F for controls, ICRF-193- or VPA-treated cells. (**H**) Immunofluorescence analysis of NudE/EL localization on the NE after treatment with ICRF-193. In control prophase cells, NudE/EL localize to the NE (yellow arrowhead). This localization is lost after treatment with ICRF-193 (right panel). (**I**) Quantification of NudE/EL fluorescence intensity on the NE after treatment with ICRF-193 (*n* = 20 cells, 4 replicates; *P* = 0.0416, using an unpaired *t* test), in comparison with control cells (*n* = 16 cells, 3 replicates). (**J**) Quantification of the coefficient of variation (CV) for control cells (DMSO, *n* = 32 cells, 3 replicates) and cells treated with ICRF-193 (*n* = 38 cells, 3 replicates, *P* = 0.0435) or VPA (*n* = 35 cells, 3 replicates, *P* = 0.0269). Comparisons between groups were performed using an ANOVA test. (**K**) Quantification of the percentage of prophase cells showing NE or nucleoplasmic localization of CENP-F in control cells, SUN1-depleted or SUN2-depleted cells. (**L**) Representative western blotting analysis of BicD2 levels in control cells and BicD2-depleted cells. (**M**) Representative western blotting analysis of NudE/EL levels in control cells and NudE/EL-depleted cells. On the dot plots, each dot corresponds to an individual value, the line corresponds to the median and the bars correspond to the interquartile range. Square dots represent the median of each replicate in the experiment.

To further dissect the mechanistic link between chromosome condensation, NE tension and mitotic entry, we systematically evaluated how different mechanosensitive NE components known to associate with chromatin, contribute to dynein loading and cyclin B1 translocation (Fig. 6A). Firstly, we disrupted the nuclear lamina in HeLa cells by depleting Lamin A using RNAi (Fig. EV4A). Decreasing Lamin A levels significantly changed the shape of prophase nuclei (Fig. 6B) which became irregular and deformed (Figs. 6C and EV4B), as determined by the nuclear irregularity index (NII). Moreover, these cells showed an increase in membrane displacements across the entire NE (Fig. EV4C,D). Importantly, depleting Lamin A had no effect on dynein loading on the NE (Fig. 6D,E). These data suggest that the nuclear lamina, while necessary for maintaining nuclear shape, is unlikely to be involved in the mechanical remodelling of the nucleus required for mitotic entry. Next, we asked whether this mechanical remodelling involves the LINC complex. This complex is comprised of KASH domain proteins such as Nesprins, that localize to the outer nuclear membrane (ONM) and bind to the cytoskeleton, and SUN proteins on the inner nuclear membrane (INM) that associate with chromatin, creating a physical link between the cytoskeleton and chromatin (Lombardi et al, 2011). We started by expressing a mutant form of KASH tagged with mRFP (DN-KASH) that binds to SUN proteins on the INM and displaces endogenous Nesprins from the ONM. This disrupts the interactions of the LINC complex with the cytoskeleton and blocks force transmission across the NE (Lombardi et al, 2011). As a control, we expressed a version of KASH that cannot interact with SUN proteins (KASH-ΔL) and therefore, does not interfere with endogenous Nesprin localization. As expected, expression of DN-KASH was sufficient to displace endogenous Nesprin-2 from the NE, which did not occur when KASH-ΔL was expressed (Fig. EV4E). Importantly, Nesprin displacement significantly decreased cPLA2 both in the nucleus as well as in the NE (Fig. EV4F,G). This indicates that an intact LINC complex is necessary for the increase in NE tension that occurs during prophase (Fig. 1J,K). These results are also in line with our previous observations in prophase cells showing that the LINC complex-mediated NE tension is required for timely translocation of cyclin B1 across the NPCs (Dantas et al, 2022). However, expression of DN-KASH did not affect the loading of dynein on the NE (Fig. 6F,G). Taken together, these results indicate that, while Nesprin localization on the ONM is required for efficient generation of tension on the NE and cyclin B1 translocation, it is not involved in the regulation of dynein loading. Finally, we asked whether SUN proteins on the INM were required for the mechanical remodelling of the nucleus during mitotic entry. Similar to cells expressing DN-KASH (Dantas et al, 2022), depletion of SUN2 resulted in a significant delay in cyclin B1 nuclear translocation (Fig. 6H,I). The delay observed upon SUN2 depletion is not as striking as when chromosome condensation is disrupted (Fig. 2), suggesting that additional players might be involved. Next, we evaluated whether SUN proteins were required for dynein accumulation on the NE. Depletion of either SUN1 or SUN2 resulted in a significant decrease in the levels of the respective protein (Fig. EV5A–C). This was accompanied by a decrease in dynein accumulation on the NE (Figs. 6J–L and EV5D), which correlated with decreased nuclear levels of cPLA2 (Fig. EV4H,I). Importantly, this loss of dynein in SUN-depleted cells occurred while chromosome condensation was even slightly increased when compared to controls, as determined by the CV measurements (Fig. EV5E). Overall, these data indicate that SUN proteins transmit the signal from mitotic chromosomes to the NE, ensuring timely cyclin B1 translocation and dynein loading, required for mitotic entry. Finally, we wanted to determine if increasing NE tension in cells depleted of SUN1 or SUN2 was sufficient to restore the timing of mitotic entry. Strikingly, confinement of SUN1 or SUN2 depleted cells was unable to rescue dynein loading on the NE (Fig. 6J,K). Similar results were obtained when both proteins were depleted simultaneously using a shRNA approach (Fig. EV5F), indicating that SUN1 and SUN2 likely play redundant roles in this process. Moreover, hypotonic treatment of SUN2-depleted cells also failed to rescue dynein loading on the NE. (Fig. 6L,M). Therefore, we concluded that SUN proteins are essential for transmitting a mechanical signal from condensed mitotic chromosomes to the NE, to enable dynein localization and timely cyclin B1 translocation.

### A chromosome-SUN-NPC axis regulates mitotic entry

**Figure 7:**
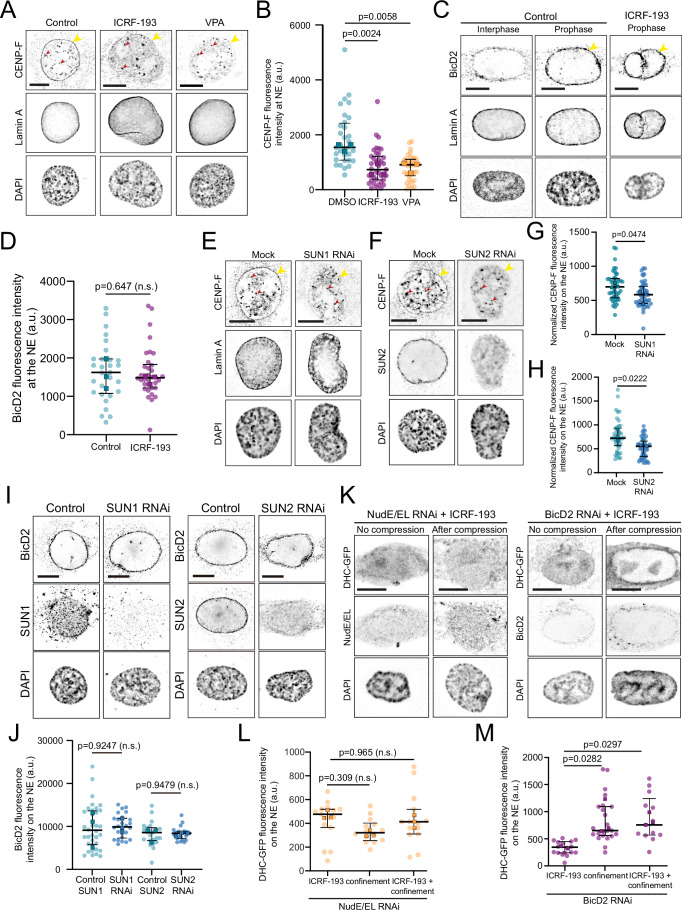
NE tension drives dynein accumulation through the Nup133-CENP-F/NudE/EL pathway. (**A**) Representative immunofluorescence analysis of CENP-F localization in control cells and cells treated with ICRF-193 or VPA. Yellow arrowheads indicate the NE; red arrowheads indicate kinetochores. (**B**) Quantification of the levels of CENP-F on the NE for control cells (*n* = 32 cells, 3 replicates), ICRF-193 (*n* = 39 cells, 3 replicates, *P* = 0.0024) or VPA (*n* = 36 cells, 3 replicates, *P* = 0.0058). Comparisons between groups were performed using an ANOVA test. (**C**) Representative immunofluorescence images of BicD2 localization in control cells (DMSO) or cells treated with ICRF-193. Note the accumulation of BicD2 on the NE of prophase cells (yellow arrowheads), irrespective of the treatment. (**D**) Quantification of fluorescence intensity of BicD2 on the NE of control cells (*n* = 29 cells, 3 replicates) or cells treated with ICRF-193 (*n* = 34 cells, 3 replicates, *P* = 0.647, using a Mann–Whitney test) during prophase. Representative immunofluorescence images of CENP-F localization in cells depleted of SUN1 (**E**) and SUN2 (**F**). Quantification of CENP-F fluorescence intensity on the NE of cells treated with SUN1 RNAi ((**G**); *n* = 45 cells, 3 replicates for control cells and *n* = 48 cells, 3 replicates for SUN1 RNAi, *P* = 0.0474, using an unpaired *t* test) or SUN2 RNAi cells ((**H**); *n* = 43 cells, 3 replicates for control cells and *n* = 49 cells, 3 replicates for SUN2 RNAi, *P* = 0.0222, using an unpaired *t* test), compared to their respective controls. (**I**) Representative immunofluorescence images of BicD2 localization in control cells, as well as cells depleted of SUN1 (left panel) or SUN2 (right panel). (**J**) Quantification of BicD2 fluorescence intensity on the NE following SUN1 (*n* = 36 cells, 3 replicates for controls and *n* = 26 cells, 3 replicates for SUN1 RNAi, *P *= 0.9427, using an unpaired *t* test) or SUN2 (*n *= 23 cells, 3 replicates for controls and n = 23 cells, 3 replicates for SUN2 RNAi, *P* = 0.9479, using an unpaired t-test) depletion. (**K**) Representative immunofluorescence images of dynein accumulation on the NE of prophase cells that were treated with ICRF-193 and depleted of NudE/EL (left panel) or depleted of BicD2 (right panel), without compression or after compression. (**L**) Quantification of dynein fluorescence intensity on the NE following NudE/EL RNAi with either ICRF-193 treatment only (*n* = 15 cells, 3 replicates), compression only (*n* = 14 cells, 3 replicates, *P* = 0.308) or ICRF-193 treatment with compression combined (*n* = 15 cells, 3 replicates, *P* = 0.965). Comparisons between groups were performed using an ANOVA test. (**M**) Quantification of dynein fluorescence intensity on the NE following BicD2 RNAi with either ICRF-193 treatment only (*n* = 16 cells, 3 replicates), compression only (*n* = 23 cells, 3 replicates, *P* = 0.0282) or ICRF-193 treatment with compression combined (*n* = 13 cells, 3 replicates, *P* = 0.0297). Comparisons between groups were performed using an ANOVA test. On the dot plots, each dot corresponds to an individual value, the line corresponds to the median and the bars correspond to the interquartile range. Square dots represent the median of each replicate in the experiment. Scale bars, 10 μm.

Based on our results showing that chromosome condensation affects the morphodynamics and tensional state of the NE in prophase, together with our previous work showing that NE tension promotes cyclin B1 nuclear translocation to control mitotic entry (Dantas et al, 2022), we now propose the following integrative working model: chromosome condensation drives an increase in nuclear volume and NE tension at prophase onset, leading to opening of NPCs. In turn, this triggers a faster nuclear import of cyclin B1 and subsequent nuclear export of CENP-F to the ONM. Together, these translocations would allow the coordination of mitotic entry with chromosome condensation. To test this working model, we designed specific experiments that challenge some of its main predictions. We started by determining whether the loading of dynein on the NE, driven by mitotic chromosome condensation, was dependent on the nuclear export (Loftus et al, 2017) and subsequent accumulation of CENP-F on NPCs (Bolhy et al, 2011). Indeed, treatment with ICRF-193 or VPA significantly reduced the levels of CENP-F specifically on the NE (Figs. 7A,B, yellow arrowheads and  EV5G), while kinetochore binding was not affected (Fig. 7A, red arrowheads). A similar reduction was observed when we analysed the NE levels of NudE/EL, another component of the same dynein loading pathway (Fig. EV5H,I). Inversely, the levels of BicD2, involved in the alternative Nup358-BicD2 dynein loading pathway (Splinter et al, 2010) that does not require nuclear export, were not affected by ICRF-193 treatment (Fig. 7C,D, yellow arrowheads), despite a significant disruption of chromosome condensation (Fig. EV5J). Overall, these data suggest that chromosome condensation, which drives NPC dilation (Fig. 3H,I) and faster translocation of cyclin B1 into the nucleus (Fig. 2C–G), also facilitates the nuclear export and binding of CENP-F to NPCs to allow timely dynein binding.

To further test our model, we then disrupted NE tension in prophase cells and assessed whether this affected the localization of CENP-F on the NE. For this, we depleted SUN1 or SUN2, which are essential for transmitting the chromosome-mediated signal to the NE (Fig. 5J,K) and for ensuring NE tension during prophase (Fig. EV4H,I). Notably, depleting either SUN1 or SUN2 resulted in a significant proportion of cells showing nucleoplasmic localization of CENP-F (Fig. EV5K), together with a reduction in the levels of CENP-F on the NE (Fig. 7E–H). On the other hand, no changes were observed in the levels of BicD2 on the NE when compared to controls (Fig. 7I,J). These data indicate that the increase in NE tension during prophase is required for the nuclear export of CENP-F, to allow timely loading of dynein on the NE. To further confirm these observations, we mechanically stimulated nuclei of cells that were treated with ICRF-193 and depleted of NudE/EL, an essential interactor of CENP-F (Fig. EV5L). Notably, cells with disrupted chromosome condensation that were depleted of NudE/EL, could not localize dynein to the NE even after mechanical stimulation, making them indistinguishable from cells treated with ICRF-193 only (Fig. 7K,L). Inversely, mechanical stimulation of cells treated with ICRF-193 and depleted of BicD2 (Fig. EV5M) was sufficient to rescue NE dynein loading (Fig. 7K,M). Taken together, these data indicate that the chromosome-mediated signal relayed by SUN proteins to the NE facilitates CENP-F export from the nucleus to ensure timely dynein loading and mitotic entry.

## Discussion

Mitotic entry is characterized by an extensive reorganization of cytoplasmic and nuclear structures (Champion et al, 2017; Gavet and Pines, 2010b), necessary for robust spindle assembly and chromosome segregation (Dantas et al, 2022; Lancaster et al, 2013). How these nuclear and cytoplasmic reorganizations are spatially and temporally coordinated to ensure an efficient mitosis remained unclear. Here we identify mitotic chromosome condensation as one of the regulators of mitotic entry. By modulating NE tension, chromosome condensation contributes to the nuclear translocation of cyclin B1 and dynein association with NPCs, thereby determining irreversible mitotic commitment and ensuring an error-free mitosis (Dantas et al, 2022; Furuno et al, 1999).

In higher eukaryotes, mitotic entry is regulated by two mitotic cyclins, cyclin A and cyclin B. During the G2 phase, cyclin A localizes to the nucleus (Pines and Hunter, 1991) and controls the timing of mitosis (Furuno et al, 1999) by triggering the feedback loops required for initial CDK1 activation (De Boer et al, 2008; Hegarat et al, 2020). On the other hand, during the G2 phase, cyclin B1 is mostly cytoplasmic (Pines and Hunter, 1991). As cells prepare to enter mitosis, cyclin B1 translocates into the nucleus where it is maintained until NEP (Gavet and Pines, 2010a). Nuclear translocation of cyclin B1 depends on multiple factors. Its initial accumulation depends on cyclin A (Gong et al, 2007) and is further stimulated by a spatial positive feedback loop (Santos et al, 2012) that depends on NE tension (Dantas et al, 2022) and possibly involves changes in the nuclear import machinery (Gavet and Pines, 2010a). Once in the nucleus, the cyclin B1-CDK1 complex then further promotes chromosome condensation (Abe et al, 2011), NE dynein loading (Baffet et al, 2015), NPC disintegration (Laurell et al, 2011) and nuclear lamina disassembly (Heald and McKeon, 1990). Here, we propose that mitotic chromosome condensation generates an increase in NE tension (Fig. 1) that synergizes with the biochemical pathways regulating the G2-M transition to accelerate the nuclear accumulation of cyclin B1 (Fig. 2), subsequently triggering the downstream events referred above. This model requires that the process of chromosome condensation must start before the accumulation of cyclin B1 in the nucleus. One possibility is that activity of cyclin A in the nucleus could trigger the condensation process (Gong and Ferrell, 2010), leading to an increase in NE tension that would stimulate the translocation of cyclin B1 (Lindqvist, 2010; Santos et al, 2012), resulting in further chromosome condensation (Abe et al, 2011). This mechanism implies that, during the G2-M transition, the NE acts as a mechanosensor, detecting forces produced by chromosomes and relaying this signal to the cell cycle machinery. In fact, our results also demonstrate that disruption of chromosome condensation increases the levels of Wee1, along with a corresponding Wee1-dependent inhibition of CDK1 (Fig. 4), necessary to sustain a delay in mitotic entry. This is in agreement with our previous observations showing that disruption of NE tension is sufficient to increase the levels of CDK1 pY15 and delay mitotic onset (Dantas et al, 2022). How chromosome condensation and NE tension crosstalk with the G2-M checkpoint to set the timing of mitotic entry remains to be determined. Nevertheless, under these conditions, it is possible that cells would only enter mitosis once sufficient cyclin B1-CDK1 translocates to the nucleus to overcome the inhibitory effects of Wee1 (Lindqvist et al, 2009), leading to its proteosomal degradation (Watanabe et al, 2004). Therefore, by increasing NE tension and stimulating the translocation of cyclin B1, we tip the balance towards the nuclear accumulation of the cyclin B1-CDK1 complex, leading to mitotic commitment. Curiously, cyclin A2 can also partially translocate from the nucleus to the cytoplasm to activate key mitotic regulators such as Plk1 (Silva Cascales et al, 2021). This cytoplasmic accumulation is independent of CDK activity, raising the interesting possibility that cyclin A2 translocation could also depend on NE tension. In the future, it would be interesting to investigate whether the spatiotemporal dynamics of these key mitotic regulators depends on the NE tensional state.

**Figure 8:**
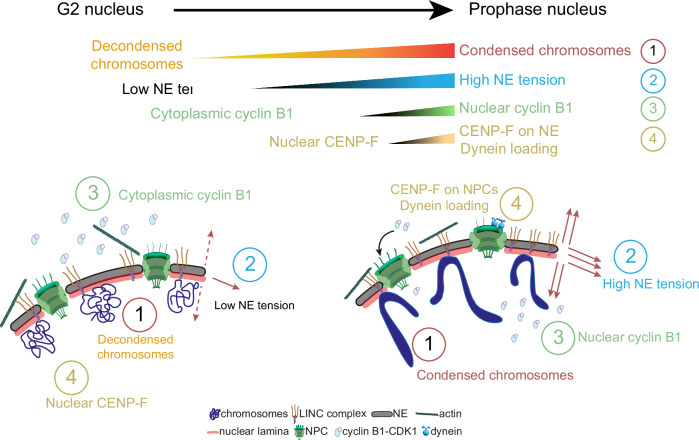
Proposed model for the regulation of mitotic entry by chromosome condensation. During the transition from G2 to mitosis, a series of coordinated events ensure the timing of mitotic onset. The first step involves mitotic chromosome condensation (1), which will lead to an increase in NE tension (2). As tension increases, NPCs dilate and cyclin B1 accumulates in the nucleus (3). Inside the nucleus, active cyclin B1-CDK1 will phosphorylate a series of nuclear targets, including CENP-F (4). This will lead to CENP-F export from the nucleus, enabling dynein loading on NPCs. Together, these allow cells to coordinate mitotic entry with chromosome condensation.

During the G2-M transition, forces exerted on the nucleus change considerably (Lima and Ferreira, 2024). As a result, the NE experiences a significant increase in tension that helps regulate the timing of mitotic entry (Dantas et al, 2022). However, the source of this tension remained unknown. We now propose that during the G2-M transition, the NE senses the tension imposed by condensing chromosomes, eliciting a downstream global cellular response (Fig. 8). On one hand, these forces are transmitted to the cytoplasm through the LINC complex, leading to increased actomyosin contractility (Dantas et al, 2022), NPC dilation (Fig. 1L,M) and contributing to the nuclear accumulation of cyclin B1 (Fig. 2D–G). On the other hand, these chromosome-derived forces also act specifically on the INM through SUN proteins, to enable the nuclear export of CENP-F (Fig. 7A,B) and dynein loading on NPCs. Accordingly, artificially increasing NE tension by confining cells or by hypotonic treatment was sufficient to restore mitotic entry, in cases where chromosome condensation was impaired. Surprisingly, dynein loading on the NE was not restored when confinement was applied to SUN-depleted cells, unlike what happens with expression of DN-KASH (Fig. 6F,G). This led us to hypothesize that SUN-dependent dynein loading relies on the transmission of forces from chromosomes to discreet sites near the NPCs, and not on a global increase of NE tension. This SUN-dependent, localized tension could alter NPC structure in a way that is not recapitulated by our manipulation setup, possibly explaining the lack of rescue under these conditions. In support of this hypothesis, it has been shown that SUN proteins can interact directly with nucleoporins (Jahed et al, 2016; Liu et al, 2007; Talamas and Hetzer, 2011) and concentrate on specific areas of the NE close to centrosomal microtubules (Lima et al, 2024). Moreover, SUN-dependent forces can position NPCs (Smith et al, 2022) and generate tension islands on the NE that are sufficient to induce NPC dilation (Morgan et al, 2025). Nevertheless, the molecular details of this SUN-NPC interaction during the G2-M transition remain to be determined. Intriguingly, our results also indicate the nuclear lamina is not involved in this process. Depleting Lamin A did not affect dynein loading (Fig. 6D), although it significantly changed nuclear shape (Fig. 6B) and increased NE displacements (Fig. EV4D). Overall, these data suggest that the loading of dynein in not dependent on lamin-mediated nuclear mechanics but rather support the hypothesis that it requires the generation of forces in discreet spots at NPCs. It is well known that interactions between chromosomes and the nuclear lamina are important for nuclear structure and mechanics during interphase (Herve et al, 2025; Schreiner et al, 2015; Stephens et al, 2017). However, during the G2-M transition, the nuclear lamina is disassembled in a CDK1-dependent manner to facilitate mitotic entry (Champion et al, 2017; Heald and McKeon, 1990). Together with our data, these observations suggest that chromosome condensation, and not the nuclear lamina, is the main contributor for NE tension during this stage of the cell cycle. Further experiments will be required to determine the contribution of each component of this chromosome-SUN-NPC pathway for nuclear mechanoresponse.

In eukaryotes, cyclins and their associated CDKs orchestrate the transitions between the different phases of the cell cycle (Basu et al, 2022). Deregulation of these transitions often lead to abnormal development (Kostic and Roy, 2002) and are associated with pathological conditions (Pellarin et al, 2025), as well as increased errors in chromosome segregation (Gayek and Ohi, 2016; Seibert et al, 2019). Importantly, recent works including our own, have demonstrated that cell cycle transitions are sensitive to mechanical stimuli (Aureille et al, 2019; Dantas et al, 2022; Donker et al, 2022; Gudipaty et al, 2017; Uroz et al, 2018). In this context, NE tension seems to be particularly relevant, as deformation of this organelle can regulate cell cycle progression by promoting the nuclear localization of key factors (Aureille et al, 2019; Dantas et al, 2022; Elosegui-Artola et al, 2017). Our results now indicate that timely transition into mitosis is sensitive to the tension state of the NE, which is regulated by chromosomes within the nucleus. Such a process would ensure that cells only commit to divide once chromosomes are sufficiently condensed, thus preventing the generation of errors caused by premature mitotic entry (Dantas et al, 2022; Furuno et al, 1999). We propose that mitotic entry is determined by the integration of biochemical and mechanical cues on the NE that enable a more robust coordination between the cytoplasmic and nuclear events, required for efficient cell division.

## Methods

**Table:** Reagents and tools table

Reagent/resource	Reference or source	Identifier or catalog number
**Experimental models**		
HeLa parental (*H. sapiens*)	ATCC	CCL-2
HeLa cyclin B1-Venus (*H. sapiens*)	Pines Lab	
HeLa cyclin B1-Venus/H2B-mRFP (*H. sapiens*)	This study	
HeLa DHC-GFP (*H. sapiens*)	www.mitocheck.org	
HeLa POM121-3xGFP (*H. sapiens*)	Uhlmann Lab	
HeLa POM121-3xGFP/H2B-mRFP (*H. sapiens*)	This study	
RPE-1 cyclin B1-Venus (*H. sapiens*)	Pines Lab Collin et al, 2013	
RPE-1 cyclin B1-eYFP (*H. sapiens*)	Lindqvist Lab Akopyan et al, 2014	
**Recombinant DNA**		
mRFP1-KASH-DN	Gundersen Lab Gant Luxton et al, 2010	
mRFP1-KASH-DL	Gundersen Lab Gant Luxton et al, 2010	
**Antibodies**		
Mouse anti-Lamin A/C	Abcam	4C11
Rabbit anti-Lamin B1	Abcam	ab16048
Rat anti-alpha tubulin	Bio-Rad	MCA77G
Rabbit anti-NudE/NudEL antibody	Vallee Lab (Stehman et al, 2007)	
Rabbit anti-BicD2	Atlas Antibodies	HPA024452
Rabbit anti-SUN1	Merck	HPA008346
Rabbit anti-SUN2	Merck	HPA001209
Rabbit anti-SMC2 antibody	Bethyl Laboratories	A300-056A
Rabbit anti-cPLA2	Cell Signaling	#2832
Rabbit anti-CENP-F	Abcam	ab5
Mouse monoclonal anti-p21WAF1 (Ab-1)	Merck	EA10
Rabbit monoclonal anti-p38 (D13E1)	Cell Signaling Technology	8690
Rabbit monoclonal anti-Wee1 (D10D2)	Cell Signaling Technology	13084
Rabbit anti-CDK1 Y15	Cell Signaling Technology	9111
Mouse anti-Nde1	Abnova	H00054820-M01
Rabbit anti-vinculin	ThermoFisher	700062
Rabbit monoclonal anti-acetyl-Histone H3 (Lys9) (C5B11)	Cell Signaling Technology	9649
Mouse monoclonal anti-Nesprin-2 (SYNE-2, F11)	Santa Cruz Biotechnology	sc-398616
Goat anti-rabbit Alexa Fluor 488	ThermoFisher	A-11008
Goat anti-mouse Alexa Fluor 488	ThermoFisher	A-11001
Goat anti-rabbit Alexa Fluor 568	ThermoFisher	A-11011
Goat anti-mouse Alexa Fluor 568	ThermoFisher	A-11004
Goat anti-rat Alexa Fluor 568	ThermoFisher	A-11077
Goat anti-rat Alexa Fluor 647	ThermoFisher	A-21247
Goat anti-mouse Alexa Fluor 647	ThermoFisher	A-21240
Goat anti-mouse-HRP	Jackson Immuno Research	115-005-003
Goat anti-rabbit-HRP	Jackson Immuno Research	111-005-003
Goat anti-rat-HRP	Jackson Immuno Research	112-005-003
**Oligonucleotides and other sequence-based reagents**		
Lamin A RNAi	Dharmacon ONTARGETplus	LQ-004978-00-0010
BicD2 RNAi	Dharmacon ONTARGETplus	L-014060-01-0020
SUN1 RNAi	Dharmacon ONTARGETplus	L-025277-01-0020
SUN2 RNAi	Dharmacon ONTARGETplus	L-009959-01-0020
NudE siRNA	Sigma-Aldrich	5‘-GCUUGAAUCAGGCCAUCGA-3‘ 5‘-UCGAUGGCCUGAUUCAAGC-3‘
NudEL siRNA	Sigma-Aldrich	5‘-GGAUGAAGCAAGAGAUUUA3‘ 5‘-UAAAUCUCUUGCUUCAUCC-3‘
SMC2 siRNA	Sigma-Aldrich	5′-UGCUAUCACUGGCUUAAAUTT-3
Non-targeting pool siRNA	Dharmacon	#D-001810-10-20
**Chemicals, enzymes and other reagents**		
ICRF-193	Merck-Millipore	I4659
Valproic acid (VPA), sodium salt	Sigma-Aldrich	P4543
MK-1175 (Wee1 inhibitor)	MedChemExpress	HY-10993
RO-3306 (CDK1 inhibitor)	Sigma-Aldrich	SML0569
Thymidine	Sigma-Aldrich	T1895
Dimethyl sulfoxide (DMSO)	Sigma-Aldrich	D4540
Fibronectin (FBN)	Sigma-Aldrich	F1141
Sodium Bicarbonate (NaHCO_3_)		
Dulbecco’s Modified Eagle Medium (DMEM)	ThermoFisher	11965092
Opti-MEM Reduced Serum Medium	ThermoFisher	11058021
DMEM/F12	ThermoFisher	11320033
Fetal Bovine Serum (FBS)	ThermoFisher	A5256801
Lipofectamine™2000 transfection reagent	Invitrogen™	11-668-019
Lipofectamine™ RNAiMAX transfection reagent	Invitrogen™	13778100
Paraformaldehyde (PFA)	Delta Microscopies	D15710
Poly-L-lysine (PLL)	Sigma-Aldrich	P8920
PLL(20)-g[3,5]-PEG(2)	SuSoS	
Fibrinogen From Human Plasma, Alexa Fluor546 Conjugate	ThermoFisher	F13192
Fibrinogen From Human Plasma, Alexa Fluor647 Conjugate	ThermoFisher	F35200
Polydimethylsiloxane (PDMS)	GE	RTV615
Antibiotic Antimycotic solution (AAs)	ThermoFisher	15240062
Leibowitz’s L-15 medium	ThermoFisher	21083027
SiR-DNA	Spirochrome	SC007
Triton™ X-100	Sigma-Aldrich	X100
DAPI (2-(4-amidinophenyl)-1H -indole-6-carboxamidine)	Sigma-Aldrich	D9542
cOmplete™ Protease Inhibitor Cocktail	Merck	04693116001
Bradford Protein Assay	Bio-Rad	5000201
Clarity Western ECL Substrates	Bio-Rad	1705061
NZYColour Protein Marker II	NYZtech	MB09002
HistoGel™	Fisher Scientific	22110678
Tween® 20	Sigma-Aldrich	P1379
40% acrylamide/Bis solution 29:1	Bio-Rad	1610146
**Software**		
Fiji image processing software	ImageJ distribution, NIH	
Trackosome	https://github.com/Trackosome/Trackosome Castro et al, 2020	
MATLAB 2025a	Mathworks	
Zen 3 Microscopy software	ZEISS	
NIS Elements AR	Nikon	
GraphPad Prism 9.5.1	GraphPad Software	
**Other**		
Static cell confiner	4DCell	CSOW 620
Dual vacuum/pressure pump	Elveflow	AF1-Dual
Dynamic cell confiner	Adapted from (LeBerre et al, 2014)	
JEM 1400 transmission electron microscope	JEOL	
Deep UV light	Novascan Technologies	PSD-UV
Plasma generator	Diener Electronic	Zepto Plasma System
Synthetic quartz photomask	Delta Mask	
RMC Ultramicrotome	PowerTome	
iBlot Gel Transfer Device	Bio-Rad	

### Cell lines

Cell lines were cultured in Dulbecco’s Modified Eagle Medium (DMEM; Life Technologies) supplemented with 10% fetal bovine serum (FBS; Life Technologies) and grown in a 37 °C humidified incubator with 5% CO_2_. HeLa cell line expressing histone H2B-GFP/mRFP-α-tubulin was generated in our laboratory using lentiviral vectors, as previously described (Nunes et al, 2020). HeLa DHC-GFP line was a kind gift from Iain Cheeseman. HeLa CyclinB1-Venus cell line expressing endogenously tagged cyclin B1 was a gift from Jonathon Pines. HeLa POM121-3xGFP/H2B-mRFP cell line was kindly made available by Katharine Ullman. RPE-1 cell line expressing endogenously tagged cyclin B1-eYFP was kindly made available by Arne Lindqvist. All cell lines were routinely tested for mycoplasma infection.

### Transient transfection and plasmids

For transient overexpression of mRFP1-KASH-DN or mRFP1-KASH-ΔL (kindly provided by Edgar Gomes), cells were transfected with the corresponding plasmid using Lipofectamine2000 (Invitrogen), according to the manufacturer’s instructions. Briefly, 5 μL of Lipofectamine 2000 and 0.6 μg/mL of DNA of interest were diluted and incubated in Opti-Minimal Essential Medium (Opti-MEM; ThermoFisher) for 30 min. Cells at 50–70% confluence were then incubated with the DNA-lipid complexes for 6 h. Prior to and during transfection, cells were cultured in reduced serum medium (DMEM supplemented with 5% FBS). Transfected cells were analyzed 72 h after transfection.

### RNAi experiments

Cells were transfected with small interfering RNAs (siRNAs) using Lipofectamine RNAi Max (Life Technologies), according to the manufacturer’s instructions. Briefly, 5 μL of Lipofectamine and each siRNA were diluted and incubated in Opti-MEM (ThermoFisher) for 30 min. Cells at 50–70% confluence were then incubated with the siRNA-lipid complexes for 6 h. Prior to and during transfection, cells were cultured in reduced serum medium (DMEM supplemented with 5% FBS). For all siRNAs used, cells were analyzed 48 - 72 h after transfection. Protein depletion efficiency was monitored by immunoblotting or immunohistochemistry. Different concentrations of siRNA were used: 20 nM for Lamin A RNAi, BicD2 and NudE/EL, 40 nM for SUN1 and SUN2 RNAi and 200 nM for SMC2 RNAi. The following commercial ONTARGETplus siRNAs (Dharmacon) were used: Lamin A/C (#LQ-004978-00-0010), BicD2 (#L-014060-00-0020), SUN1 (#L-025277-00-0020), SUN2 (#L-009959-01-0020). For combined NudE/EL depletion the following oligos were ordered from Sigma-Aldrich 5‘-GCUUGAAUCAGGCCAUCGA-3‘ and 5‘-UCGAUGGCCUGAUUCAAGC-3‘ for NudE and 5‘-GGAUGAAGCAAGAGAUUUA3’and 5‘-UAAAUCUCUUGCUUCAUCC-3’ for NudEL. For SMC2 depletion the following oligo was ordered from Sigma-Aldrich 5′-UGCUAUCACUGGCUUAAAUTT-3′. Both commercial ONTARGETplus non-targeting Pool siRNAs (Dharmacon #D-001810-10-20) and mock transfections were used as controls.

### Drug treatments

Inhibition of Topoisomerase II was done using 10 μM of ICRF-193 (Merck-Millipore). Inhibition of histone deacetylases (HDACs) was done using 1.5 μM of VPA (Sigma-Aldrich). Both drugs were added to the culture medium 8–16 h before live-cell imaging or fixation. Control cells were treated with DMSO (Sigma-Aldrich) only.

### Cell synchronization

For the electron microscopy experiments, cells were synchronized using a double-thymidine block. Cells were incubated with 2 mM thymidine (T1895; Sigma-Aldrich) for 16 h, followed by a 10 h release. To release the cells, the cells were washed three times in pre-warmed 1× PBS and incubated in pre-warmed fresh media. A second block was performed for another 16 h, followed by an 8 h release. To release the cells, the cells were washed three times in pre-warmed 1× PBS and incubated in pre-warmed fresh media containing either DMSO (D4540; Sigma-Aldrich) or ICRF-193 (Merck-Millipore).

For imaging experiments, cells were synchronized in late G2 using a CDK1 inhibitor (RO-3306; CDK1i; Sigma-Aldrich SML0569). Cells were incubated with 10 μM RO-3306 for 10–16 h. Before imaging, the inhibitor was washed out three times using pre-warmed fresh medium and incubated in pre-warmed fresh media containing DMSO, ICRF-193 or VPA.

### Electron microscopy

For ultrastructural analysis, cells were previously synchronized using a double thymidine block. Eight hours after the second release from the thymidine block, cells were fixed with 4% paraformaldehyde, 5% glutaraldehyde in 0.2 M sodium cacodylate buffer for 15 min., with agitation. The fixative was removed and a new fixative solution of 2% paraformaldehyde, 2.5% glutaraldehyde, 0.1 M sodium cacodylate was added to the cells for 1 h. The samples were then post-fixed in 2% osmium tetroxide in 0.1 M sodium cacodylate buffer for 2 h and then the cell pellet was resuspended in Histogel^TM^ and stained with a 1% aqueous uranyl acetate solution for 30 min, dehydrated and embedded in Embed-812 resin. Ultra-thin sections (60 nm thick) were sectioned on an RMC Ultramicrotome (PowerTome, USA) using a Diatome diamond knife, picked up on slot grids, and stained with uranyl and lead citrate for 5 min each. The samples were imaged on a JEOL JEM 1400 transmission electron microscope (JEOL, Tokyo, Japan) and the images were digitally recorded using an Orius 1100 W CCD digital camera (Tokyo, Japan). Transmission electron microscopy (TEM) was carried out at the i3S HEMS center, Porto, Portugal. Nuclear pore complex (NPC) size was measured using Fiji (Schindelin et al, 2012).

### Micro-patterning

To control individual cell and nuclear shape, micropatterns were produced as previously described (Azioune et al, 2009; Nunes et al, 2020). Briefly, glass coverslips were activated with plasma (Zepto Plasma System, Diener Electronic) for 1 min and then incubated with 0.1 mg/mL of PLL(20)-g[3,5]-PEG(2) (SuSoS) in 10 mM HEPES at pH 7.4, for 30 min, at room temperature (RT). After rinsing and air-drying, the coverslips were sealed onto a synthetic quartz photomask (Delta Mask), previously activated with deep-UV light (PSD-UV, Novascan Technologies) for 5 min, using 3 μL of MiliQ water. The coverslips were then irradiated through the photomask with the UV lamp for 5 min and incubated with 25 μg/mL of fibronectin (FBN; Sigma-Aldrich) in 100 mM NaHCO_3_ at pH 8.6, for 1 h, at RT. To monitor patterning efficiency and quality, 5 μg/mL of Alexa546 or 647-conjugated fibrinogen (Thermo Fisher Scientific) was added to the FBN solution. Total, 50,000–75,000 cells were seeded per patterned-coverslip and allowed to adhere for 10–15 h before imaging. To wash out non-adherent cells, the cell medium was changed approximately 2–5 h after seeding.

### Cell confinement

For cell confinement experiments, confinement slides were fabricated using glass coverslips covered by a microstructured layer of PDMS, designed with a regular array of micropillars (diameter 449 μm, 1 mm spacing, 8 μm height), as described elsewhere (Le Berre et al, 2014). Briefly, a drop of polydimethylsiloxane (PDMS, RTV615, GE) mixture (8/1 w/w PDMS A/crosslinker B) was poured on top of a microfabricated mold. Glass coverslips with 10 mm diameter, previously activated with plasma for 2 min (Zepto system, Diener Electronics), were placed on top of the PDMS drop and gently pushed with tweezers to obtain a very thin layer of PDMS under the coverslips. The mold containing the coverslips was then baked on the hot plate at 95 °C for 15 min. After removing the excess PDMS, the coverslips were released from the mold using isopropanol and a scalpel blade, rinsed with isopropanol and air dried. Prior to use, confinement coverslips were incubated for at least 1 h with the appropriate culture media and drugs (when necessary). The confinement slides were applied onto cells using either a dynamic or a static cell confiner. To perform dynamic cell confinement experiments (Le Berre et al, 2014), a custom-made suction cup made of a PDMS mixture (10/1 w/w PDMS A/crosslinker B) was baked on the hot plat at 80 °C for 1 h and left to dry overnight. After unmolding the device, a puncher (0.75 mm) was used to create a hole to plug the device into the vacuum generator apparatus (AF1-Dual, Elveflow). The confinement slide with the micropillars was then attached onto the piston of the PDMS suction cup and placed on top of a 35-mm imaging dish device. Cell confinement was modulated by increasing or decreasing the pressure on the vacuum line. For the static cell confinement experiments, we used a commercially available modified 6-well lid (4DCell). In this case, PDMS pillars were attached to the inside of the lid of the 6-well plate and confinement slides were attached to the bottom of the PDMS pillars. Once the lid was placed on the plate and locked, cells became confined. Confinement was removed by unlocking the lid from the plate.

### Time-lapse microscopy

For time-lapse microscopy, cells were seeded on FBN-patterned coverslips one day before imaging. Before each experiment, the cell culture medium was replaced with Leibovitz’s-L15 medium (ThermoFisher Scientific) supplemented with 10% FBS and Antibiotic–Antimycotic solution (AAS; ThermoFisher Scientific). When SiR-dyes (20 nM SiR-tubulin or 10 nM SiR-DNA; Spirochrome) and/or drugs (acute pharmacological inhibition) were used, they were added to the culture medium before acquisition. Live-cell imaging was performed using temperature-controlled Nikon TE2000 microscopes equipped with a modified Yokogawa CSU-X1 spinning-disc head (Yokogawa Electric), an electron multiplying iXon+ DU-897 EM-CCD camera (Andor) and a filter-wheel. The following laser lines were used for excitation: 488, 561 and 647 nm. The experiments were done with an oil-immersion 60×1.4 NA Plan-Apo DIC (Nikon), except for the nuclear pore fluctuation analysis, in which an oil-immersion 100×1.4 NA Plan-Apo DIC (Nikon) was used. Image acquisition was controlled by NIS Elements AR software. Approximately 17–21 z-stacks with a 0.5 μm or 0.7 μm separation were collected every 20 or 30 s. For NE fluctuation analysis, a single z-stack was collected every 200 msec.

### Immunofluorescence

For CENP-F, cPLA2, SUN1, SUN2, Lamin A/C, Lamin B1, BicD2 and NudE/EL immunostainings cells were seeded on FBN-patterned-coverslips and fixed with 4% Paraformaldehyde (PFA) in Cytoskeleton Buffer (274 mM NaCl, 2.2 mM Na2HPO4, 10 mM KCL, 0.8 mM KH2PO4, 4 mM EDTA, 4 mM MgCl2, 10 mM PIPES, 10 nM Glucose, pH 6.1) for 10 min. Subsequently, cells were permeabilized with 0.5% Triton X-100 (Sigma- Aldrich) in 1× phosphate-buffered saline (PBS) for 5 min, and washed with 0.1% Triton X-100 in 1× PBS, 2× for 5 min. The cells were then blocked with 10% FBS in 0.1% Triton X-100 in 1× PBS for 30 min. After blocking, the cells were incubated with the primary antibodies diluted in blocking solution, for 1 h at RT. Next, the cells were washed with 10% Triton X-100 in 1× PBS, 2× for 5 min and subsequently incubated with the respective secondary antibody and DAPI (1 μg/mL, Sigma-Aldrich), diluted in blocking solution, for 45 min at RT. Lastly, cells were washed with 0.1% Triton X-100 in 1× PBS, 2× for 5 min, and sealed on glass slides mounted with 20 mM Tris pH 8, 0.5 N-propyl gallate and 90% glycerol. For SMC2 immunostainings cells were seeded on Poly-L-lysine-(PLL)-coated coverslips (50 μg/mL; Sigma, P8920). Cells were washed with 1× PBS for 1 min, pre-extracted with 0.5% Triton-X100 for 30 s and fixed with 4% PFA in Cytoskeleton Buffer (274 mM NaCl, 2.2 mM Na_2_HPO_4_, 10 mM KCL, 0.8 mM KH_2_PO_4_, 4 mM EDTA, 4 mM MgCl_2_, 10 mM PIPES, 10 nM Glucose, pH 6.1) for 10 min. Next, cells were permeabilized with 0.5% Triton X-100 for 3 min, washed with 0.1% Triton-X100 for 2x for 3 min and blocked with 10% FBS in 0.1% Triton X-100 in PBS for 30 min. After blocking, the cells were incubated with the primary antibodies followed by the secondary antibodies and sealed on glass coverslips as described for the initial immunostainings. The following primary antibodies were used: mouse anti-Lamin A/C (1:500, Abcam), rabbit anti-Lamin B1 (1:500, Abcam), rat anti-alpha tubulin (1:500, Bio-Rad), rabbit anti-NudE/EL antibody (1:500, gift from Richard Vallee), rabbit anti-BicD2 (1:500, Atlas Antibodies) rabbit anti-SUN1 (1:1000, Merck), rabbit anti-SUN2 (1:1000, Merck), rabbit anti-SMC2 antibody (1:500; Bethyl Laboratories) rabbit anti-cPLA2 (1:100, #2832; Cell Signaling), rabbit anti-CENP-F (ab5, 1:300; Abcam). Alexa Fluor 488, 568 and 647 (1:2000, ThermoFisher Scientific) were used as secondary antibodies. Images were acquired using an AxioImager Z1 (×63, plan oil differential interference contract objective lens, 1.4 NA; all from Carl Zeiss) which is coupled with a CCD camera (ORCA-R2; Hamamatsu Photonics). Image acquisition was controlled by Zen software (Carl Zeiss).

### Quantification of fluorescence intensity on the nuclear envelope

Quantification of fluorescence intensity of NE proteins was performed using ImageJ, as described elsewhere (Lima et al, 2024). Briefly, a sum projection of three z-slices encompassing the central region of the nucleus was generated. On the sum-projected image, a segmented line (smoothened by a spline fit) of a defined width (w1) was drawn along the NE, and the transverse-averaged fluorescence signal (S1), containing the fluorescence signal as well as background, was measured. A second equivalent measurement (S2) was done using the same line, after increasing its width to w2. While the signal of interest remains the same in the dilated line, background increases by the factor w2/w1, which allows retrieval of I(r), the background-corrected profile, using the equation:

$$I\left(r\right)=\frac{{w}_{1}{w}_{2}}{{w}_{2}-{w}_{1}}\left({S}_{2}\left(r\right)-{S}_{1}\left(r\right)\right)$$

Line width w1 should be large enough to fully encompass the signal of interest, while w2 should be at least 20% larger than w1, while small enough to avoid inclusion of extraneous signal from non-NE sources. For our quantifications, the intensity profile (i.e. the r-dependence) was irrelevant, so I(r) was integrated along the full length of the curve and divided by the line length (or, equivalently, the line ‘area’).

### Cyclin B1 quantification

For quantifications of cyclin B1 levels and translocation rates, images were analysed using ImageJ. A small circular region of interest (ROI) was defined, and cyclin B1 fluorescence intensity measured, throughout time in the cell nucleus. The same ROI was used to measure the background outside the cell area. All fluorescence intensity values were then background corrected and area normalized. The values obtained were then fitted with the sigmoidal function $y=a+\frac{(b-a)}{1+{e}^{(x-x0)/{dx}}}$ and scaled to their fitted maximum and minimum values. From this, we obtained cyclin B1 translocation rates and half-time. Time zero is defined as the moment when the CDK1 inhibitor is washed out.

### Quantification of nuclear volume, nuclear envelope excess of perimeter (EOP) and coefficient of variation (CV) of DNA

The excess of perimeter (EOP) parameter was calculated to estimate the amount of nuclear envelope (NE) area stored in folds, as described previously (Lomakin et al, 2020). To calculate the EOP, we measured the perimeter (P) and surface area (A) from 2D immunofluorescence images taken at the maximum radius of the nucleus, using Lamin A/C labelling. Next, we defined R0 as the radius of the circle defined by the area A. EOP was obtained as the ratio between (P − 2πR0) and (2πR0). Highly folded nuclei have EOP values close to 1, whereas in nuclei with smooth surfaces, EOP tends to zero.

The nuclear volume was estimated using ImageJ. Briefly, after image thresholding (Otsu method), the area of each slide in the stack was measured individually. The area measurements were then added together and multiplied by the depth of each slice. Finally, the nuclear volume measurements were normalized to the smallest and largest value in the data set using GraphPad.

Chromosome condensation was assessed using the coefficient of variation (CV). This method evaluates the degree of heterogeneity of the DNA signal in the nucleus using DAPI staining (Neguembor et al, 2021), providing a simple way to quantify changes in overall chromatin compaction.

### Nuclear envelope dynamics

Nuclear envelope movements were quantified using the “Membrane fluctuations” package in Trackosome (Castro et al, 2020). Movements are defined as the distance from each point of the median membrane to the membrane at a given frame, along a direction normal to the median membrane. The membranes are segmented and centered for all frames, assuring a common centroid among frames. To obtain a reference membrane of the NE, we calculate the median projection of the centered frames and segment the resulting membrane. Each point of the reference membrane is then associated with a normal vector defining the direction of the membrane displacements. The movements are measured by calculating the distance between the reference membrane and the membrane at each frame, along the directions defined by the normal vectors.

### Fluorescence lifetime imaging (FLIM) of chromosome condensation

FLIM measurements to determine the degree of chromosome condensation are based on an approach described elsewhere (Auduge et al, 2019), with a different instrumentation as follows. Briefly, cells were imaged on a Zeiss LSM 980 (Zeiss, München, Germany) in combination with the LSM Upgrade Kit provided by PicoQuant (PicoQuant, Berlin, Germany). The LSM Upgrade Kit consists of a pulsed laser source at 480 nm generating nanosecond pulses at a repetition rate of 40MH injected into the LSM 980 and a PMA cooled hybrid photomultipliers plugged on a MultiHarp 150 Multichannel time correlated single photon counter which collects the fluorescence signal emitted from the sample through a spectral filter (BrightLine HC 520/35, Semrock). Images were analysed on SymPhoTime software (PicoQuant, Berlin, Germany). Fluorescence decay from chromatin-containing regions of interest is fitted by a monoexponential model, taking into account the instrumental response, yielding the fluorescence lifetime of the EGFP probe.

### Western blotting

HeLa cell extracts were collected, washed, and resuspended in 30-50 μL of Lysis Buffer (20 nM HEPES/KOH pH 7.9; 1 mM EDTA pH 8; 1 mM EGTA; 150 mM NaCl; 0.5% NP40; 10% glycerol, 1:50 protease inhibitor (ROCHE); 1:100 Phenylmethylsulfonyl fluoride). The samples were then flash frozen in liquid nitrogen and kept on ice for 30 min. After centrifugation at 14,000 rpm for 5 min at 4 °C, the supernatant was collected, and protein concentration was determined by the Bradford protein assay (Bio-Rad). 50 μg of protein from each sample were loaded and ran on 15% SDS-PAGE gels. Next, the loaded proteins were transferred to a nitrocellulose Hybond-C membrane using the iBlot Gel Transfer Device (Thermo Scientific). Afterwards the membranes were blocked with 5% Milk in triphosphate-buffered saline (TBS) with 0.1% Tween-20 (TBS-T) for 1 h at RT and subsequently incubated with the primary antibodies overnight at 4 °C with shaking. After three washes in TBS-T, the membranes were incubated with the secondary antibody for 1 h at RT. After several washes with TBS-T, the detection was performed with Clarity Western ECL Substrate (Bio-Rad). The following primary antibodies were used: mouse anti-Lamin A/C (1:500, Abcam), mouse anti-Nde1 (1:500, Abnova), rabbit anti-BicD2 (1:250, Atlas Antibodies) and rat anti-alpha tubulin (1:1000, Bio-Rad). The secondary antibodies used were anti-mouse-HRP, anti-rabbit-HRP and anti-rat-HRP at 1:5000.

### Statistical analysis and data presentation

Three to six independent experiments were used for statistical analysis. No statistical tests were used to estimate sample size and no blinding was done. Knockdown efficiency was assessed by immunofluorescence or immunoblot quantification, as indicated. In all figures, and for visualization purposes only, fluorescence images were deconvolved using ImageJ and maximum intensity projections generated. Quantifications of fluorescence intensity were done using sum-projected images without any further manipulations. Plots show all data points, with the size of the bars (whiskers) ranging from the 25th to the 75th percentile and the line representing the median of the sample. Square dots represent the median of the replicates in each experiment. Statistical analysis for multiple group comparison was performed using a parametric one-way analysis of variance (ANOVA) when the samples had a normal distribution. Otherwise, multiple group comparison was done using a nonparametric ANOVA (Kruskal–Wallis). Multiple comparisons were analyzed using either post-hoc Student-Newman-Keuls (parametric) or Dunn’s (nonparametric) tests. When only two experimental groups were compared, we used either a parametric *t* test or a nonparametric Mann–Whitney test. Distribution normalities were assessed using the Kolmogorov–Smirnov test. No power calculations were used. All statistical analyses were performed using GraphPad Prism 9.5.1 (GraphPad Software).

## Supplementary information

Peer Review File

Movie EV1

Expanded View Figures

## Data Availability

The bioimaging data from this publication have been deposited to the BioImage Archive database (https://www.ebi.ac.uk/bioimage-archive/) and assigned the BioImages accession number S-BIAD3171. The source data of this paper are collected in the following database record: biostudies:S-SCDT-10_1038-S44318-026-00835-8.
