# Supplementary material for: Chromosome condensation mechanically primes the nucleus for mitosis
Source: EMBO J. 2026 Jun 29;45(15):5357–87. doi: 10.1038/s44318-026-00835-8 (PMC13434721; doi:10.1038/s44318-026-00835-8)
Supplement: Supplementary file 1 [file 44318_2026_835_MOESM1_ESM.pdf]

THE EMBO JOURNAL

Chromosome condensation mechanically primes the nucleus for mitosis

Vanessa Nunes, Margarida Moura, Sara Silva, Débora Vareiro, Nicolas Audugé, Nicolas Borghi, and Jorge Ferreira

Corresponding author(s): Jorge Ferreira (jferreir@i3s.up.pt)

Review Timeline:

Submission Date:	23rd Sep 25
Editorial Decision:	28th Nov 25
Revision Received:	2nd Apr 26
Editorial Decision:	21st May 26
Revision Received:	29th May 26
Accepted:	8th Jun 26

Editor: Hartmut Vodermaier

Transaction Report:

(Note: With the exception of the correction of typographical or spelling errors that could be a source of ambiguity, letters and reports are not edited. Depending on transfer agreements, referee reports obtained elsewhere may or may not be included in this compilation. Referee reports are anonymous unless the Referee chooses to sign their reports.)

Dear Dr. Ferreira,

Thank you for submitting your manuscript on chromosomal mechanical control of mitotic entry in human cells. Three experts in spatiotemporal cell cycle regulation (refs 1 & 3) and mechanobiology aspects (ref 2) have now evaluated it, and provided the reports copied below. As you will see, the referees appreciate the potential interest of your findings and model, but raise a number of substantive issues with the conclusiveness of the data and the clarity of their presentation; one of the key concerns being that the link between chromosome condensation and the cell cycle machinery remains to be decisively established.

Since it is not clear whether the various major criticisms could be fully addressed to the referees' satisfaction during a regular round of revision, I would at this point invite you to assemble a tentative point-by-point response and revision proposal, and send this back to me by early next week. Based on such a revision plan, which I may in parts share/discuss with some of the referees, and possible follow-up discussion via video call, we could then get a better understanding of whether a revision for The EMBO Journal would seem realistic, or whether a toned-down version would be a better candidate for rapid publication in one of our sister journals (such as EMBO Reports or Life Science Alliance).

Looking forward to hearing from you,

Best regards,

Hartmut Vodermaier

Hartmut Vodermaier, PhD
Senior Editor, The EMBO Journal
h.vodermaier@embojournal.org

Referee #1 (Report for Author)

In this study the authors have investigated the role of mechanical forces on the timing of entry to mitosis in HeLa cells, and on changes in the nuclear envelope before nuclear envelope breakdown. The authors use mechanical compression, ICRF-193, valproic acid, and various siRNA-depletions to interfere with chromosome condensation or

mechanotransduction, and measure the effect on the timing of mitotic entry after release from an RO3066 arrest, and on the recruitment of dynein and CENP-F to the nuclear envelope. The authors conclude that chromosome condensation triggers mitotic entry by enlarging nuclear pores to increase the nuclear import of Cyclin B1 and export of CENP-F, and mechanosensitive signalling through the LINC complex coordinates nuclear envelope changes to promote NEBD and the reorganisation of the cell.

This is a careful study that reports a series of interesting observations supporting a role for mechanosensitive pathways in reshaping the nuclear envelope at mitotic entry. I have no concerns with this part of the study. Where the study fails to convince is in the link to the regulation of the cell cycle machinery. The major concern here is that the authors never measure the activity of the cell cycle regulators - essential for them to conclude anything about control of the timing of mitotic entry and especially to conclude that Cyclin B-CDK1 is activated first in the nucleus. A second, related, concern, is that the author assume that their perturbations of the nucleus act on the mitotic trigger directly; they do not consider that they may have activated checkpoint pathways that delay mitotic entry. These two concerns mean that the authors' conclusion that chromosome condensation controls mitotic entry is unproven.

More minor concerns are:
1) The study is entirely confined to HeLa cells, which have a perturbed cell cycle due to their aneuploidy and expression of HPV E6 and E7 proteins.
2) The authors measure the timing of mitotic entry by release from a RO3306 arrest. RO3306 inhibits CDK1 and prolonged arrest alters the cell cycle machinery because it inhibits Cyclin A-CDK1 activity as well as preventing Cyclin B-CDK1 activity.
3) The authors assume that the primary actions of ICR-193, valproic acid, and SMC2 depletion are through their effect on chromosome condensation, but this is the only effect they measure and so remains correlation not causation.
4) The authors emphasise the recent paper from the Nurse lab reporting activation of Cyclin B-CDK1 in the nucleus in support of their conclusion that Cyclin B-CDK1 is activated in the nucleus in human cells. The problem here is that cdc13-CDK1 in fission yeast is nuclear, whereas human Cyclin B-CDK1 is cytoplasmic.

Overall, I think they are some interesting data in this paper on the changes in the nuclear envelope at the entry to mitosis but the link to the cell cycle machinery is tenuous.

Referee #2 (Report for Author)

Nunes and colleagues present a study entitled "Chromosome condensation mechanically primes the nucleus for mitosis" where the authors show that that mitotic entry is driven mechanical forces emanating from condensed chromatin and that these forces are required for the coordination between the cytoplasmic and nuclear events required for an efficient mitotic transition. The authors thus add on mechanistic insights with respect to the initial drivers of cyclin B1-CDK1 complex from the cytoplasm to the nucleus to initiate mitotic entry. The authors propose that this signal and thus initial mitotic commitment comes from condensing chromosomes which generate higher tension at the NE to trigger a very complicated downstream cyclin B1/CDK1/SUN proteins/NUPs/Dynein/ CENP-F/NudE/EL pathway.
I am very excited by the findings and the topic but found the paper quite difficult to read and understand -the writing could be streamlined and improved for clarity without contradicting or hinting at interpretations. Importantly, from the abstract onwards, the manuscript is vaguely written with respect to precisely describing the mechanical state of the nuclei that the authors aim to implicate - I would suggest rewriting for precision and clarity statements like "mitotic entry is driven by a nuclear mechanical signal", "spatiotemporally integrated by SUN proteins on the nuclear envelope", "chromosome-generated mechanical signal ", "mitotic onset is controlled by a chromosome-dependent nuclear mechanism", even in figures/results sessions there are vague titles like "Nuclear changes in prophase are determined by chromosome condensation" or "Chromosome condensation generates a mechanical signal on the nucleus". Please define your "mechanical signal" - given that the authors measure NE fluctuation, nuclear pore dilation, and look at cPLA2 they mean to say NE tension from the onset - why not just call it what it is?
Conceptually I do not think that compression is a good model for chromosome-condensation driven increase in NE tension relevant for the biological process at hand - it doesn't really recapitulate the G2-M transition which involves an increase in volume and thus completely different concentration balance scenario when compared to compression which leads to nuclear volume loss. I understand the NE tension but the authors already have a nice paper showing tension is required - to me the novelty here is where the process starts which the author don't state specifically but rather dance around the pool of cyclin B1/CDK1 come from with contradictory discussions.
NPC dilation and transport discussion is very confusing - am I wrong to conclude that the authors believe that the initial cyclin B1/CDK1 event occurs in the nucleus or is it that the NE tension/NE unfolding increases NPC dilation and thus promotes faster transport of cyclin B1/CDK1 to the nucleus? Is there a pool of cyclin B1/CDK in the nucleus? Did the authors show or discuss this? I am not sure this was experimentally shown so it is difficult to understand in the current form what exactly the message is from the authors. You might

consider making a clear point on this as it is an important detail.
What is the link between cPLA2 activation and opening of NPCs - model is proposed but exact link unclear? Or do the authors use it just a measure of NE tension?
In figure 5, siLMNA for figure 5B the chosen nucleus is a raisin when looking at nuclear shape/lamin B then then the chosen nucleus to examine dynein loading in figure 5D is quite normal looking - again we need to see a lot more than one nucleus for all of the quantifications and there is likely a lot of variability
I find the SUN1/2 inhibition data a bit puzzling as it has the same effect under control and confined conditions so it is really tension-independent then this really makes the story again confusing - please reason here. In my view it also appears surprising that SUN proteins would be required for the force transmission given the huge conformational change of the chromatin with condensation. Maybe elaborate a bit, perhaps even graphically how the authors see the temporal evolution of force balancing across the INM/ONM and all the players involved before/during/after mitotic commitment and/or chromosome condensation - describing this 15min process clearly graphically would help.
Can the authors reason why they believe their mechanism is really the NE tension versus nuclear unfolding during prophase? What were the experiments that uncoupled these two?
What authors do show is that chromosome condensation is required for NE unfolding/tension and NPC dilation and that compression induces dynein loading to NE (they have already showed the cyclinB and tension part earlier) but then they go on to discuss chromosome-NE interaction and how this is critical - again without really showing or focusing on this aspect in the work - these discussions distract from the main points. I reason this is where the authors are going?
Is it possible to detect/image condensin activity in the nucleus and time it to cyclinB1/CDK1 activation to address where the initial signal comes from (ie peak of condensin activity first or cyclin B1/CDK1?)
Please define DHC-GFP somewhere before using it.
DHC-GFP that is quantified in 6E is missing a representative image
I would suggest introducing CENP-F and discussion on nuclear export of CENP-F, allowing the loading of dynein on the NE, earlier in the introduction - in general introduce all the players at the onset so that then the logic is clear. I would also suggest modifying your diagram in figure 5 (why is plectin there pictured!?) and bringing it into the intro or similar.
For all figures it is necessary to provide many more than 1 image to be able to access the robustness of the phenotype especially since the authors are analyzing 20-30cells. It appears that all the statistics were done improperly using cell numbers and not n numbers - cannot use cells to calculate significance; please correct
Given the reputation of the authors, the data behind all direct experimental observations and interpretations are exciting and if the authors believe there is a pool of nucleoplasmic

cyclinB1/CDK1 and that the process starts in the nucleus, that is new - I would review cell numbers which are quite low and statistics should be redone and all replicas means shown (no boxplots should be used with all data point hidden but the outliers) with a few exceptions I pointed to above but I must say the manuscript is not written in a way that is helpful to the message - it is not always clear, especially to a non-expert, why the vast majority of experiments were done - there are too many players and too many "axis" of regulation without direct evidence for cylin B1-CDK1 being activated in the nucleus in the first place at the onset, which may or may not be proposed to be the case. I think the story is too complicated - condensing chromatin, SUN proteins, Nesprins, NUPs, cPLA2, cyclin B1-CDK, dynein, BicD2, Nup133/CENP-F/NudE/EL pathway - everything is involved - it is really hard to digest this paper and if the authors feel it is important to include all of them then a rewrite session will be helpful.

Referee #3 (Report for Author)

This is an interesting manuscript that suggests, and provides data in support of, a model in which chromosome condensation drives permeabilization of the nuclear envelope, nuclear translocation of Cyclin B-Cdk1 and commitment to mitotic entry. In general, the experiments are well performed, and the text is well formulated. The results are relevant in particular for the cell cycle community, but also for a wider audience, as it suggests a mechanism for connecting chromosome conformation to biochemical signals.

I have two points that I think should be addressed before publication.

Cdk1 activation gradually increases throughout G2 phase, but in this experimental setup Cdk1 is inhibited through a prolonged G2, which affects transcription through G2 and the balance of kinase activities when Cdk1 inhibition is removed to start mitotic entry. Thus, the starting point of the experiments may not necessarily reflect the situation when mitosis starts during unperturbed proliferation. Key results need to be validated independent of synchronization by Cdk1 inhibition.

The manuscript focuses only on Cyclin B-Cdk1. Cyclin A-Cdk1 (and Cdk2) and its partial cytoplasmic translocation in G2 should at least be discussed. The role of Cyclin A should in particular be mentioned in the discussion of how the spatial feedback is initiated.

Additional suggestion:

In case the connection between chromosome condensation and Cyclin B-Cdk1

translocation is driving mitotic entry, a prediction could be that blocking chromosome condensation would delay mitotic entry even when artificially activating Cdk1. Would inhibition of chromosome condensation delay mitotic entry forced by Wee1 inhibition?

Manuscript: EMBOJ-2025-122546

Point-by-point response to referees/Revision Plan

Response to Referee #1

1 - "Authors never measure the activity of the cell cycle regulators - essential for them to conclude anything about control of the timing of mitotic entry and especially to conclude that Cyclin B-CDK1 is activated first in the nucleus".

We thank the referee for this observation. We agree that we cannot conclude from our data that Cyclin B-CDK1 is first activated in the nucleus. This is a possibility that is mentioned in the Discussion only, based on recently published data (Kapadia and Nurse, Nature, 2025). However, we should point out that this is not an absolute requirement for our model. Activation of the cyclin B1-CDK1 complex can occur in the cytoplasm, but the impairment in nuclear translocation of the complex imposed by disrupting chromosome condensation would still result in a delay in mitotic entry. In support of this hypothesis, it was previously shown that low levels of cyclin B1-CDK1 activity are sufficient to induce its translocation into the nucleus (Gavet and Pines, Dev. Cell, 2010). These could be enough to initiate chromosome condensation (Kimura et al., Science, 1998; Abe et al., Genes Dev., 2011) and trigger the mechanical signal on the nucleus. We propose to clarify these points in the discussion. Additional clarifications involving experimental work are outlined in the next comment.

2 - "…the major concern here is that the authors never measure the activity of the cell cycle regulators - essential for them to conclude anything about control of the timing of mitotic entry".

"A second, related, concern, is that the author assume that their perturbations of the nucleus act on the mitotic trigger directly; they do not consider that they may have activated checkpoint pathways that delay mitotic entry".

These represent valid concerns raised by the referee. To address both points, we propose to synchronize cells using a double thymidine block. Then, after disrupting chromosome condensation, we will perform an immunofluorescence analysis of cell-cycle regulators that control the G2-M transition. We will analyse the levels of Wee1 (Masuda et al., MBoC, 2011), p21 (Hervé et al., Nat Cell Biol, 2025) and p38 (Mikhailov et al., Curr Biol, 2007). Moreover, we will evaluate the levels of phospho-CDK1 Y15 (Heald et al., Cell, 1993; McGowan and Russel, EMBO J, 1993), that represents an inactive form of the kinase. Comparing the levels between controls and treated cells should inform us whether chromosome condensation impacts the cell cycle machinery or activates checkpoint pathways to delay mitotic entry.

3 - "The study is entirely confined to HeLa cells, which have a perturbed cell cycle due to their aneuploidy and expression of HPV E6 and E7 proteins".

This is an accurate observation. However, we would like to point out that by using a synchronization protocol, we are ensuring that cells are starting from a similar stage, which should minimize effects due to cell cycle asynchrony. Under these conditions, our control HeLa cells are significantly different than our treated cells. Overall, we believe these conditions allow us to conclude that our treatments have a specific effect on mitotic entry. Nevertheless, to further strengthen the validity of our observations, we propose to use near-diploid, untransformed RPE-1 cells to perform key experiments that will confirm our observations with HeLa cells. We have previously used RPE-1 cells to show that nuclear translocation of cyclin B1 depends on nuclear tension (Dantas et al., J Cell Biol, 2022). Now we will disrupt chromosome condensation in RPE-1 cells using VPA or ICRF-193 and evaluate the dynamics of cyclin B1 and mitotic entry.

4 - "The authors measure the timing of mitotic entry by release from a RO3306 arrest. RO3306 inhibits CDK1 and prolonged arrest alters the cell cycle machinery because it inhibits Cyclin A-CDK1 activity as well as preventing Cyclin B-CDK1 activity".

Again, a valid point raised by the referee. We would like to point out that our control cells, which are also treated with the CDK1 inhibitor quickly enter mitosis (16.1±14.1 min), while treated cells are significantly delayed (Fig. 2 and Fig. S3 in our manuscript). Nevertheless, to rule out a synergistic deleterious effect of CDK1 inhibition with the other treatments, we will use a double thymidine synchronization protocol (which we have previously implemented in our laboratory) and measure cyclin B1 translocation in either control cells or cells with disrupted chromosome condensation. This will provide a CDK1-independent way to assess the effect of chromosome condensation on mitotic entry.

5 - "The authors assume that the primary actions of ICR-193, valproic acid, and SMC2 depletion are through their effect on chromosome condensation, but this is the only effect they measure and so remains correlation not causation".

We thank the referee for this comment. Our assumption on the action of these treatments on chromosome condensation is based on extensive published literature (e.g., Ishida et al., J Cell Bio, 1994; Biggs et al., MBoC, 2019; Hirota et al., J Cell Sci, 2005). These treatments were selected to target the multiple components involved in the process of condensation. We confirmed their impact on chromosome condensation by measuring the coefficient of variation (CV) parameter. Although we cannot formally rule out any secondary side-effects of these treatments, we believe it unlikely that they would lead to the same side-effects and induce delays in mitotic entry by alternative pathways.

6 - "The authors emphasise the recent paper from the Nurse lab reporting activation of Cyclin B-CDK1 in the nucleus in support of their conclusion that Cyclin B-CDK1 is activated in the nucleus in human cells. The problem here is that cdc13-CDK1 in fission yeast is nuclear, whereas human Cyclin B-CDK1 is cytoplasmic".

This is a relevant point already raised by the referee in a previous comment (please see point 1 above). We would like to emphasize that the nuclear activation of cyclin B1-CDK1 is just one possible hypothesis suggested in the discussion, which was not formally tested. It is known that in G2, cyclin B1 is continuously shuttling between the cytoplasm and the nucleus, with nuclear export rate exceeding nuclear import (Hagting et al., Curr Biol, 1999; Hagting et al., EMBO J, 1998; Yang et al., J Biol Chem, 2001). During early, mitosis, phosphorylation of cyclin B1 in its cytoplasmic retention sequence (CRS) enhances its nuclear translocation (Hagting et al., Curr Biol, 1999). Therefore, it is perfectly possible that cyclin B1-CDK1 is first activated in the cytoplasm. This reduced activity is, nevertheless, sufficient to induce cyclin B1 translocation into the nucleus (Gavet and Pines, Dev. Cell, 2010) and initiate chromosome condensation (Kimura et al., Science, 1998; Abe et al., Genes and Dev, 2011), thereby setting off the positive feedback loop that drives further cyclin B1 translocation. We propose to clarify and contextualize this point in the manuscript.

Response to Referee #2

"I am very excited by the findings and the topic but found the paper quite difficult to read and understand -the writing could be streamlined and improved for clarity without contradicting or hinting at interpretations. Importantly, from the abstract onwards, the manuscript is vaguely written with respect to precisely describing the mechanical state of the nuclei that the authors aim to implicate - I would suggest rewriting for precision and clarity statements like "mitotic entry is driven by a nuclear mechanical signal", "spatiotemporally integrated by SUN proteins on the nuclear envelope", "chromosome-generated mechanical signal ", "mitotic onset is controlled by a chromosome-dependent nuclear mechanism", even in figures/results sessions there are vague titles like "Nuclear changes in prophase are determined by chromosome condensation" or "Chromosome condensation generates a mechanical signal on the nucleus". Please define your "mechanical signal" - given that the authors measure NE fluctuation, nuclear pore dilation, and look at cPLA2 they mean to say NE tension from the onset - why not just call it what it is?"

We thank the referee for this insightful comment and suggestions and apologize for the lack of clarity. We propose to clarify the ambiguous sentences throughout the manuscript by specifically referring to the events analysed.

"Conceptually I do not think that compression is a good model for chromosome-condensation driven increase in NE tension relevant for the biological process at hand - it doesn't really recapitulate the G2-M transition which involves an increase in volume and thus completely different concentration balance scenario when compared to compression which leads to nuclear volume loss".

This is a relevant point raised by the referee, although we observe that compression does rescue the timing of mitotic entry. Nevertheless, to resolve this issue, we propose to use hypotonic treatment that triggers an increase in nuclear volume (e.g. Deviri and Safran, PNAS, 2022) and that we previously showed increases NE tension (Dantas et al., J Cell Biol, 2022). To this end, we will disrupt chromosome condensation as described in our manuscript and impose a hypotonic shock while filming cells during mitotic entry. We anticipate this will trigger cyclin B1 translocation and possibly dynein loading, rescuing the delay imposed by disrupting chromosome condensation.

"I understand the NE tension but the authors already have a nice paper showing tension is required - to me the novelty here is where the process starts which the author don't state specifically but rather dance around the pool of cyclin B1/CDK1 come from with contradictory discussions."

We apologize for the lack of clarity. We propose to rewrite the text to focus on the role of chromosome condensation as the source of the downstream nuclear changes required for mitotic entry.

"NPC dilation and transport discussion is very confusing - am I wrong to conclude that the authors believe that the initial cyclin B1/CDK1 event occurs in the nucleus or is it that the NE tension/NE unfolding increases NPC dilation and thus promotes faster transport of cyclin B1/CDK1 to the nucleus? Is there a pool of cyclin B1/CDK in the nucleus? Did the authors show or discuss this? I am not sure this was experimentally shown so it is difficult to understand in the current form what exactly the message is from the authors. You might consider making a clear point on this as it is an important detail."

Again, we apologize for the lack of clarity. Based on our data, we believe that an increase in tension leads to unfolding of the NE and NPC dilation which promotes faster transport of cyclin B1.

The part of the comment related with the nuclear pool of cyclin B1 goes along the same lines of what was mentioned by Referee #1. We did not directly measure the nuclear localization or activity of cyclin B1-CDK1. However, as we mention in our response to referee #1, it is known that in G2 cyclin B1 is continuously shuttling between the cytoplasm and the nucleus, with nuclear export rate exceeding nuclear import (Hagting et al., Curr Biol, 1999; Hagting et al., EMBO J, 1998; Yang et al., J Biol Chem, 2001). Therefore, while nuclear cyclin B1-CDK1 is residual in comparison with the cytoplasmic pool, it could still be sufficient to trigger chromosome condensation, setting off the positive feedback loop that drives further cyclin B1 translocation. We will rewrite the text to clarify this information and refine the mechanism we propose.

"What is the link between cPLA2 activation and opening of NPCs - model is proposed but exact link unclear? Or do the authors use it just a measure of NE tension?"

Based on previous works, we use cPLA2 recruitment to the NE simply as a proxy for NE tension (Lomakin et al., Science, 2020; Enyedi et al., Cell, 2016; Dantas et al., J Cell Biol, 2022). Also based on a recent report, NE tension was shown to be

sufficient to dilate NPCs (Zimmerli et al., Science, 2021). In our model, we propose that increased NE tension during prophase, which we confirm by the recruitment of cPLA2 to the NE, drives NPC dilation. In agreement with this hypothesis, we observed a decrease in cPLA2 recruitment to the NE when chromosome condensation was disrupted (Fig. 3C-E, 3G).

"In figure 5, siLMNA for figure 5B the chosen nucleus is a raisin when looking at nuclear shape/lamin B then then the chosen nucleus to examine dynein loading in figure 5D is quite normal looking - again we need to see a lot more than one nucleus for all of the quantifications and there is likely a lot of variability".

We thank the referee for the careful analysis. Indeed, there is a significant variability in nuclear shape upon Lamin A depletion, as can be observed by scattering of the values of "nuclear irregularity index" (NII) in figure 5C. To clarify this point, we will add additional examples to the figure, to better reflect the variability in nuclear shapes.

"I find the SUN1/2 inhibition data a bit puzzling as it has the same effect under control and confined conditions so it is really tension-independent then this really makes the story again confusing - please reason here.

Thank you for this comment. Based on the results obtained after SUN1/2 depletion, we propose two independent roles for SUN proteins. Firstly, through the LINC complex, they are involved in the increase in NE tension necessary to trigger nuclear translocation of cyclin B1. Indeed, depletion of SUN1/2 lead to a decrease in cPLA2 levels on the NE (Fig. S4E, F) and a delay in cyclin B1 translocation (new data recently collected, to be included in a revised manuscript). Secondly, we propose that SUN proteins act in a LINC complex-independent way to transmit localized tension generated by chromosomes to the NPCs. This is supported by observations that cells depleted of SUN1/2 are unable to load CENP-F on the NE (Fig. 6E-H) and rescue dynein on NPCs even after confinement (Fig. 5J-L). This LINC complex-independent role does not necessarily require a global increase in NE tension. However, to address the referee´s concern, we propose to use an alternative mode of increasing NE tension, using a hypotonic shock as described above. We will treat cells depleted of SUN1/2 with a hypotonic solution, (which should mimic nuclear volume increase) and evaluate the levels of dynein on the NE. Based on this, we will also clarify and discuss the role of SUN proteins in the text.

"In my view it also appears surprising that SUN proteins would be required for the force transmission given the huge conformational change of the chromatin with condensation. Maybe elaborate a bit, perhaps even graphically how the authors see the temporal evolution of force balancing across the INM/ONM and all the players involved before/during/after mitotic commitment and/or chromosome condensation - describing this 15min process clearly graphically would help".

We thank the referee for this valuable comment. We propose to modify our graphical model to include the temporal order of events.

“Can the authors reason why they believe their mechanism is really the NE tension versus nuclear unfolding during prophase? What were the experiments that uncoupled these two? What authors do show is that chromosome condensation is required for NE unfolding/tension and NPC dilation and that compression induces dynein loading to NE (they have already showed the cyclinB and tension part earlier) but then they go on to discuss chromosome-NE interaction and how this is critical - again without really showing or focusing on this aspect in the work - these discussions distract from the main points. I reason this is where the authors are going?”

Regarding this point, we would like to clarify that we do not intend to separate the contribution of unfolding from tension. We think unfolding of NE membranes during this stage leads to increased tension, given the observed increase in the recruitment of cPLA2. This is similar to what we and others have previously shown (Lomakin et al., Science, 2020; Enyedi et al., Cell, 2016; Dantas et al., J Cell Biol, 2022). Moreover, in a previous publication we also observed that increasing NE folding by LBR overexpression leads to a decrease in NE tension in RPE-1 cells (Dantas et al., J Cell Biol, 2022). We propose to clarify these points in the manuscript and avoid generalizations regarding chromosome-NE interactions.

“Is it possible to detect/image condensin activity in the nucleus and time it to cyclinB1/CDK1 activation to address where the initial signal comes from (ie peak of condensin activity first or cyclin B1/CDK1?)”

It is not possible to directly measure condensin activity in our experimental conditions. However, it is possible to determine its association with chromosomes, which is a prerequisite for condensin-mediated chromosome condensation. To address this point, we will measure the dynamics of condensin II association to chromosomes and correlate this with cyclin B1 nuclear levels. We will focus on condensin II as this protein is nuclear during prophase and is involved in the initial steps of chromosome condensation (Walther et al., J Cell Biol, 2018; Ono et al., MBoC, 2004), unlike condensin I which has cytoplasmic localization until nuclear envelope breakdown.

“Please define DHC-GFP somewhere before using it. DHC-GFP that is quantified in 6E is missing a representative image”

We will update the manuscript to include these changes.

“I would suggest introducing CENP-F and discussion on nuclear export of CENP-F, allowing the loading of dynein on the NE, earlier in the introduction - in general introduce all the players at the onset so that then the logic is clear. I would also suggest modifying your diagram in figure 5 (why is plectin there pictured!?) and bringing it into the intro or similar”.

These changes will be introduced in a revised version of the manuscript.

“For all figures it is necessary to provide many more than 1 image to be able to access the robustness of the phenotype especially since the authors are analyzing 20-30cells.

It appears that all the statistics were done improperly using cell numbers and not n numbers - cannot use cells to calculate significance; please correct"

We apologize for the lack of clarity. We will provide additional images for all figures and where necessary.

Whenever lower cell numbers are presented, these are related to experiments that are technically very challenging, combining live-cell analysis with depletions and confinement. For these reasons, it is very difficult to obtain large cell numbers. Moreover, working with mitosis is particularly challenging, considering that any stress to the cell will inevitably result in cell cycle arrest. For most experiments, the number of cells measured was usually between 30-50 cells per condition. These cell numbers are within what is routinely used in the field. We will clarify all statistical tests and define the number of replicates in each condition. All experiments were independently replicated a minimum of three times.

"Given the reputation of the authors, the data behind all direct experimental observations and interpretations are exciting and if the authors believe there is a pool of nucleoplasmic cyclinB1/CDK1 and that the process starts in the nucleus, that is new - I would review cell numbers which are quite low and statistics should be redone and all replicas means shown (no boxplots should be used with all data point hidden but the outliers) with a few exceptions I pointed to above but I must say the manuscript is not written in a way that is helpful to the message - it is not always clear, especially to a non-expert, why the vast majority of experiments were done - there are too many players and too many "axis" of regulation without direct evidence for cyclin B1-CDK1 being activated in the nucleus in the first place at the onset, which may or may not be proposed to be the case. I think the story is too complicated - condensing chromatin, SUN proteins, Nesprins, NUPs, cPLA2, cyclin B1-CDK, dynein, BicD2, Nup133/CENP-F/NudE/EL pathway - everything is involved - it is really hard to digest this paper and if the authors feel it is important to include all of them then a rewrite session will be helpful."

Again, we apologize for the lack of clarity. We will change the graphs to include all data points and means and update all statistical tests.

Moreover, as we mentioned above, we will rewrite the manuscript to improve clarity and simplify the message.

Response to Referee #3

"Cdk1 activation gradually increases throughout G2 phase, but in this experimental setup Cdk1 is inhibited through a prolonged G2, which affects transcription through G2 and the balance of kinase activities when Cdk1 inhibition is removed to start mitotic entry. Thus, the starting point of the experiments may not necessarily reflect the situation when mitosis starts during unperturbed proliferation. Key results need to be validated independent of synchronization by Cdk1 inhibition".

We thank the referee for this relevant comment, which is shared by referee #1. Using synchronizations is a convenient way to ensure that cells are starting from a similar state. This also provides us with a reference point to start our measurements. To clarify that the delays we see are not due to Cdk1 inhibition, we will assess mitotic entry using a double thymidine synchronization protocol (which we have used previously). We will also quantify the timing of mitotic entry

in unperturbed cells, to confirm that Cdk1 inhibition is not negatively impacting the dynamics of the process. This will also be confirmed by analysing the levels of specific cell cycle markers for G2 phase, as was proposed in our response to an observation made by referee #1.

"The manuscript focuses only on Cyclin B-Cdk1. Cyclin A-Cdk1 (and Cdk2) and its partial cytoplasmic translocation in G2 should at least be discussed. The role of Cyclin A should in particular be mentioned in the discussion of how the spatial feedback is initiated."

We thank the reviewer for pointing out the relevant role of cyclin A-cdk1 complex in mitotic entry. We will address this point in the discussion.

"In case the connection between chromosome condensation and Cyclin B-Cdk1 translocation is driving mitotic entry, a prediction could be that blocking chromosome condensation would delay mitotic entry even when artificially activating Cdk1. Would inhibition of chromosome condensation delay mitotic entry forced by Wee1 inhibition?"

This is a very interesting suggestion by the referee. We should point out that the biochemical pathway mediated by cyclin B1-CDK1 is essential for mitotic entry. Indeed, mechanically forcing the translocation of cyclin B1 into the nucleus of CDK1-inhibited cells does not trigger mitotic entry (Dantas et al., J Cell Biol, 2022). Following the referee`s comment, we propose to determine the timing of mitotic entry in cells with impaired chromosome condensation and treated with a Wee1 inhibitor. These will be compared with unmanipulated cells and with cells that have normal chromosome condensation and Wee1 inhibition.

Prof. Jorge G. Ferreira
Instituto de Investigação e Inovação em Saúde -i3S
Epithelial Polarity and Cell Division
Rua Alfredo Allen
Porto 4200-315
Portugal

28th Nov 2025

Re: EMBOJ-2025-122546
Chromosome condensation mechanically primes the nucleus for mitosis

Dear Dr. Ferreira,

Thank you for your letter outlining how you might address the referee comments on your recent EMBO Journal submission. I have now had a chance to go through your tentative point-by-point rebuttal, and I noticed that your response and revision plan appear very sensible and should in principle be well-suited for addressing the most salient concerns raised by the reviewers. In particular, your proposals to test an alternative synchronization protocol, to look at relevant cell-cycle regulators, and to also use hypotonic treatment appear to be well taken; and I also appreciate the planned validation of key experiments in RPE-1 cells and the added discussions of CDK1-Cyclin B activation location.

I would therefore be happy to invite you to revise the study along the proposed lines, and to resubmit it to The EMBO Journal using the link below. It is our policy to allow only a single round of (major) revision, so please keep me updated should there be any unexpected problems with the revisions, or should you require an extension beyond the default 3-months deadline. As always, competing manuscript published elsewhere during the course of this revision will not affect our eventual decision on your study. Finally, please note the detailed information and guidelines on how to prepare a revision below (and in our online Guide to Authors) - closely adhering to them shall greatly facilitate the editorial process at the time of resubmission.

Thank you again for the opportunity to consider this work, and I look forward to receiving your revision in due time.

With kind regards,

Hartmut

Hartmut Vodermaier, PhD
Senior Editor, The EMBO Journal
h.vodermaier@embojournal.org

*** PLEASE NOTE: All revised manuscript are subject to initial checks for completeness and adherence to our formatting guidelines. Revisions may be returned to the authors and delayed in their editorial re-evaluation if they fail to comply to the following requirements. As a first step please read our guidelines for revised submissions:
https://link.springer.com/journal/44318/submission-guidelines#cms-Revised-submissions

1) Every manuscript requires a Data Availability section (even if only stating that no deposited datasets are included). Primary datasets or computer code produced in the current study have to be deposited in appropriate public repositories prior to resubmission, and reviewer access details provided in case that public access is not yet allowed.

2) Each figure legend must specify
- size of the scale bars that are mandatory for all micrograph panels
- the statistical test used to generate error bars and P-values
- the type error bars (e.g., S.E.M., S.D.)
- the number (n) and nature (biological or technical replicate) of independent experiments underlying each data point
- Figures may not include error bars for experiments with n<3; scatter plots showing individual data points should be used instead.

3) Revised manuscript text (including main tables, and figure legends for main and EV figures) has to be submitted as editable text file (e.g., .docx format). We encourage highlighting of changes (e.g., via text color) for the referees' reference.

4) Each main and each Expanded View (EV) figure should be uploaded as individual production-quality files (preferably in .eps,

.tif, .jpg formats). For suggestions on figure preparation/layout, please refer to our Figure Preparation Guidelines: https://media.springernature.com/original/springer-cms/rest/v1/content/27825798/data/v1

5) Point-by-point response letters should include the original referee comments in full together with your detailed responses to them (and to specific editor requests if applicable), and also be uploaded as editable (e.g., .docx) text files.

6) Please complete our Author Checklist, and make sure that information entered into the checklist is also reflected in the manuscript; the checklist will be available to readers as part of the Review Process File.

7) All authors listed as (co-)corresponding need to deposit, in their respective author profiles in our submission system, a unique ORCiD identifier linked to their name. Please see our Guide to Authors for detailed instructions.

8) Please note that supplementary information at EMBO Press has been superseded by the 'Expanded View' for inclusion of additional figures, tables, movies or datasets; with up to five EV Figures being typeset and directly accessible in the HTML version of the article.

9) To facilitate reproducibilty and cross-laboratory adoption of methodologies, please structure the Materials & Methods section as outlined in our guide to authors, including a completed Reagents and Tools Table.

10) Digital image enhancement is acceptable practice, as long as it accurately represents the original data and conforms to community standards. If a figure has been subjected to significant electronic manipulation, this must be clearly noted in the figure legend and/or the 'Materials and Methods' section. The editors reserve the right to request original versions of figures and the original images that were used to assemble the figure. Finally, we generally encourage uploading of numerical as well as gel/blot image source data.

At EMBO Press we ask authors to provide source data for the main manuscript figures. You will receive a separate email with instructions for providing source data with your revised manuscript, including how to upload and organize the files.

In the interest of ensuring the conceptual advance provided by the work, we recommend submitting a revision within 3 months (26th Feb 2026). Please discuss the revision progress ahead of this time with the editor if you require more time to complete the revisions. Use the link below to submit your revision:

Link Not Available

Manuscript: EMBOJ-2025-122546

Point-by-point response to referees

Response to Referee #1

1 - "Authors never measure the activity of the cell cycle regulators - essential for them to conclude anything about control of the timing of mitotic entry and especially to conclude that Cyclin B-CDK1 is activated first in the nucleus".

We thank the referee for this observation. It is correct that cannot conclude from our data that Cyclin B-CDK1 is first activated in the nucleus. We mention this as a possibility in the Discussion only, based on recently published data (Kapadia and Nurse, Nature, 2025). However, we should point out that this is not an absolute requirement for our model. Activation of the cyclin B1-CDK1 complex can occur in the cytoplasm, but impairment in nuclear translocation of the complex imposed by disrupting chromosome condensation would still result in a delay in mitotic entry. In support of this hypothesis, it was previously shown that low levels of cyclin B1-CDK1 activity are sufficient to induce its translocation into the nucleus (Gavet and Pines, Dev. Cell, 2010). We have now also discussed the potential role of cyclin A in these events and how they could crosstalk with cyclin B1. We have clarified these points in the revised manuscript. Additional experiments involving cel cycle regulators are outlined in the next comment.

2 - "…the major concern here is that the authors never measure the activity of the cell cycle regulators - essential for them to conclude anything about control of the timing of mitotic entry".

"A second, related, concern, is that the author assume that their perturbations of the nucleus act on the mitotic trigger directly; they do not consider that they may have activated checkpoint pathways that delay mitotic entry".

These comments represent valid concerns raised by the referee. To address both points, we have now synchronized cells and performed an analysis of cell-cycle regulators in cells with disrupted chromosome condensation. We quantified the levels of Wee1 (Heald et al., 1993; Masuda et al., MBoC, 2011), p21 (Hervé et al., Nat Cell Biol, 2025) and p38 (Mikhailov et al., Curr Biol, 2007). Moreover, we assessed the levels of phospho-CDK1 Y15 (Heald et al., Cell, 1993; McGowan and Russel, EMBO J, 1993), that corresponds to an inactive form of the kinase. These results have been added to the manuscript in a new Fig. 4 and have been discussed accordingly. Based on our results, we concluded that interfering with chromosome condensation leads to an increase in the nuclear levels of Wee1, which maintain CDK1 in an inactive state (as assessed by the increased levels of CDK1 Y15). These observations are in line with the observed delay in mitotic entry. On the other hand, we observed no differences in the levels of either p21 or p38, suggesting that we are not activating the antephase checkpoint that would induce a G2 delay (Matsusaka and Pines, 2004). How chromosome condensation crosstalks with Wee1 is a very interesting follow-up that remains to be determined.

3 - "The study is entirely confined to HeLa cells, which have a perturbed cell cycle due to their aneuploidy and expression of HPV E6 and E7 proteins".

This is yet another accurate observation made by the referee. Nevertheless, we would like to point out that by using a synchronization protocol we are forcing cells to start the process of mitotic entry from a roughly similar stage, which should minimize effects due to cell cycle perturbations. Under these conditions, our control HeLa cells are significantly different than our treated cells, which should allow us to conclude that the treatments have a specific effect on mitotic entry. Nevertheless, to address the referee´s concern, we have now repeated the analysis of cyclin B1 translocation in near-diploid, untransformed RPE-1 cells expressing cyclin B1-eYFP that were treated with VPA. These experiments have confirmed our earlier observations with HeLa cells and have been added to the revised manuscript in Fig. EV3.

4 - "The authors measure the timing of mitotic entry by release from a RO3306 arrest. RO3306 inhibits CDK1 and prolonged arrest alters the cell cycle machinery because it inhibits Cyclin A-CDK1 activity as well as preventing Cyclin B-CDK1 activity".

Again, a very valid concern raised by the referee. We would like to point out that our control cells, which are also treated with the CDK1 inhibitor quickly enter mitosis after the release (16.1±14.1 min), while treated cells are significantly delayed (Fig. 2 and Fig. EV3 in our manuscript). Nevertheless, to rule out a synergistic deleterious effect of CDK1 inhibition with the other treatments, we have now used a double thymidine synchronization protocol and measured cyclin B1 translocation in either control cells or cells with disrupted chromosome condensation. This has provided us with an independent way to assess the effect of chromosome condensation on the timing of mitotic entry. As can be seen in revised Fig. 2, disruption of chromosome condensation by treatment with ICRF-193 or VPA also significantly delays mitotic entry in cells synchronized with a double thymidine block.

5 - "The authors assume that the primary actions of ICR-193, valproic acid, and SMC2 depletion are through their effect on chromosome condensation, but this is the only effect they measure and so remains correlation not causation".

We thank the referee for this comment. Our assumption on the action of these treatments regarding chromosome condensation is based on extensive published literature (e.g., Ishida et al., J Cell Bio, 1994; Biggs et al., MBoC, 2019; Hirota et al., J Cell Sci, 2004; Schneider et al., Nature, 2022). These treatments were selected to target the multiple components involved in the chromosome condensation process. We confirmed their impact on chromosome condensation by measuring the coefficient of variation (CV) parameter. Although we cannot formally rule out potential side-effects of these treatments, we believe it is unlikely that these side-effects would lead to the same delay in mitotic entry. In any case, we have revised the text to indicate that we observe a correlation between condensation and the delay.

6 - "The authors emphasise the recent paper from the Nurse lab reporting activation of Cyclin B-CDK1 in the nucleus in support of their conclusion that Cyclin B-CDK1 is activated in the nucleus in human cells. The problem here is that cdc13-CDK1 in fission yeast is nuclear, whereas human Cyclin B-CDK1 is cytoplasmic".

This is a relevant point already raised by the referee in a previous comment (please see point 1 above). We would like to emphasize that the nuclear activation of cyclin B1-CDK1 was just one possible hypothesis suggested in the discussion, which was not formally tested. In the revised manuscript, we have now taken into consideration the possible role of cyclin A in the initial steps of chromosome condensation (Gong & Ferrell, Mol Biol Cell, 2010) and in the activation of the cyclin B1-CDK1 complex (De Boer et al., Oncogene, 2008; Hegarat et al., EMBO J, 2020) and discussed our results in light of this. We avoided considerations regarding the nuclear activation of cyclin B1, which was not tested and is not the main focus of this manuscript.

Response to Referee #2

"I am very excited by the findings and the topic but found the paper quite difficult to read and understand -the writing could be streamlined and improved for clarity without contradicting or hinting at interpretations. Importantly, from the abstract onwards, the manuscript is vaguely written with respect to precisely describing the mechanical state of the nuclei that the authors aim to implicate - I would suggest rewriting for precision and clarity statements like "mitotic entry is driven by a nuclear mechanical signal", "spatiotemporally integrated by SUN proteins on the nuclear envelope", "chromosome-generated mechanical signal ", "mitotic onset is controlled by a chromosome-dependent nuclear mechanism", even in figures/results sessions there are vague titles like "Nuclear changes in prophase are determined by chromosome condensation" or "Chromosome condensation generates a mechanical signal on the nucleus". Please define your "mechanical signal" - given that the authors measure NE fluctuation, nuclear pore dilation, and look at cPLA2 they mean to say NE tension from the onset - why not just call it what it is?"

We thank the referee for this insightful comment and suggestions and apologize for the lack of clarity. We have now worked hard to clarify the ambiguous sentences throughout the manuscript and specifically refer the events analysed.

"Conceptually I do not think that compression is a good model for chromosome-condensation driven increase in NE tension relevant for the biological process at hand - it doesn't really recapitulate the G2-M transition which involves an increase in volume and thus completely different concentration balance scenario when compared to compression which leads to nuclear volume loss".

This is a relevant point raised by the referee, although we observe that compression does rescue the timing of mitotic entry. Nevertheless, to resolve this issue, we have now used a hypotonic treatment that triggers an increase in nuclear volume (e.g. Deviri and Safran, PNAS, 2022) and should recapitulate what we observe in prophase cells (Fig. 1G, H). Importantly, we previously showed that hypotonic treatment increases NE tension (Dantas et al., J Cell Biol, 2022). To this end, we have disrupted chromosome condensation by treating cells with ICRF-193 or VPA, incubated cells in a hypotonic solution and filmed them during mitotic entry. This was sufficient to trigger cyclin B1 translocation and rescue the delay imposed by disrupting chromosome condensation. These results have been added to a revised Fig. 5. In addition, we have performed hypotonic treatment in cells expressing DHC-GFP and depleted of SUN2 RNAi to confirm our results with the cell confiner. These results have been added to a revised Fig. 6.

“I understand the NE tension but the authors already have a nice paper showing tension is required - to me the novelty here is where the process starts which the author don't state specifically but rather dance around the pool of cyclin B1/CDK1 come from with contradictory discussions.”

We apologize for the lack of clarity. To avoid confusion, we have rewritten the text to focus on the role of chromosome condensation as the source of the downstream nuclear changes required for mitotic entry. We have focused the discussion on the contribution of chromosome condensation for the process of mitotic entry, instead of addressing the possible role of the nuclear pool of cyclin B1/CDK1.

“NPC dilation and transport discussion is very confusing - am I wrong to conclude that the authors believe that the initial cyclin B1/CDK1 event occurs in the nucleus or is it that the NE tension/NE unfolding increases NPC dilation and thus promotes faster transport of cyclin B1/CDK1 to the nucleus? Is there a pool of cyclin B1/CDK in the nucleus? Did the authors show or discuss this? I am not sure this was experimentally shown so it is difficult to understand in the current form what exactly the message is from the authors. You might consider making a clear point on this as it is an important detail.”

Again, we apologize for the lack of clarity. Based on our data, we believe that an increase in tension leads to NE unfolding and NPC dilation, which promotes faster transport of cyclin B1. Regarding the referee´s comment on the nuclear pool of cyclin B1 (which is shared by referee #1), we did not directly measure the nuclear activity of cyclin B1-CDK1. As we also mentioned in our response to referee #1, this was just one possible hypothesis suggested in the discussion, which was not formally tested. In the revised manuscript, we avoid considerations regarding the nuclear activation of cyclin B1, which was not tested and is not the focus of this manuscript. Instead, we take into consideration the role of cyclin A in the initial steps of chromosome condensation (Gong & Ferrell, Mol Biol Cell, 2010) and in the activation of the cyclin B1-CDK1 complex (De Boer et al., Oncogene, 2008; Hegarat et al., EMBO J, 2020) and discussed our results accordingly.

“What is the link between cPLA2 activation and opening of NPCs - model is proposed but exact link unclear? Or do the authors use it just a measure of NE tension?”

Based on previous works, we use cPLA2 recruitment to the NE simply as a proxy for NE tension (Lomakin et al., Science, 2020; Enyedi et al., Cell, 2016; Dantas et al., J Cell Biol, 2022). Also based on a recent report, NE tension was shown to be sufficient to dilate NPCs (Zimmerli et al., Science, 2021). In our model, we propose that increased NE tension during prophase, which we confirm by the recruitment of cPLA2 to the NE, drives NPC dilation. In agreement with this hypothesis, we observed a decrease in cPLA2 recruitment to the NE when chromosome condensation was disrupted (Fig. 3C-E, 3G).

“In figure 5, siLMNA for figure 5B the chosen nucleus is a raisin when looking at nuclear shape/lamin B then then the chosen nucleus to examine dynein loading in figure 5D is quite normal looking - again we need to see a lot more than one nucleus for all of the quantifications and there is likely a lot of variability”.

We thank the referee for the careful analysis. Indeed, there is a significant variability in nuclear shape upon Lamin A depletion, as can be observed by scattering of the values in the "nuclear irregularity index" (NII) plot (revised figure 6C). To clarify this point, we have added an additional example to the figure, as well as to Fig. EV4B, to better reflect the variability in nuclear shapes.

"I find the SUN1/2 inhibition data a bit puzzling as it has the same effect under control and confined conditions so it is really tension-independent then this really makes the story again confusing - please reason here.

"In my view it also appears surprising that SUN proteins would be required for the force transmission given the huge conformational change of the chromatin with condensation. Maybe elaborate a bit, perhaps even graphically how the authors see the temporal evolution of force balancing across the INM/ONM and all the players involved before/during/after mitotic commitment and/or chromosome condensation - describing this 15min process clearly graphically would help".

We thank the referee for this valuable comment. Based on the results obtained after SUN1/2 depletion, we propose the following model (new Fig. 8).

1 - During G2, when chromosomes are not condensed, the forces acting on the NE are low. Therefore, NE membrane displacements are low (Fig. 1A-F) and cPLA2 is not recruited to the NE (Fig. 3C, D).

2 - As cells progress towards mitosis, forces acting on the NE change. There is an increase in both inwards- and outwards-directed NE membrane fluctuations. Whereas inwards displacements seem to depend on chromosome condensation (Fig. 2B) (Introini et al., Commun Biol, 2023), outwards displacements are microtubule-dependent (Fig. EV1) Together with the observed increase in nuclear volume (Fig. 1H), increased perinuclear space (Fig. 1N) and cPLA2 recruitment to the NE (Fig. 1K), they reflect a modification in the mechanical state of the nucleus, essential to ensure the timing of mitotic entry.

3 – Disrupting this force balance changes NE tension. Interfering with chromosome structure leads to a change in NE membrane displacements (Fig. 2B), decreased NPC dilation and perinuclear space (Fig. 1I, J), and lower cPLA2. Depletion of SUN proteins also changes the way in which forces are transmitted to the NE, reflected by a decrease in cPLA2 levels on the NE (Fig. EV4E, F). Moreover, both depletion of SUN2 (Fig. 6K, L) and expression of a dominant-negative KASH (DN-KASH), impair cyclin B1 translocation (Dantas et al., 2022), which can be rescued by mechanical stimulation (Dantas et al., J Cell Biol, 2022), indicating this is a LINC complex-dependent process.

We also propose that SUN proteins act in a LINC complex-independent way to transmit the forces generated by chromosomes directly to NPCs. It is known that at least SUN1 directly associates with NPCs (Liu et al., 2007; Talamas and Hetzer, 2010). Moreover, SUN-dependent forces can position NPCs (Smith et al., 2022) and generate "tension islands" on the NE that can drive NPC dilation (Morgan et al., 2025). Based on our observations, we also know that cells depleted of SUN proteins are unable to load CENP-F (Fig. 7E-H) or dynein on NPCs even after confinement or hypotonic treatment (Fig. 6J-M). Therefore, we reasoned SUN proteins could be responsible for transmitting forces from chromosomes to the NE, generating local areas of tension concentrated in or around NPCs. Indeed, we have previously shown that during prophase, SUN proteins

concentrate on specific areas of the NE close to centrosomal microtubules (Lima et al., Life Sci Alliance, 2024). This localized tension could alter NE or NPC structure in way that is not recapitulated by the confinement setup or hypotonic treatment, possibly explaining the lack of dynein rescue in SUN depleted cells that are compressed. We have now tried to clarify these aspects in the revised text. We have also modified our graphical model to include a proposed temporal order of events and the forces acting on the NE.

"Can the authors reason why they believe their mechanism is really the NE tension versus nuclear unfolding during prophase? What were the experiments that uncoupled these two? What authors do show is that chromosome condensation is required for NE unfolding/tension and NPC dilation and that compression induces dynein loading to NE (they have already showed the cyclinB and tension part earlier) but then they go on to discuss chromosome-NE interaction and how this is critical - again without really showing or focusing on this aspect in the work - these discussions distract from the main points. I reason this is where the authors are going?"

Regarding this point, we would like to clarify that we do not intend to separate the contribution of unfolding from tension. We think NE tension arises from an unfolding of NE membranes during this stage, given the observed increase in the recruitment of cPLA2. This is similar to what we and others have previously shown (Lomakin et al., Science, 2020; Enyedi et al., Cell, 2016; Dantas et al., J Cell Biol, 2022). Moreover, in a previous publication we also observed that increasing NE folding by LBR overexpression leads to a decrease in NE tension in RPE-1 cells (Dantas et al., J Cell Biol, 2022). We have clarified these points in the manuscript and avoided generalizations regarding chromosome-NE interactions.

"Is it possible to detect/image condensin activity in the nucleus and time it to cyclinB1/CDK1 activation to address where the initial signal comes from (ie peak of condensin activity first or cyclin B1/CDK1?)"

It is not possible to directly measure condensin activity *per se* in our experimental conditions. However, it is known that condensin activity (and therefore chromosome condensation) requires CDK activity. In fact, both cyclin A (Gong and Ferrell, MBoC, 2010) and CDK1 (Abe et al., Genes and Dev, 2011) are required to start the condensation process. Considering these reports and comments made by other referees, we have now rewritten our discussion to focus on the possible role of cyclin A as the initial trigger for chromosome condensation and cyclin B1-CDK1 activation. Notably, during late G2, cyclin A localizes to the nucleus, placing it in the right context to mediate these processes.

"Please define DHC-GFP somewhere before using it. DHC-GFP that is quantified in 6E is missing a representative image"

DHC-GFP has now been defined in the text. I´m afraid we could not find a quantification of DHC-GFP in 6E. We assume the referee meant 5E? We have now added a representative image of control cells expressing DHC-GFP along with an example of a cell depleted of Lamin A. These are now in a revised Fig. 6D.

“I would suggest introducing CENP-F and discussion on nuclear export of CENP-F, allowing the loading of dynein on the NE, earlier in the introduction - in general introduce all the players at the onset so that then the logic is clear. I would also suggest modifying your diagram in figure 5 (why is plectin there pictured!?) and bringing it into the intro or similar”.

We have now added a section to the Introduction of the revised manuscript focusing on the loading of dynein on the NE. We included plectin in the diagram for the sake of completion, since nesprins can bind multiple cytoskeletal filaments. However, we agree that is irrelevant in the context of this manuscript. We have now modified the diagram in Fig. 5 and left only microtubules in the scheme.

“For all figures it is necessary to provide many more than 1 image to be able to access the robustness of the phenotype especially since the authors are analyzing 20-30cells. It appears that all the statistics were done improperly using cell numbers and not n numbers - cannot use cells to calculate significance; please correct”

We apologize for the lack of clarity. We have now provided additional images to some of the key figures. Whenever lower cell numbers are presented, these are related to experiments that are technically very challenging, combining live-cell analysis with depletions and confinement. For these reasons, it is very difficult to obtain large cell numbers. Moreover, working with mitosis is particularly challenging, considering that any undue stress to the cell will inevitably result in a cell cycle arrest. For most experiments, the number of cells measured was usually between 30-50 cells per condition. These cell numbers are within what is routinely used in the field. We have also defined the number of replicates, included all datapoints and clarified the statistical tests. All experiments were independently replicated a minimum of three times.

“Given the reputation of the authors, the data behind all direct experimental observations and interpretations are exciting and if the authors believe there is a pool of nucleoplasmic cyclinB1/CDK1 and that the process starts in the nucleus, that is new - I would review cell numbers which are quite low and statistics should be redone and all replicas means shown (no boxplots should be used with all data point hidden but the outliers) with a few exceptions I pointed to above but I must say the manuscript is not written in a way that is helpful to the message - it is not always clear, especially to a non-expert, why the vast majority of experiments were done - there are too many players and too many "axis" of regulation without direct evidence for cyclin B1-CDK1 being activated in the nucleus in the first place at the onset, which may or may not be proposed to be the case. I think the story is too complicated - condensing chromatin, SUN proteins, Nesprins, NUPs, cPLA2, cyclin B1-CDK, dynein, BicD2, Nup133/CENP-F/NudE/EL pathway - everything is involved - it is really hard to digest this paper and if the authors feel it is important to include all of them then a rewrite session will be helpful.”

Again, we apologize for the lack of clarity. We have now changed the graphs to include all data points. We also show replicate means and updated all statistical tests.

Moreover, as we mentioned above, we have rewritten the manuscript to improve clarity and simplify the message.

Response to Referee #3

"Cdk1 activation gradually increases throughout G2 phase, but in this experimental setup Cdk1 is inhibited through a prolonged G2, which affects transcription through G2 and the balance of kinase activities when Cdk1 inhibition is removed to start mitotic entry. Thus, the starting point of the experiments may not necessarily reflect the situation when mitosis starts during unperturbed proliferation. Key results need to be validated independent of synchronization by Cdk1 inhibition".

We thank the referee for this relevant comment, which is shared by referee #1. Using synchronizations is a convenient way to ensure that cells are starting from a similar state. This also provides us with a reference point to start our measurements. To clarify that the delays we see are not due to unbalanced kinase activities, we have now assessed mitotic entry using a double thymidine synchronization protocol. This allowed us to confirm that prolonged Cdk1 inhibition is not negatively impacting the dynamics of the process. These results were been added to a revised Fig. 2. We have also analysed the levels of specific cell cycle regulators such as Wee1, p21, p38 and CDK pY15. These results have been added to a new Fig. 4.

"The manuscript focuses only on Cyclin B-Cdk1. Cyclin A-Cdk1 (and Cdk2) and its partial cytoplasmic translocation in G2 should at least be discussed. The role of Cyclin A should in particular be mentioned in the discussion of how the spatial feedback is initiated."

We thank the reviewer for pointing out the relevant role of cyclin A for mitotic entry. We have addressed this point in the discussion.

"In case the connection between chromosome condensation and Cyclin B-Cdk1 translocation is driving mitotic entry, a prediction could be that blocking chromosome condensation would delay mitotic entry even when artificially activating Cdk1. Would inhibition of chromosome condensation delay mitotic entry forced by Wee1 inhibition?"

This is a very interesting suggestion by the referee. We should point out that the biochemical pathway mediated by cyclin B1-CDK1 is essential for mitotic entry. Indeed, inhibition of CDK1 blocks mitotic entry even when cyclin B1 is forced to enter the nucleus by confinement (Dantas et al., J Cell Biol, 2022). Following the referee`s comment, we have now assessed the nuclear levels of Wee1 after blocking chromosome condensation (Fig. 4A). We have also quantified the timing of mitotic entry in cells treated with VPA and the specific Wee1 inhibitor MK-1775. These results were added to a revised Fig. 4E, F and discussed in the text.

Dear Dr. Ferreira,

Thank you again for submitting your revised manuscript EMBOJ-2025-122546R, "Chromosome condensation mechanically primes the nucleus for mitosis." We have now received a complete set of reviews from all referees, which you will find enclosed below for your information. They all acknowledge the improvements to the study, and so we should be able to pursue it further for publication. Nevertheless, as I mentioned yesterday, we are trying to sort out a disagreement between the referees from the mitotic regulation side:

As you will see below, referee 1 remains unconvinced that the conclusion re. nuclear membrane tension affection initiation of mitosis via cyclin B1 import is strongly supported. I in turn discussed this caveat with referee 3, who has a similar expertise and provided a somewhat more balanced view on this aspect (see also below). In essence, referee 3 felt that some of your rescue data would provide more than just correlative evidence, but also noted that the exact mechanistic connections -and particularly the relative importance of certain pathways- remain to be worked out in the future.

At this point, I would invite you to consider these referee opinions, and think about how they could best be incorporated into the presentation of the final manuscript in ways that would acknowledge the remaining uncertainties and present specific conclusions in a sufficiently circumspect manner so as to also show consideration for referee 1's hesitations.

No need to re-revise the text at this point, simply respond to me with some thoughts/ideas, and once we agreed on a good way forward, I would then send you a formal invitation for final revisions.

Thanks in advance and looking forward to hearing from you,

Best regards,

Hartmut

Hartmut Vodermaier, PhD
Senior Editor, The EMBO Journal
h.vodermaier@embojournal.org

Referee #1 (Report for Author)

In this revised version the authors have taken account of my criticisms and addressed most of them experimentally where needed. They have included new data on the level of WEE1 and phospho-Y15 on CDK1 to address my concern that they had not measured the cell cycle regulators in their study. These data show that WEE1 levels are increased along with a consequent increase in P-Y15 when chromosome condensation is perturbed. The authors interpret this as evidence supporting the idea that chromosome condensation primes the nucleus for mitosis but I would argue that it shows that, as I originally surmised, their manipulations are triggering a checkpoint response that is delaying mitosis. Thus, I am still of the opinion that the authors have not established that chromosome condensation is an upstream trigger for mitosis.
The authors have shifted their discussion to suggest that Cyclin A might be responsible for chromosome condensation and triggering mitosis, which is in agreement with many studies showing that Cyclin A is required for cells to enter mitosis, and more pertinently the study by Furuno et al., 1999, which showed that inhibiting Cyclin A activity in prophase will reverse chromosome condensation and send cells back to interphase.
In summary, this paper reports interesting, high quality data on the mechanics of nuclear membrane tension at mitosis that warrant publication, but I remain unconvinced that they have shown that this is has a direct effect on the initiation of mitosis through regulating Cyclin B1 import. I would support publication of the paper only if they remove this conclusion.

Referee #2 (Report for Author)

Authors addressed all of my concerns and improved the clarity and data presentation in the manuscript - i have no further comments of questions

Referee #3 (Report for Author)

The authors have addressed my points and I think the revised manuscript has improved. I have no remaining concerns.

Additional comments re. ref 1:

I can see the reasoning of the reviewer - how do we know that these treatments do not simply invoke a checkpoint. The key experiment for me here is that they can rescue the

treatment. If this is merely an artefact of the treatments, presumably acting on Cdk activity, then it is not surprising that Wee1 inhibition could overrule it. But blocking condensation and rescuing Cyclin B translocation by increasing tension in my mind points to a model in which tension driven by chromosome condensation can influence Cyclin B localisation.

Admittedly, there is only one experiment that forces Cyclin B translocation after VPA and ICRF treatment (5B, C), and other rescues using confinement and assessing DHC are possibly more thorough. As stated by the authors, their treatments could have other effects, and hypotonic shock used for Cyclin B translocation certainly could do more than increase nuclear membrane tension.

Nonetheless, I believe that taken together, the manuscript shows a mechanistic connection to translocation of Cyclin B. This connection is likely to be modulated, and given that Cdk influences both chromosome condensation and cyclin B translocation, we clearly do not understand everything here. The relative importance of this pathway, compared to other described modes of Cdk/localization and activation is in my mind not that clear.

In my view the discussion section in the revised version is more balanced in the revised version and brings up that we do not understand the full link to Cdk activity. I would also argue that it is difficult to separate certain aspects of checkpoints from normal progression, as ATR, CHK1, WEE1 are active and regulate cell cycle progression in the absence of external perturbation. Given that nuclear membrane topology can affect ATR I would not be surprised if checkpoint proteins are also involved.

Thus, I don't see the need to remove statements that chromosome condensation can affect nuclear membrane tension, which in turn can affect Cyclin B localization. What can be modulated is in my mind exactly how important this pathway will be for mitotic entry.

Dr. Hartmut Vodermaier
EMBO Journal

20th May 2026

Manuscript: EMBOJ-2025-122546

Dear Dr. Vodermaier,

thank you for your feedback and for your handling of this process. I agree with the referees that there are additional mechanistic connections that need to be figured out regarding the crosstalk between chromosome condensation and cell cycle progression, particularly regarding cyclin B1 activation.

One such aspect is related to how nuclear envelope (NE) tension affects the timing of mitotic entry. Admittedly, as referee 3 points out, in the current manuscript there is only one experiment that forces cyclin B1 translocation after disruption of chromosome condensation (Fig. 5B, C). Nevertheless, I would also like to point out that in a previous publication (Dantas et al., J. Cell Biol., 2022), we have thoroughly characterized how disruption of NE tension (without interfering with chromosome condensation) was sufficient to impair cyclin B1 translocation and delay mitotic entry. Moreover, this effect was reverted by confinement of the nucleus. Overall, these observations are suggestive that cyclin B1 localization is, at least partly, sensitive to NE tension.

After carefully reading all the observations made by the referees, we propose to modify the discussion of the manuscript to reflect their comments. In particular, we will clarify that: (1) there are multiple pathways that regulate CDK activity and localization, and (2) chromosome condensation-dependent regulation of NE tension is one of the possible regulatory mechanisms of mitotic entry. Moreover, we will also reinforce that it is still unclear how chromosome condensation crosstalks with the G2-M checkpoint to regulate the timing of mitotic entry. With this, we will avoid framing chromosome condensation as the single or main driver of this process.

We hope that these changes will be sufficient to reassure you and the referees, allowing publication of our manuscript in EMBO Journal.

A01/00

INSTITUTO DE INVESTIGAÇÃO E INOVAÇÃO EM SAÚDE
UNIVERSIDADE DO PORTO

Rua Alfredo Allen, 208
4200-135 Porto
Portugal
+351 220 408 800
info@i3s.up.pt
www.i3s.up.pt

We remain available to further clarify these matters with you, either by email or via a remote meeting. Do let us know if there is anything else that is required on our side.

With our warmest regards

Jorge G. Ferreira, *PhD*
Project Leader at Instituto de Investigação e Inovação em Saúde (i3S)
Associate Professor at the Faculty of Medicine of Porto
Rua Alfredo Allen, 208 / 4200-135 Porto, Portugal
Tel.: +351 220 408 800 Ext. 6011 / Email: jferreir@ibmc.up.pt

INSTITUTO DE INVESTIGAÇÃO E INOVAÇÃO EM SAÚDE
UNIVERSIDADE DO PORTO

Rua Alfredo Allen, 208
4200-135 Porto
Portugal
+351 220 408 800
info@i3s.up.pt
www.i3s.up.pt

Prof. Jorge G. Ferreira
Instituto de Investigação e Inovação em Saúde -i3S
Epithelial Polarity and Cell Division
Rua Alfredo Allen
Porto 4200-315
Portugal

21st May 2026

Re: EMBOJ-2025-122546R
Chromosome condensation mechanically primes the nucleus for mitosis

Dear Dr. Ferreira,

Thank you again for submitting your revised manuscript to our editorial office. In light of the overall positive assessment of your revisions by the three original referees, we would be happy to move towards acceptance and publication of this work in The EMBO Journal, as soon as a few remaining editorial issues have been addressed:

- In light of the remaining caveat raised by referee 1, and referee 3's additional perspective on this concern, please modify the discussion of the manuscript to reflect these comments, as proposed.

- Our pre-acceptance figure checks indicated that some of the representative images in Figure 3 are displayed in both panels A and C. Please make sure to clearly state/explain this in the figure legend, or choose different representative images for the two occasions.

- Please adjust the order of the manuscript sections, and also make sure to use the correct section headers:
Title page with complete author information, Abstract, Introduction, Results, Discussion, Methods, Data Availability, Acknowledgements, Disclosure and Competing Interests Statement, References, Main Figure Legends, Tables, Expanded Figure Legends.

- Please carefully go through the reference list, which contains several entries lacking full citation information such as volume or page/eLocator numbers.

- As we are switching from a free-text author contribution statement towards a more formal statement based on Contributor Role Taxonomy (CRediT) terms, please remove the present Author Contribution section and instead specify each author's contribution(s) directly in the Author Information page of our submission system during upload of the final manuscript. See https://casrai.org/credit/ for more information.

- Please cut the EV movie legend from the main text, instead placing it into a separate legend text file; then move the legend file together with the respective movie file into a separate ZIP archive before re-uploading as "Movie EV1".

- Please provide suggestions for a short 'blurb' text prefacing and summing up the conceptual aspect of the study in two sentences (max. 250 characters), followed by 3-5 one-sentence 'bullet points' with brief factual statements of key results of the paper; they will form the basis of an editor-written 'Synopsis' accompanying the online version of the article. Please also upload a synopsis image, which can be used as a "visual title" for the synopsis section of your paper. The image (maybe based on a simplified version of Figure 8?) should be ideally in JPG format, and please make sure that it remains in the modest dimensions of (exactly) 550 pixels wide and between 300 pixels high.

- Finally, during routine pre-acceptance checks, our data editors have raised the following queries regarding figures, data, and legends; I would appreciate if you briefly answered to them in the cover letter of your final submission, and made the requested text modifications with changes/additions highlighted via the "Track changes" option, to facilitate our final checking"
1. Please state the exact p valuesin the legends of figures 1D, E, M, N; EV1 A, B; EV2 G, EV4 D, EV5 B, C, E, F, I, J
2. Please indicate the statistical test used for data analysis in the legends of figures 1D, E, H, I, K, M, N; 2B, D, G, I, K; 3B, D, F, G, I, J; 4B, D, F, H, J; 5C, I, K; 6C, E, G, I, K, M; EV1 A, B, C; EV2 B, D, E, F, G; EV3 H, J; EV4 D, G, I; EV5 B, C, E, F, I, J
3. Please provide the missing information related to "n" in the legends of figures 1E, I; 3D, G; 7B, D, G, H, J, L, M; EV1 C
4. Please define the error bars in the legends of figures 1E, H, I, K, M, N; 2B, D, G, I, K; 3B, D, F, G, I, J; 4B, D, F, H, J; 5C, I, K; 6C, E, G, I, K, M; 7B, D, G, H, J, L, M;EV1 C; EV2 B, D, E, F; EV3 H, J; EV4 D, G, I; EV5 B, C, E, F, I, J

I am returning the manuscript to you for a final round of minor revision, to allow you to make these modifications and upload the revised files. Once we will have received them, we should be ready to swiftly proceed with formal acceptance and production of the manuscript.

With kind regards,

Hartmut

Hartmut Vodermaier, PhD
Senior Editor, The EMBO Journal
h.vodermaier@embojournal.org

*** PLEASE NOTE: All revised manuscript are subject to initial checks for completeness and adherence to our formatting guidelines. Revisions may be returned to the authors and delayed in their editorial re-evaluation if they fail to comply to the following requirements. As a first step please read our guidelines for revised submissions:
https://link.springer.com/journal/44318/submission-guidelines#cms-Revised-submissions

1) Every manuscript requires a Data Availability section (even if only stating that no deposited datasets are included). Primary datasets or computer code produced in the current study have to be deposited in appropriate public repositories prior to resubmission, and reviewer access details provided in case that public access is not yet allowed.

2) Each figure legend must specify
- size of the scale bars that are mandatory for all micrograph panels
- the statistical test used to generate error bars and P-values
- the type error bars (e.g., S.E.M., S.D.)
- the number (n) and nature (biological or technical replicate) of independent experiments underlying each data point
- Figures may not include error bars for experiments with n<3; scatter plots showing individual data points should be used instead.

3) Revised manuscript text (including main tables, and figure legends for main and EV figures) has to be submitted as editable text file (e.g., .docx format). We encourage highlighting of changes (e.g., via text color) for the referees' reference.

4) Each main and each Expanded View (EV) figure should be uploaded as individual production-quality files (preferably in .eps, .tif, .jpg formats). For suggestions on figure preparation/layout, please refer to our Figure Preparation Guidelines:
https://media.springernature.com/original/springer-cms/rest/v1/content/27825798/data/v1

5) Point-by-point response letters should include the original referee comments in full together with your detailed responses to them (and to specific editor requests if applicable), and also be uploaded as editable (e.g., .docx) text files.

6) Please complete our Author Checklist, and make sure that information entered into the checklist is also reflected in the manuscript; the checklist will be available to readers as part of the Review Process File.

7) All authors listed as (co-)corresponding need to deposit, in their respective author profiles in our submission system, a unique ORCiD identifier linked to their name. Please see our Guide to Authors for detailed instructions.

8) Please note that supplementary information at EMBO Press has been superseded by the 'Expanded View' for inclusion of additional figures, tables, movies or datasets; with up to five EV Figures being typeset and directly accessible in the HTML version of the article.

9) To facilitate reproducibilty and cross-laboratory adoption of methodologies, please structure the Materials & Methods section as outlined in our guide to authors, including a completed Reagents and Tools Table.

10) Digital image enhancement is acceptable practice, as long as it accurately represents the original data and conforms to community standards. If a figure has been subjected to significant electronic manipulation, this must be clearly noted in the figure legend and/or the 'Materials and Methods' section. The editors reserve the right to request original versions of figures and the original images that were used to assemble the figure. Finally, we generally encourage uploading of numerical as well as gel/blot image source data.

At EMBO Press we ask authors to provide source data for the main manuscript figures. You will receive a separate email with instructions for providing source data with your revised manuscript, including how to upload and organize the files.

In the interest of ensuring the conceptual advance provided by the work, we recommend submitting a revision within 3 months (19th Aug 2026). Please discuss the revision progress ahead of this time with the editor if you require more time to complete the revisions. Use the link below to submit your revision:

Dr. Hartmut Vodermaier
EMBO Journal

28th May 2026

Manuscript: EMBOJ-2025-122546

Dear Dr. Vodermaier,

We would like to thank you for your feedback on our manuscript entitled "Chromosome condensation mechanically primes the nucleus for mitosis".

We have now addressed the final points raised by the referees and the editorial office. All changes to the manuscript were highlighted using "Track Changes" to facilitate your checking. Below you can find a point-by-point response to the comments.

- We have modified the discussion to reflect the changes suggested by the referees. In that regard we reinforced that multiple pathways regulate CDK activity and localization and indicated that chromosome condensation-dependent regulation of NE tension is one of the regulatory mechanisms of mitotic entry.
- We have altered the representative images of DMSO and VPA in panel 3A, which were inadvertently also present in panel 3C.
- We have adjusted the order of manuscript sections and the respective headings
- We have checked and corrected the reference list to include pages/eLocator numbers.
- Author contributions were removed from the manuscript and updated on the submission page.
- The legend for Movie EV1, along with the actual movie, are supplied as a separate ZIP archive.

INSTITUTO
DE INVESTIGAÇÃO
E INOVAÇÃO
EM SAÚDE
UNIVERSIDADE
DO PORTO

Rua Alfredo Allen, 208
4200-135 Porto
Portugal
+351 220 408 800
info@i3s.up.pt
www.i3s.up.pt

- We have provided a separate file with a short "blurb" text and one-sentence bullet points, as well as a synopsis image.
- We have added the exact p values in the figure legends. However, we should note that we are not able to provide exact values whenever the $p<0.0001$, since our statistical analysis software (GraphPad Prism) caps p values to this limit.
- We have indicated all statistical tests used in the figure legends.
- We have provided "n" in the figure legends. Some of the requests were already in the manuscript. We highlighted these in yellow.
- We have defined what the plots represent, including the error bars for all figures.

Please do not hesitate in contacting me, should you have any doubts. I look forward to hearing from you at your earliest convenience.

With my warmest regards,

Jorge Ferreira

Jorge G. Ferreira, *PhD*
Project Leader at Instituto de Investigação e Inovação em Saúde (i3S)
Associate Professor at the Faculty of Medicine of Porto
Rua Alfredo Allen, 208 / 4200-135 Porto, Portugal
Tel.: +351 220 408 800 Ext. 6011 / Email: jferreir@ibmc.up.pt

INSTITUTO
DE INVESTIGAÇÃO
E INOVAÇÃO
EM SAÚDE
UNIVERSIDADE
DO PORTO

Rua Alfredo Allen, 208
4200-135 Porto
Portugal
+351 220 408 800
info@i3s.up.pt
www.i3s.up.pt

EMBO Press Author Checklist

Corresponding Author Name: Jorge G. Ferreira	
Journal Submitted to: The EMBO Journal	
Manuscript Number: EMBOJ-2025-122546R	

USEFUL LINKS FOR COMPLETING THIS FORM
The EMBO Journal - Author Guidelines
EMBO Reports - Author Guidelines
Molecular Systems Biology - Author Guidelines
EMBO Molecular Medicine - Author Guidelines

Reporting Checklist for Life Science Articles (updated January
This checklist is adapted from Materials Design Analysis Reporting (MDAR) Checklist for Authors. MDAR establishes a minimum set of requirements in transparent reporting in the life sciences (see Statement of Task: 10.31222/osf.io/9sm4x). Please follow the journal's guidelines in preparing your
Please note that a copy of this checklist will be published alongside your article.

Abridged guidelines for figures
1. Data
The data shown in figures should satisfy the following conditions:
- the data were obtained and processed according to the field's best practice and are presented to reflect the results of the experiments in an accurate and unbiased manner.
- ideally, figure panels should include only measurements that are directly comparable to each other and obtained with the same assay.
- plots include clearly labeled error bars for independent experiments and sample sizes. Unless justified, error bars should not be shown for technical
- if n<5, the individual data points from each experiment should be plotted. Any statistical test employed should be justified.
- Source Data should be included to report the data underlying figures according to the guidelines set out in the authorship guidelines on Data

2. Captions
Each figure caption should contain the following information, for each panel where they are relevant:
- a specification of the experimental system investigated (eg cell line, species name).
- the assay(s) and method(s) used to carry out the reported observations and measurements.
- an explicit mention of the biological and chemical entity(ies) that are being measured.
- an explicit mention of the biological and chemical entity(ies) that are altered/varied/perturbed in a controlled manner.
- the exact sample size (n) for each experimental group/condition, given as a number, not a range;
- a description of the sample collection allowing the reader to understand whether the samples represent technical or biological replicates (including how many animals, litters, cultures, etc.).
- a statement of how many times the experiment shown was independently replicated in the laboratory.
- definitions of statistical methods and measures:
  - common tests, such as t-test (please specify whether paired vs. unpaired), simple χ2 tests, Wilcoxon and Mann-Whitney tests, can be unambiguously identified by name only, but more complex techniques should be described in the methods section;
  - are tests one-sided or two-sided?
  - are there adjustments for multiple comparisons?
  - exact statistical test results, e.g., P values = x but not P values < x;
  - definition of 'center values' as median or average;
  - definition of error bars as s.d. or s.e.m.

Please complete ALL of the questions below.
Select "Not Applicable" only when the requested information is not relevant for your study.

Materials

Newly Created Materials	Information included in the manuscript?	In which section is the information available? (Reagents and Tools Table, Materials and Methods, Figures, Data Availability Section)
New materials and reagents need to be available; do any restrictions apply?	Yes	Reagents and Tools Table, Materials and Methods, Data Availability Section

Antibodies	Information included in the manuscript?	In which section is the information available? (Reagents and Tools Table, Materials and Methods, Figures, Data Availability Section)
For **antibodies** provide the following information:<br>- Commercial antibodies: RRID (if possible) or supplier name, catalogue number and or/clone number<br>- Non-commercial: RRID or citation	Yes	Reagents and Tools Table, Materials and Methods

DNA and RNA sequences	Information included in the manuscript?	In which section is the information available? (Reagents and Tools Table, Materials and Methods, Figures, Data Availability Section)
**Short novel DNA or RNA including primers, probes**: provide the sequences.	Yes	Reagents and Tools Table, Materials and Methods

Cell materials	Information included in the manuscript?	In which section is the information available? (Reagents and Tools Table, Materials and Methods, Figures, Data Availability Section)
**Cell lines:** Provide species information, strain. Provide accession number in repository **OR** supplier name, catalog number, clone number, and/**OR** RRID.	Yes	Reagents and Tools Table, Materials and Methods
**Primary cultures:** Provide species, strain, sex of origin, genetic modification status.	Not Applicable	
Report if the cell lines were recently **authenticated** (e.g., by STR profiling) and tested for mycoplasma contamination.	Yes	Materials and Methods

Experimental animals	Information included in the manuscript?	In which section is the information available? (Reagents and Tools Table, Materials and Methods, Figures, Data Availability Section)
**Laboratory animals or Model organisms:** Provide species, strain, sex, age, genetic modification status. Provide accession number in repository **OR** supplier name, catalog number, clone number, **OR** RRID.	Not Applicable	
**Animal observed in or captured from the field:** Provide species, sex, and age where possible.	Not Applicable	
Please detail **housing and husbandry conditions**.	Not Applicable	

Plants and microbes	Information included in the manuscript?	In which section is the information available? (Reagents and Tools Table, Materials and Methods, Figures, Data Availability Section)
**Plants:** provide species and strain, ecotype and cultivar where relevant, unique accession number if available, and source (including location for collected wild specimens).	Not Applicable	
**Microbes:** provide species and strain, unique accession number if available, and source.	Not Applicable	

Human research participants	Information included in the manuscript?	In which section is the information available? (Reagents and Tools Table, Materials and Methods, Figures, Data Availability Section)
If collected and within the bounds of privacy constraints report on age, sex and gender or ethnicity for all study participants.	Not Applicable	

Core facilities	Information included in the manuscript?	In which section is the information available? (Reagents and Tools Table, Materials and Methods, Figures, Data Availability Section)
If your work benefited from core facilities, was their service mentioned in the acknowledgments section?	Yes	Acknowledgments

Design

Study protocol	Information included in the manuscript?	In which section is the information available? (Reagents and Tools Table, Materials and Methods, Figures, Data Availability Section)
If study protocol has been **pre-registered, provide DOI in the manuscript**. For clinical trials, provide the trial registration number **OR** cite DOI.	Not Applicable	
Report the **clinical trial registration number** (at ClinicalTrials.gov or equivalent), where applicable.	Not Applicable	

Laboratory protocol	Information included in the manuscript?	In which section is the information available? (Reagents and Tools Table, Materials and Methods, Figures, Data Availability Section)
Provide DOI OR other citation details if **external detailed step-by-step protocols** are available.	Not Applicable	

Experimental study design and statistics	Information included in the manuscript?	In which section is the information available? (Reagents and Tools Table, Materials and Methods, Figures, Data Availability Section)
Include a statement about **sample size** estimate even if no statistical methods were used.	Yes	Materials and Methods
Were any steps taken to minimize the effects of subjective bias when allocating animals/samples to treatment (e.g. **randomization procedure**)? If yes, have they been described?	Not Applicable	
Include a statement about **blinding** even if no blinding was done.	Yes	Materials and Methods
Describe **inclusion/exclusion criteria** if samples or animals were excluded from the analysis. Were the criteria pre-established?<br><br>If sample or data points were omitted from analysis, report if this was due to attrition or intentional exclusion and provide justification.	Not Applicable	
For every figure, are **statistical tests** justified as appropriate? Do the data meet the assumptions of the tests (e.g., normal distribution)? Describe any methods used to assess it. Is there an estimate of variation within each group of data? Is the variance similar between the groups that are being statistically compared?	Yes	Materials and Methods

Sample definition and in-laboratory replication	Information included in the manuscript?	In which section is the information available? (Reagents and Tools Table, Materials and Methods, Figures, Data Availability Section)
In the figure legends: state number of times the experiment was **replicated** in laboratory.	Yes	Figure Legends
In the figure legends: define whether data describe **technical or biological replicates**.	Yes	Figure Legends

Ethics

Ethics	Information included in the manuscript?	In which section is the information available? (Reagents and Tools Table, Materials and Methods, Figures, Data Availability Section)
Studies involving **human participants**: State details of **authority granting ethics approval** (IRB or equivalent committee(s), provide reference number for approval.	Not Applicable	
Studies involving **human participants**: Include a statement confirming that **informed consent** was obtained from all subjects and that the experiments conformed to the principles set out in the WMA Declaration of Helsinki and the Department of Health and Human Services Belmont Report.	Not Applicable	
Studies involving **human participants:** For publication of **patient photos**, include a statement confirming that consent to publish was obtained.	Not Applicable	
Studies involving experimental **animals**: State details of **authority granting ethics approval** (IRB or equivalent committee(s), provide reference number for approval. Include a statement of compliance with ethical regulations.	Not Applicable	
Studies involving **specimen and field samples:** State if relevant **permits** obtained, provide details of authority approving study; if none were required, explain why.	Not Applicable	

Dual Use Research of Concern (DURC)	Information included in the manuscript?	In which section is the information available? (Reagents and Tools Table, Materials and Methods, Figures, Data Availability Section)
Could your study fall under dual use research restrictions? Please check biosecurity documents and list of **select agents and toxins** (CDC): https://www.selectagents.gov/sat/list.htm	Not Applicable	
If you used a select agent, is the security level of the lab appropriate and reported in the manuscript?	Not Applicable	
If a study is subject to dual use research of concern regulations, is the name of the **authority granting approval and reference number** for the regulatory approval provided in the manuscript?	Not Applicable	

Reporting

The MDAR framework recommends adoption of discipline-specific guidelines, established and endorsed through community initiatives. Journals have their own policy about requiring specific guidelines and recommendations to complement MDAR.

Adherence to community standards	Information included in the manuscript?	In which section is the information available? (Reagents and Tools Table, Materials and Methods, Figures, Data Availability Section)
State if relevant guidelines or checklists (e.g., **ICMJE, MIBBI, ARRIVE, PRISMA**) have been followed or provided.	Not Applicable	
For **tumor marker prognostic studies,** we recommend that you follow the **REMARK** reporting guidelines (see link list at top right). See author guidelines, under 'Reporting Guidelines'. Please confirm you have followed these guidelines.	Not Applicable	
For **phase II and III randomized controlled trials**, please refer to the **CONSORT** flow diagram (see link list at top right) and submit the CONSORT checklist (see link list at top right) with your submission. See author guidelines, under 'Reporting Guidelines'. Please confirm you have submitted this list.	Not Applicable	

Data Availability

Data availability	Information included in the manuscript?	In which section is the information available? (Reagents and Tools Table, Materials and Methods, Figures, Data Availability Section)
Have **primary datasets** been deposited according to the journal's guidelines (see 'Data Deposition' section) and the respective accession numbers provided in the Data Availability Section?	Yes	Data Availability Section
Were **human clinical and genomic datasets** deposited in a public access-controlled repository in accordance to ethical obligations to the patients and to the applicable consent agreement?	Not Applicable	
Are **computational models** that are central and integral to a study available without restrictions in a machine-readable form? Were the relevant accession numbers or links provided?	Not Applicable	
If publicly available data were reused, provide the respective **data citations in the reference list**.	Not Applicable	
