# Supplementary material for: Chromosome condensation mechanically primes the nucleus for mitosis
Source: EMBO J. 2026 Jun 29;45(15):5357–87. doi: 10.1038/s44318-026-00835-8 (PMC13434721; doi:10.1038/s44318-026-00835-8)
Supplement: Supplementary file 2 [file 44318_2026_835_MOESM3_ESM.pdf]

Expanded View Figures

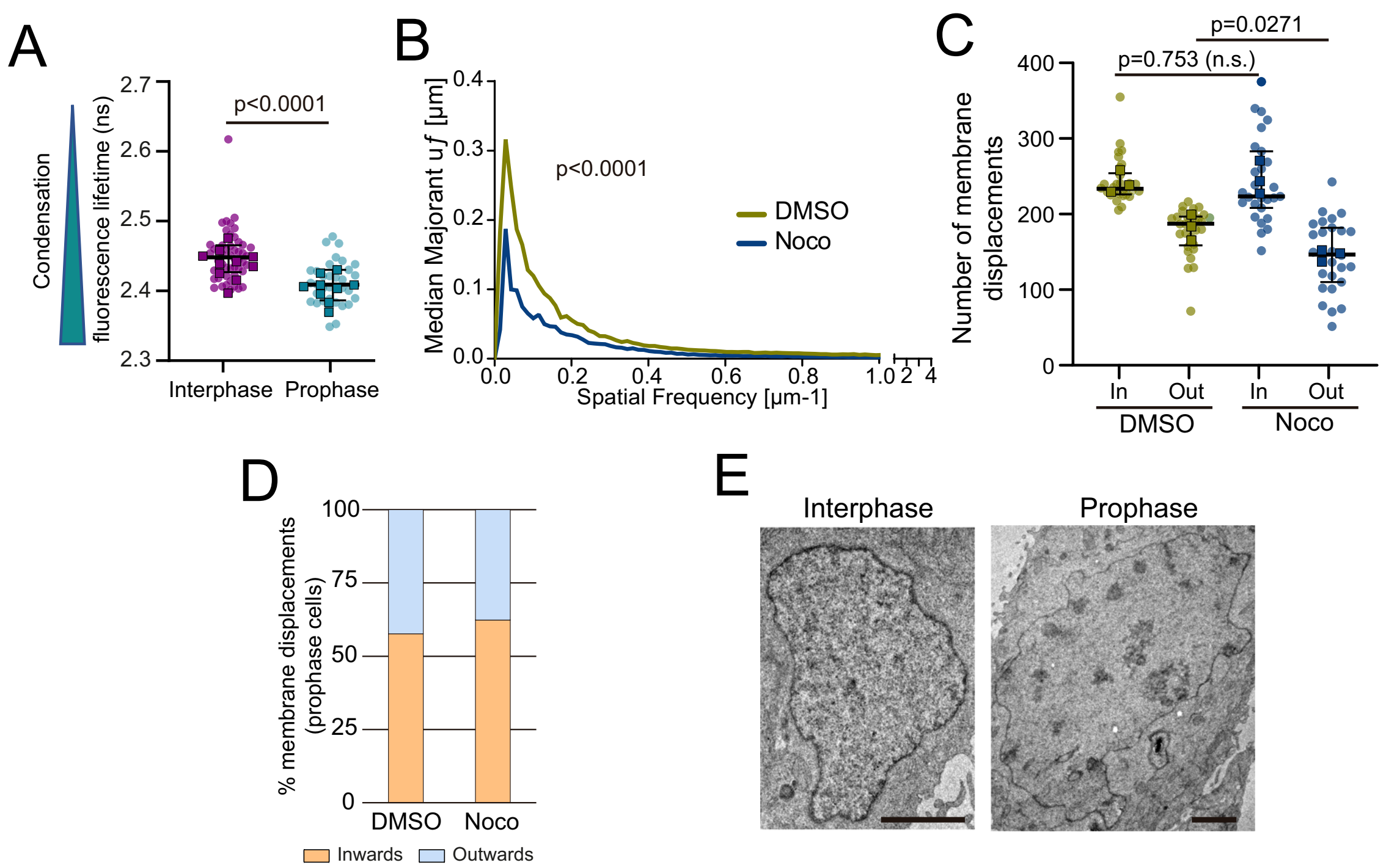

Figure EV1. Microtubule-dependent changes in NE membrane displacement.

(**A**) Fluorescence lifetime microscopy (FLIM) measurement of histone H4-GFP of cells in interphase ($n = 50$ cells, 3 replicates) and prophase ($n = 36$ cells, 3 replicates, $P < 0.0001$, using a Kruskal–Wallis test). Decreased fluorescence lifetime in prophase cells reflects increased chromosome compaction. (**B**) Median of the majorant of frequency-dependent NE membrane displacements, uf, obtained by finding the maximum amplitude of the spatial Fourier transform (FT) for each frequency for DMSO ($n = 30$ cells, 3 replicates) and Nocodazole treated cells (Noco; $n = 27$ cells, 3 replicates; $P < 0.0001$, using a two-way ANOVA). This FT curve shows the maximum displacement amplitude of each wavelength. Quantification of the orientation (**C**) and proportion (**D**) of NE membrane displacements for DMSO ($n = 30$ cells, 3 replicates) and Nocodazole treated cells ($n = 27$ cells, 3 replicates; $P = 0.0271$, using an unpaired t test). (**E**) Representative TEM images of interphase (left) and prophase (right) nuclei. On the dot plots, each dot corresponds to an individual value, the line corresponds to the median and the bars correspond to the interquartile range. Square dots represent the median of each replicate in the experiment. Scale bars correspond to 5 µm.

 © The Author(s)

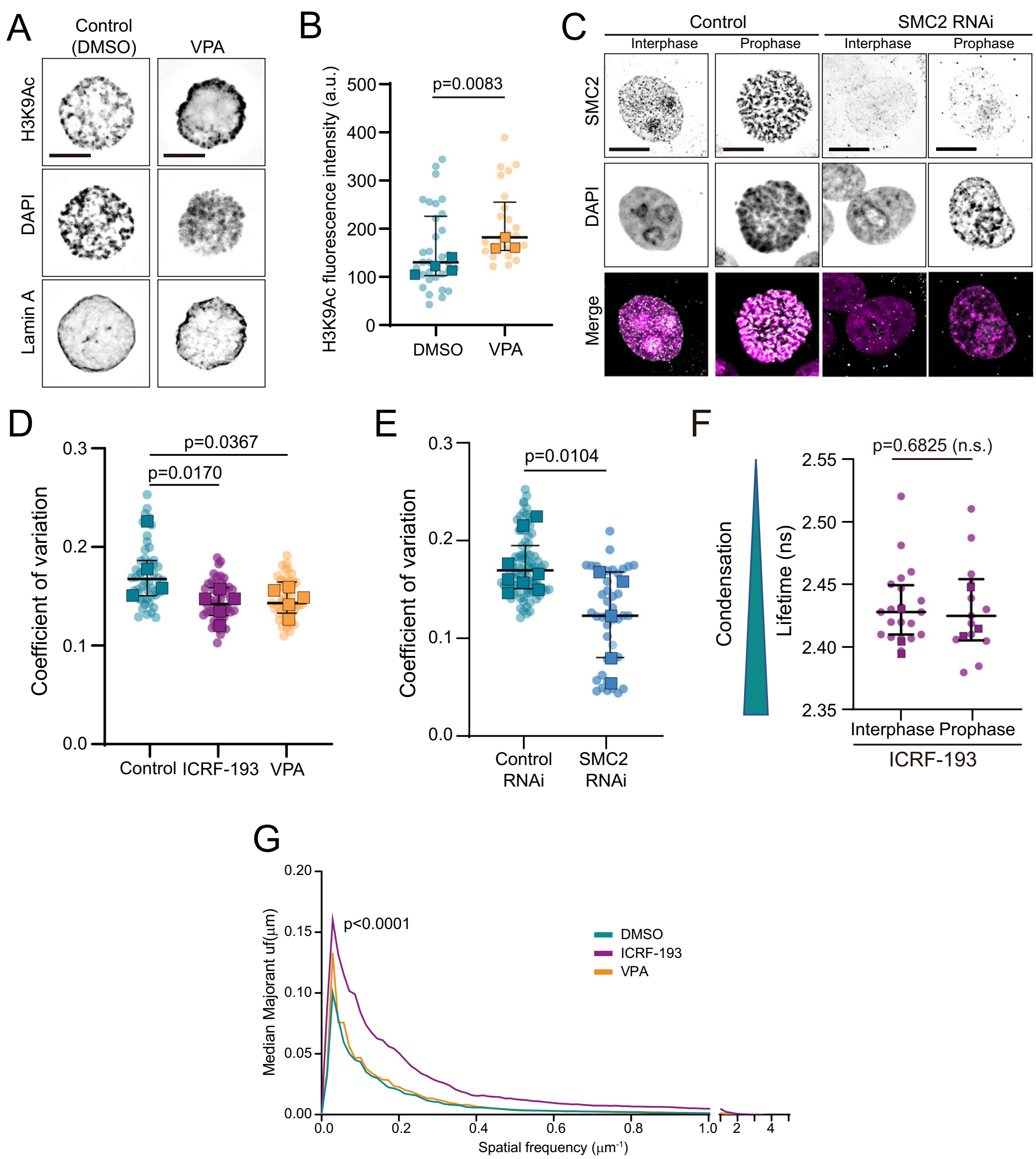

Figure EV2. Disrupting chromosome condensation affects NE membrane displacements.

(**A**) Representative immunofluorescence images of histone H3 acetylation on lysine 9 (H3K9ac) in control cells (DMSO) and VPA-treated cells. (**B**) Quantification of H3K9ac levels in prophase cells treated with DMSO ($n = 33$ cells, 4 replicates) or VPA ($n = 25$ cells, 3 replicates for VPA; $P = 0.0083$, using an unpaired t test), to determine the efficacy of VPA treatment in increasing histone acetylation. (**C**) Representative images of SMC2 immunostaining in control cells and SMC2-depleted cells in interphase and prophase, to demonstrate the efficacy of the RNAi treatment. (**D**) Quantification of chromosome condensation using the coefficient of variation (CV) parameter. Treatment with ICRF-193 ($n = 49$ cells, 5 replicates, $P = 0.0170$) or VPA ($n = 48$ cells, 5 replicates, $P = 0.0367$) significantly decreases the CV when compared to control cells ($n = 52$ cells, 4 replicates), reflecting an impairment in chromosome condensation. Comparisons between groups were performed using an ANOVA test. (**E**) Quantification of chromosome condensation after depletion of SMC2 by RNAi. The CV is significantly decreased in depleted cells ($n = 42$ cells, 5 replicates; $P = 0.0104$, using an unpaired t test) when compared to control cells ($n = 91$ cells, 8 replicates). (**F**) FLIM measurement of histone H4-GFP in interphase or prophase cells treated with ICRF-193 ($n = 20$ cells for interphase and $n = 13$ cells for prophase; $p = 0.6825$ using a Mann–Whitney test). Decreased fluorescence lifetime corresponds to increased chromosome compaction. (**G**) Median of the majorant of frequency-dependent NE membrane displacements, uf, obtained by finding the maximum amplitude of the spatial Fourier transform (FT) for each frequency for DMSO ($n = 27$ cells), ICRF-193 ($n = 30$ cells) or VPA-treated cells ($n = 22$ cells; $P < 0.0001$, using a Friedman test). This FT curve shows the maximum displacement amplitude of each wavelength. On the dot plots, each dot corresponds to an individual value, the line corresponds to the median and the bars correspond to the interquartile range. Square dots represent the median of each replicate in the experiment.

© The Author(s)

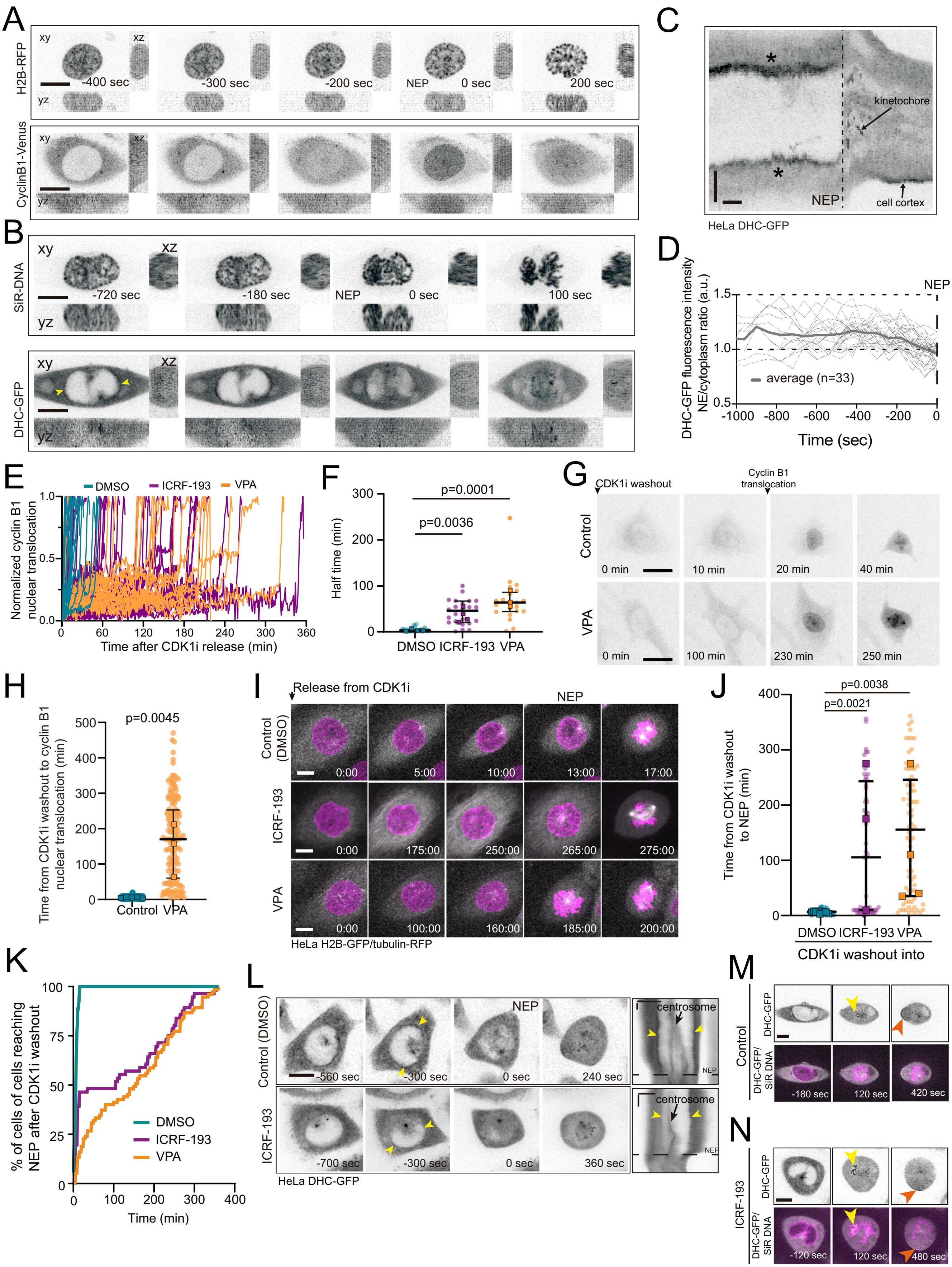
A
H2B-RFP
CyclinB1-Venus
xy
xz
yz
-400 sec
-300 sec
-200 sec
NEP
0 sec
200 sec
B
SiR-DNA
DHC-GFP
-720 sec
-180 sec
100 sec
C
kinetochore
cell cortex
HeLa DHC-GFP
D
DHC-GFP fluorescence intensity NE/cytoplasm ratio (a.u.)
average (n=33)
Time (sec)
E
DMSO
ICRF-193
VPA
Normalized cyclin B1 nuclear translocation
Time after CDK1i release (min)
F
Half time (min)
p=0.0001
p=0.0036
G
CDK1i washout
Cyclin B1 translocation
Control
0 min
10 min
20 min
40 min
100 min
230 min
250 min
H
Time from CDK1i washout to cyclin B1 nuclear translocation (min)
p=0.0045
Control
I
Release from CDK1i
Control (DMSO)
HeLa H2B-GFP/tubulin-RFP
J
Time from CDK1i washout to NEP (min)
p=0.0038
p=0.0021
CDK1i washout into
K
% of cells of cells reaching NEP after CDK1i washout
Time (min)
L
centrosome
-560 sec
-300 sec
240 sec
-700 sec
360 sec
HeLa DHC-GFP
M
DHC-GFP/ SiR DNA
-180 sec
120 sec
420 sec
N
-120 sec
480 sec

Figure EV3. Chromosome condensation sets the timing of mitotic entry.

(**A**) Representative frames from a movie of a HeLa cell expressing cyclin B1-Venus/H2B-RFP during mitotic entry. (**B**) Representative frames from a movie of a HeLa cell expressing DHC-GFP and stained with SiR-DNA during mitotic entry. Note the accumulation of dynein on the NE (yellow arrowheads). (**C**) Kymograph of the cell in (**B**), highlighting the accumulation of dynein on the NE prior to NEP (asterisks), as well as its accumulation on kinetochores and cell cortex after mitotic entry. Horizontal scale bar, 200 s. Vertical scale bar, 10 µm. (**D**) Ratio between dynein on the NE and on the cytoplasm over time in prophase cells ($n = 33$ cells). Note how the ratio is above 1, reflecting an enrichment on the NE at this stage. In the movies, time zero corresponds to the moment of NEP. (**E**) Individual traces for cyclin B1 nuclear translocation for cells treated with DMSO, ICRF-193 or VPA. Time zero corresponds to the time of CDK1 inhibitor (CDK1i) washout. Values were normalized and scaled between 0 and 1, corresponding to the minimum and maximum values, respectively. (**F**) Half-times for cyclin B1 translocation were calculated from the curve fits for DMSO, ICRF-193 ($P = 0.0036$) and VPA ($P = 0.0001$). Comparisons between groups were performed using an ANOVA test. (**G**) Representative frames from movies of RPE-1 cells expressing cyclin B1-eYPF after synchronization with a CDK1 inhibitor. Following washout, cells were filmed during mitotic entry. Time lapse is 5 min. Time is in min and scale bars are 100 µm. (**H**) Quantification of the time from CDK1i washout to cyclin B1 nuclear translocation in RPE-1 cells ($n = 232$ cells, 5 replicates for DMSO; $n = 149$ cells, 3 replicates for VPA; $P = 0.0045$, using an unpaired *t* test). (**I**) Representative frames from movies of cells expressing H2B-GFP/tubulin-RFP that were synchronized in late G2 using CDK1i and incubated with DMSO, ICRF-193 or VPA overnight. Following CDK1i washout, cells progressed to prophase, in medium still containing either DMSO, ICRF-193 or VPA. Time zero corresponds to washout of CDK1i. (**J**) Quantification of the time from CDK1i washout to NEP for DMSO ($n = 70$ cells, 9 replicates), ICRF-193- ($n = 84$ cells, 3 replicates, $P = 0.0021$) or VPA-treated cells ($n = 85$ cells, 4 replicates, $P = 0.0038$). Comparisons between groups were performed using an ANOVA test. NEP is determined as the moment soluble tubulin enters the nuclear space, reflecting permeabilization of the NE. (**K**) Cumulative percentage of cells treated with DMSO, ICRF-193 or VPA that enter mitosis (NEP) after CDK1i washout. (**L**) Representative frames of HeLa cells expressing DHC-GFP cells during mitotic entry, after treatment with DMSO (top panel) or ICRF-193 (bottom panel). Yellow arrowheads indicate the NE. Right panels show kymographs highlighting the absence of dynein on the NE of ICRF-193 treated cells. Horizontal scale bar, 10 µm. Vertical scale bar, 200 s. Representative frames from movies of cells expressing DHC-GFP and stained with SiR-DNA from control cells (**M**) and ICRF-193 (**N**) treated cells. Note how dynein still accumulates at kinetochores (yellow arrowhead) and cell cortex (orange arrowhead) after ICRF-193 treatment. On the dot plots, each dot corresponds to an individual value, the line corresponds to the median and the bars correspond to the interquartile range. Square dots represent the median of each replicate in the experiment.

© The Author(s)

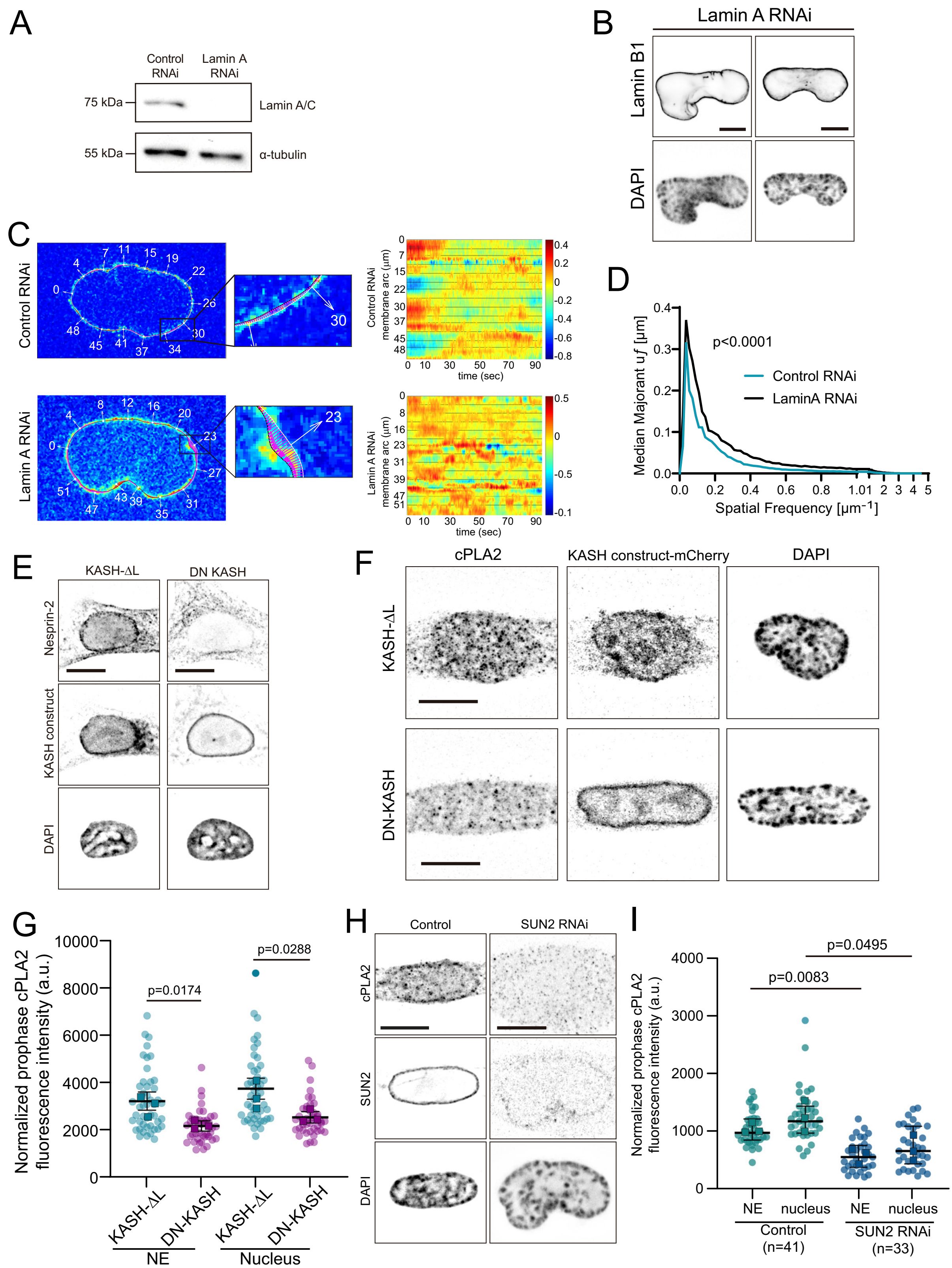
A
Control RNAi
Lamin A RNAi
75 kDa
55 kDa
Lamin A/C
α-tubulin
B
Lamin A RNAi
Lamin B1
DAPI
C
Control RNAi
Lamin A RNAi
Control RNAi membrane arc (μm)
Lamin A RNAi membrane arc (μm)
time (sec)
D
p<0.0001
Control RNAi
LaminA RNAi
Median Majorant uf [μm]
Spatial Frequency [μm-1]
E
KASH-ΔL
DN KASH
Nesprin-2
KASH construct
DAPI
F
cPLA2
KASH construct-mCherry
DAPI
KASH-ΔL
DN-KASH
G
Normalized prophase cPLA2 fluorescence intensity (a.u.)
p=0.0174
p=0.0288
KASH-ΔL
DN-KASH
NE
Nucleus
H
Control
SUN2 RNAi
cPLA2
SUN2
DAPI
I
Normalized prophase cPLA2 fluorescence intensity (a.u.)
p=0.0495
p=0.0083
NE
nucleus
Control (n=41)
SUN2 RNAi (n=33)

 © The Author(s)

Figure EV4. Tension on the NE requires SUN proteins.

(**A**) Western blotting analysis of Lamin A levels following depletion by RNAi. (**B**) Representative images of nuclei from cells depleted of Lamin A. (**C**) Representative examples of inwards (pink vectors) or outwards (red vectors) NE membrane displacements of a control cell (top) and a cell depleted of Lamin A (bottom). Right panels show a map of the displacement amplitude, u, obtained for all time frames. The *y* axis corresponds to the arc around the median NE membrane. (**D**) Median of the majorant of frequency-dependent displacements, uf, obtained by finding the maximum amplitude of the spatial Fourier transform (FT) for each frequency for controls ($n = 24$ cells) and Lamin A depleted cells ($n = 21$ cells; $P < 0.0001$, using a two-way ANOVA). This FT curve shows the maximum displacement amplitude of each wavelength. (**E**) Representative immunofluorescence images of the expression of KASH-ΔL (left panel) and DN-KASH (right panel) constructs in HeLa cells and their respective impact on Nesprin-2 localization on the NE. (**F**) Representative immunofluorescence images of cPLA2 levels on the nucleus in cells expressing KASH-ΔL or DN-KASH. (**G**) Quantification of cPLA2 fluorescence intensity on the NE and nucleus for cells expressing KASH-ΔL or DN-KASH ($n = 46$ cells, 3 replicates for KASH-DL; $n = 42$ cells, 3 replicates for DN-KASH, $P = 0.0174$ for NE and $P = 0.0288$ for nucleus). Group comparisons were performed using unpaired *t* tests. (**H**) Representative immunofluorescence images of cPLA2 levels on the nucleus following SUN2 RNAi. (**I**) Quantification of cPLA2 fluorescence intensity on the NE and nucleus for control cells ($n = 41$ cells, 3 replicates) and SUN2 depleted cells ($n = 33$ cells, 3 replicates; $P = 0.0083$ for NE and $P = 0.0495$ for nucleus). Comparisons between groups were performed using unpaired *t* tests. On the dot plots, each dot corresponds to an individual value, the line corresponds to the median and the bars correspond to the interquartile range. Square dots represent the median of each replicate in the experiment. Scale bars, 10 µm.

© The Author(s)

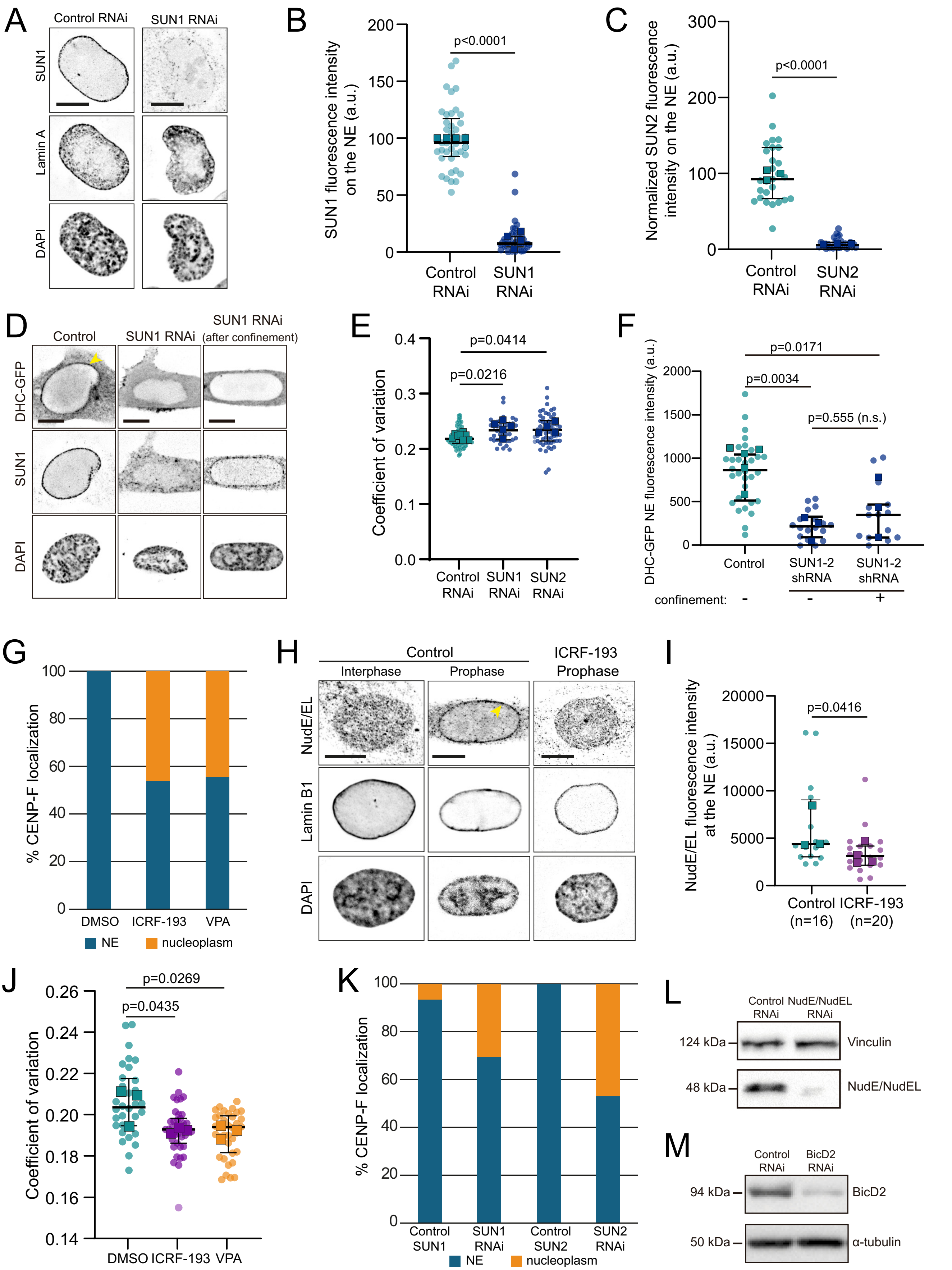
A
Control RNAi
SUN1 RNAi
SUN1
Lamin A
DAPI
B
SUN1 fluorescence intensity on the NE (a.u.)
p<0.0001
Control RNAi
SUN1 RNAi
C
Normalized SUN2 fluorescence intensity on the NE (a.u.)
p<0.0001
Control RNAi
SUN2 RNAi
D
Control
SUN1 RNAi
SUN1 RNAi (after confinement)
DHC-GFP
SUN1
DAPI
E
Coefficient of variation
p=0.0414
p=0.0216
Control RNAi
SUN1 RNAi
SUN2 RNAi
F
DHC-GFP NE fluorescence intensity (a.u.)
p=0.0171
p=0.0034
p=0.555 (n.s.)
Control
SUN1-2 shRNA
SUN1-2 shRNA
confinement: - - +
G
% CENP-F localization
DMSO
ICRF-193
VPA
NE
nucleoplasm
H
Control
Interphase
Prophase
ICRF-193 Prophase
NudE/EL
Lamin B1
DAPI
I
NudE/EL fluorescence intensity at the NE (a.u.)
p=0.0416
Control (n=16)
ICRF-193 (n=20)
J
Coefficient of variation
p=0.0269
p=0.0435
DMSO
ICRF-193
VPA
K
% CENP-F localization
Control SUN1
SUN1 RNAi
Control SUN2
SUN2 RNAi
NE
nucleoplasm
L
Control RNAi
NudE/NudEL RNAi
124 kDa
Vinculin
48 kDa
NudE/NudEL
M
Control RNAi
BicD2 RNAi
94 kDa
BicD2
50 kDa
α-tubulin

 © The Author(s)

Figure EV5. SUN proteins regulate dynein loading through the Nup133-CENP-F pathway.

(**A**) Representative immunofluorescence images of SUN1, Lamin A and DAPI for control cells (left panel) and SUN1 depleted cells (right panel). (**B**) Quantification of SUN1 levels on the NE of controls ($n = 41$ cells, 4 replicates) and SUN1 RNAi cells ($n = 46$ cells, 4 replicates, $P < 0.0001$, using an unpaired t test). (**C**) Quantification of SUN2 levels on the NE of control cells ($n = 27$ cells, 3 replicates) and SUN2 RNAi cells ($n = 30$ cells, 3 replicates; $P < 0.0001$, using an unpaired t test). (**D**) Representative immunofluorescence images showing dynein and SUN1 localization on the NE for control cells and SUN1-depleted cells. (**E**) Analysis of chromosome condensation using the coefficient of variation (CV) for control cells ($n = 104$ cells, 8 replicates) and cells treated with SUN1 RNAi ($n = 40$ cells, 5 replicates; $P = 0.0216$) or SUN2 RNAi ($n = 60$ cells, 4 replicates; $P = 0.0414$). Comparisons between groups were performed using an ANOVA test. Depletion of SUN proteins leads to a small but significant increase in CV, reflecting increased chromosome condensation. (**F**) Quantification of dynein fluorescence intensity on the NE for controls ($n = 32$ cells, 5 replicates) and SUN1/SUN2 shRNA without ($n = 18$ cells, 3 replicates; $P = 0.0034$) or with confinement ($n = 15$ cells, 3 replicates; $P = 0.0171$). Note how confinement does not restore dynein on the NE after SUN1/SUN2 are depleted ($P = 0.555$). Comparisons between groups were performed using an ANOVA test. (**G**) Quantification of the percentage of prophase cells showing NE or nucleoplasmic localization of CENP-F for controls, ICRF-193- or VPA-treated cells. (**H**) Immunofluorescence analysis of NudE/EL localization on the NE after treatment with ICRF-193. In control prophase cells, NudE/EL localize to the NE (yellow arrowhead). This localization is lost after treatment with ICRF-193 (right panel). (**I**) Quantification of NudE/EL fluorescence intensity on the NE after treatment with ICRF-193 ($n = 20$ cells, 4 replicates; $P = 0.0416$, using an unpaired t test), in comparison with control cells ($n = 16$ cells, 3 replicates). (**J**) Quantification of the coefficient of variation (CV) for control cells (DMSO, $n = 32$ cells, 3 replicates) and cells treated with ICRF-193 ($n = 38$ cells, 3 replicates, $P = 0.0435$) or VPA ($n = 35$ cells, 3 replicates, $P = 0.0269$). Comparisons between groups were performed using an ANOVA test. (**K**) Quantification of the percentage of prophase cells showing NE or nucleoplasmic localization of CENP-F in control cells, SUN1-depleted or SUN2-depleted cells. (**L**) Representative western blotting analysis of BicD2 levels in control cells and BicD2-depleted cells. (**M**) Representative western blotting analysis of NudE/EL levels in control cells and NudE/EL-depleted cells. On the dot plots, each dot corresponds to an individual value, the line corresponds to the median and the bars correspond to the interquartile range. Square dots represent the median of each replicate in the experiment.

© The Author(s)
