# Supplementary material for: Chromosome condensation mechanically primes the nucleus for mitosis
Source: EMBO J. 2026 Jun 29;45(15):5357–87. doi: 10.1038/s44318-026-00835-8 (PMC13434721; doi:10.1038/s44318-026-00835-8)
Supplement: Supplementary file 3 [file 44318_2026_835_MOESM2_ESM.zip › Legend for Movie EV1.docx]

**Legend for Movie EV1 – Chromosome condensation controls the nuclear translocation of cyclin B1**

Representative movie of HeLa cells expressing cyclin B1-Venus after treatment with DMSO (control), ICRF-193 or VPA. Cells were previously synchronized in G2 using a CDK1 inhibitor (Ro-3306). Movie starts immediately after inhibitor washout (time zero). Time lapse is in min and scale bar is 10 μm. Note the severe delay in cyclin B1 translocation after treatment with either ICRF-193 or VPA.
